# Deciphering the initiation pathway of CagT4SS assembly by in vitro reconstitution of subcomplexes

**DOI:** 10.1038/s41467-026-75820-0

**Published:** 2026-07-23

**Authors:** Hoi Yee Chu, Chin Yu Mok, You-Rong Lin, Huawei Zhang, Takayuki Uchihashi, Shannon Wing Ngor Au

**Affiliations:** 1 Center for Protein Science and Crystallography, School of Life Sciences, The Chinese University of Hong Kong, Shatin, Hong Kong, China; 2 Exploratory Research Center of Life and Living Systems, National Institutes of Natural Sciences, Okazaki, Aichi Japan; 3 Center for AI-Driven Medical Research, Shenzhen Institutes of Advanced Technology, Chinese Academy of Sciences, Shenzhen, China; 4 Department of Physics, Graduate School of Science, Nagoya University, Nagoya, Aichi Japan; 5 Institute for Glyco-core Research (iGCORE), Nagoya University, Nagoya, Japan; 6 Quantum-Based Frontier Research Hub for Industry Development (Q-BReD), Nagoya University, Nagoya, Aichi Japan

**Keywords:** Atomic force microscopy, Cryoelectron microscopy

## Abstract

Cag type IV secretion system (CagT4SS) mediates the translocation of diverse substrates and is critical for the *Helicobacter pylori* pathogenesis. Its assembly is initiated by the central cylinder comprising CagX, CagY and CagM, however, the order and detailed mechanism remain unclear. Here, we characterize their respective self-oligomerizing properties and in vitro reconstitute subcomplexes that recapitulate features of the native nanomachine. By integrating multiple biophysical analyses, the structures, binding kinetics and conformational dynamics of assembly intermediates are shown. The assembly begins with self-oligomerization of CagX, followed by CagY association to form a stable periplasmic ring complex (PRC). CagM is subsequently coupled with CagX through multimerization of dimers, with CagY enhancing both the stability and cooperativity of its association. Our findings show the spatial and temporal nature of the assembly process, in particular the significance of PRC substructure, providing mechanistic insights into the biogenesis of bacterial T4SSs beyond static models.

## Abstract (web summary)

*Helicobacter pylori* Cag type IV secretion system is a nanomachine that delivers virulence factors into host cells. Here, authors reconstitute its assembly intermediates and define the stepwise initiation of the central cylinder, showing a precise coordination involved in its formation.

## Introduction

Persistent *Helicobacter pylori* infection increases risk of gastric cancer progression, one of the global causes of death^1,2^. Cytotoxin-associated gene pathogenicity island (*cag*PAI) is a major virulence factor contributing to the pathogenesis of *H. pylori*. It is a 40 kb chromosomal insertion element encoding around 30 Cag proteins that constitute the Cag type IV secretion system (CagT4SS)^2–4^. CagT4SS is a versatile nanomachine that translocates oncoprotein CagA, lipopolysaccharide metabolites, peptidoglycans and DNA into host cells upon contact^5–8^. Apart from the VirB1-11 and VirD4 homologues that are found in evolutionarily conserved T4SSs, CagT4SS also carries additional species-specific proteins, and is categorized as an expanded T4SS system^9,10^.

**Fig. 1:**
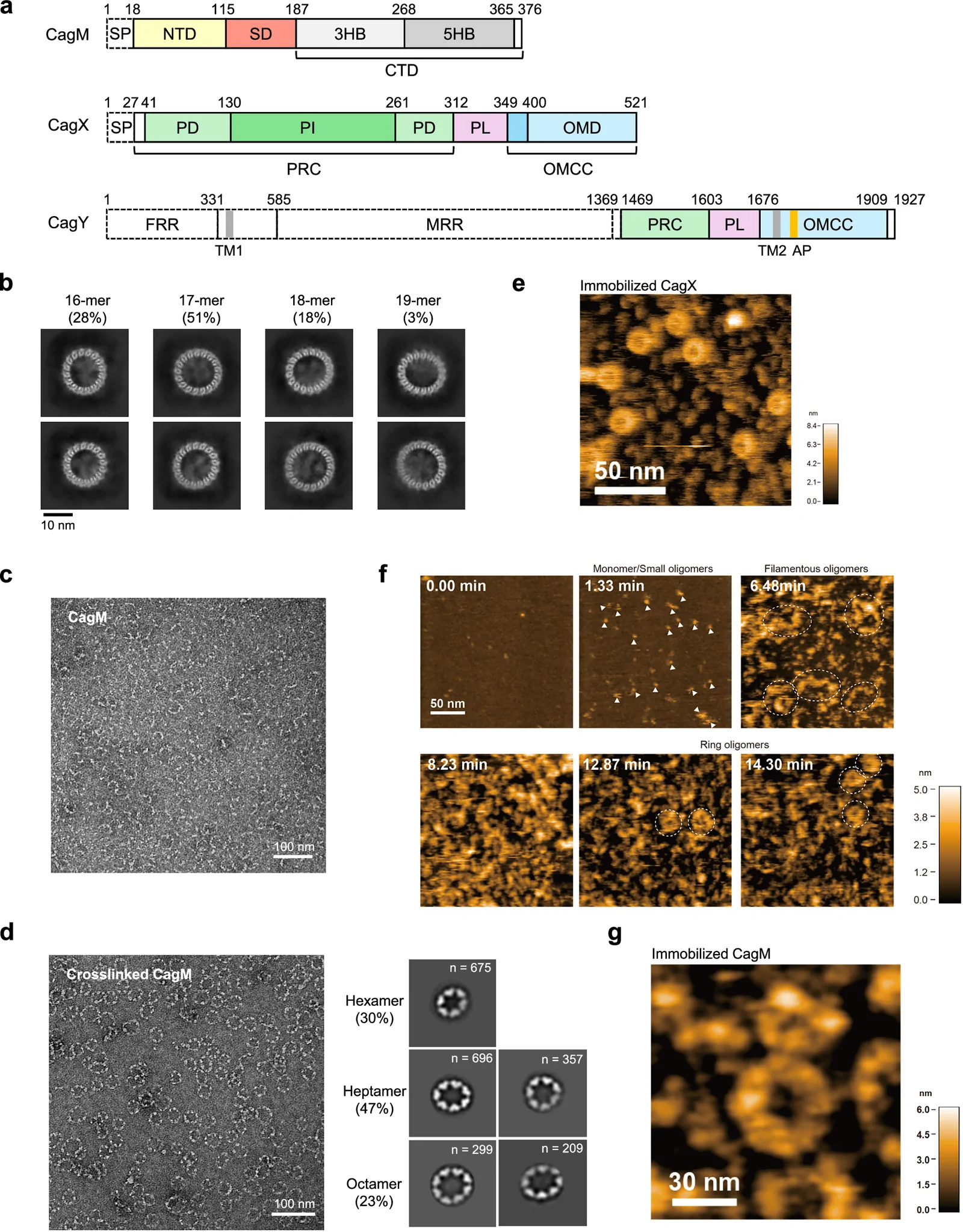
Homo-oligomerization properties of CagX and CagM. **a** Domain organization of CagM, CagX and CagY. Regions boxed in solid line, including full-length CagM and CagX (without signal peptide), and PRC-OMCC of CagY, were used in this study. SP: signal peptide; NTD: N-terminal domain; SD: small domain; 3HB: 3-helix bundle; 5HB: 5-helix bundle; CTD: C-terminal domain; PD: periplasmic domain; PI: periplasmic insertion; PRC: periplasmic ring complex; PL: periplasmic linker; OMD: outer membrane domain; OMCC: outer membrane core complex; FRR: 5’-repetitive region; TM: transmembrane region; MRR: middle repeat region; AP: antenna projections. **b** Representative cryo-EM 2D classes of CagX revealing ring structures with oligomerization states ranging from 16- to 19-mer. **c**, **d** Representative negative stained TEM images of CagM (**c**) without and (**d**) with DSSO crosslinkers. The experiment was repeated three times independently with similar results. 2D class averages of crosslinked CagM shows ring formation with different oligomeric states. **e** Representative HS-AFM image of immobilized CagX showing ring structures. **f** Time-lapsed HS-AFM images of CagM added to the observation buffer (upper right; continuous mode, 1 s/frame) on 0.1% APTES/mica showing filamentous CagM. **g** HS-AFM image of immobilized CagM showing ring structures similar to TEM images. The experiment was repeated three times independently with similar results.

The molecular architecture of CagT4SS consists of pilus, outer membrane core complex (OMCC), periplasmic ring complex (PRC), stalk, collar and inner membrane complex^11–13^. Current assembly models propose that CagT4SS is built from the outer membrane, where CagX, CagY and CagM serve as initiators and form the central cylinder of PRC-OMCC. This is followed by the incorporation of peripheral components, CagT and Cag3 via interactions with CagM^12,14,15^. CagX (VirB9), CagY (VirB10) and CagT (VirB7) are VirB homologs while CagM and Cag3 are *H. pylori*-specific^16^. All five Cag proteins are indispensable in CagT4SS formation and CagA translocation, underscoring their structural and functional importance^17–20^. The recent cryo-EM models of OMCC and PRC have revealed the overall arrangement and atomic details of these core proteins^18^. Specifically, OMCC is a 14-fold symmetric mushroom-like structure and each asymmetric unit contains CagX, CagY, CagM, CagT and Cag3 in a stoichiometric ratio of 1:1:2:2:5. The periphery of OMCC is built by stacking of CagT and Cag3 through their conserved N0 domain, which their conformational dynamics is further highlighted by a recent study^15^. For the two CagM copies at the base of OMCC, CagM-1 lies below Cag3 at the periphery and CagM-2 is adjacent to CagX in the central core. The available CagM structural model, however, contains only the C-terminal domain (CTD) (Fig. 1a). The N-terminal region (~180 amino acid residues long), which is essential for self-homodimerization remains unresolved^18,21^. Both CagX and CagY possess elongated conformations and concurrently form the 17-fold symmetric PRC structure in a 1:1 molar ratio. The OMCC region of CagX consists of an α-helix and a globular outer membrane domain (OMD) (Fig. 1a). For CagY, apart from the globular fold that interacts with CagX, its OMCC region also contains an unresolved antenna projection, which is required for CagT4SS activity^22^. While the domain organization of CagX and CagY are well defined, the 14:17 symmetry mismatch between OMCC and PRC is not understood. Noteworthy, CagY is ~200 kDa larger than any other VirB10 homologs due to the presence of two additional repeat segments. It also spans across the inner and outer membrane via two transmembrane regions, underlining the variability of CagT4SS from other T4SSs^23^.

Until now, the presence of PRC substructure is exclusive to expanded T4SSs found in *H. pylori* and *L. pneumophila*^10,18,24^, however, the two systems are structurally diverse as revealed by recent mutagenesis and cryo-ET. For example, PRC in *L. pneumophila* includes additional species-specific components beyond VirB9 and VirB10 homologues, and its formation is dependent on the OMCC^25^. Conversely, CagT4SSs isolated from *cagM* and *cagT* knockout strains retained an intact PRC but lacked OMCC^26^, implying that PRC has greater structural stability and probably is a pivotal substructure for OMCC formation. How CagX, CagY, and CagM are coupled to initiate CagT4SS assembly is fully unclear. The major bottleneck in advancing our understanding of this supramolecular architecture is the isolation of different structural intermediates from *H. pylori* mutant strains for characterization. In this study, we integrate multiple biochemical and biophysical techniques to elucidate the structural determinants for the CagT4SS initiation pathway. We show the PRC domain of CagX is a scaffold for the incorporation of CagY and CagM. By using single-particle cryo-electron microscopy (cryo-EM), hydrogen-deuterium exchange mass spectrometry (HDX-MS), and high-speed atomic force microscopy (HS-AFM) single-molecule imaging, we further present the structural dynamics of their in vitro reconstituted subassemblies and the significance of PRC in CagT4SS assembly. This approach not only allows us to overcome the challenges in deducing the molecular events of CagT4SS assembly but also open avenues for the studies of other intricate biological complexes.

## Results

### Self-oligomerization of CagX may act as a scaffold for early CagT4SS assembly

Among all Cag proteins, CagX, CagY and CagM are central to the assembly initiation process^12,14,18,26^. We considered that one of them would first form a scaffold, which the other two Cag components are then loaded to build the PRC-OMCC substructures. Here, we expressed and purified recombinant full-length CagX and CagM (without signal peptide), and CagY_PRC-OMCC_, where the subscripts denote the domain coverage using *E. coli* expression system. Their self-oligomerization properties by size exclusion chromatography coupled with static light scattering (SEC-SLS) and negative stain TEM were analyzed. SEC-SLS showed that the majority of CagX has an average experimental mass of ~57 kDa, indicating a monomeric form (Suppl. Fig. 1a). CagX was also dynamically exchanged to form a dimer as shown by the shoulder peak with ~105 kDa. Interestingly, under TEM, we observed CagX self-oligomerized into a ring structure (Suppl. Fig. 1b). Further characterization by cryo-EM two-dimensional (2D) class averages revealed symmetries ranging from 16- to 19-fold with the 17-mer species being the most dominant (Fig. 1b and Suppl. Fig. 1c). Compared with the CagX ring structure within the previously reported PRC model^18^, these in vitro self-assembled CagX rings share similar diameter (~15 nm) and symmetry. By examining two truncated constructs CagX_PRC_ and CagX_PL-OMCC_ (Fig. 1a), we confirmed the CagX ring complex was formed only by the PRC region, not the PL-OMCC (Suppl. Fig. 1d, e). For CagY, another key constituent of PRC and OMCC, the three constructs CagY_PRC-OMCC_, CagY_PRC_, and CagY_OMCC_ we generated all presented as monomers in SEC-SLS (Suppl. Fig. 1f) and no ring particles were observed under TEM (data not shown), indicating their weak intermolecular interactions. On the other hand, CagM, the peripheral component at the OMCC base was eluted in a broad peak with an average mass of ~488 kDa, suggesting the existence of transient structures in different oligomeric states (Suppl. Fig. 1g). In line with SEC-SLS analysis, heterogeneous crescent-shaped particles were detected under TEM (Fig. 1c). Intriguingly, when added with disuccinimidyl sulfoxide (DSSO) crosslinker prior to TEM imaging, CagM formed ring structures with diameter ~33 nm (Fig. 1d), similar to that of the OMCC structure reported previously^18^. Further TEM 2D class averages revealed the 6 to 8-mer rings are composed of reuleaux-like triangles pointing toward the centre.

To further elucidate the dynamic behaviour of the three Cag proteins at single-molecule level, we applied HS-AFM. At a low concentration (0.05 µM), CagX monomer exhibited as two globular domains connected by a linker (Suppl. Fig. 2a, Suppl. Movie 1). With reference to the CagX structural model (PDB ID: 6X6S and 6X6J)^18^, the small and large domains likely correspond to the globular outer membrane domain (OMD) and PRC (Fig. 1a). In agreement with the findings of negative stain TEM (Suppl. Fig. 1b), when CagX was immobilized onto APTES-treated mica and at a higher concentration (3.33 µM), self-oligomerization into ring structures was observed (Fig. 1e). On the other hand, at a concentration of 0.05 µM, CagY_PRC-OMCC_ presented as a two-domain structure linked by a flexible loop (Suppl. Fig. 2b, Suppl. Movie 2). Previous studies reported that the PRC subcomplex of CagT4SS is structurally more stable^26^, therefore the larger globular structure likely correspond to the PRC while the smaller domain contains the OMCC regions of CagY (Fig. 1a). For both CagX and CagY, the linker connecting the PRC and OMD/OMCC had a widespread distribution (8.8 ± 0.1 nm in CagX, 15.0 ± 0.1 nm in CagY; Gaussian fitting error) in end-to-end distance, suggesting the dynamic nature of the two proteins would be contributed by the periplasmic linker (PL) (Suppl. Fig. 2c). For CagM at a low concentration (0.05 µM), a gourd-shaped molecule with an extended flexible tail was observed (Suppl. Fig. 2d, Suppl. Movie 3). Likely, the core body corresponds to the CTD, while the tail corresponds to the flexible, unresolved N-terminus. The average contour length of the tail was measured as 24.4 ± 4.6 nm (s.d.), which is approximately one-third of the fully extended length expected for the ~180-residue N-terminal region (~68 nm), suggesting the presence of partial secondary structures. Upon addition of CagM at a higher concentration (0.6 µM) onto APTES-treated mica, CagM initially appeared as monomers or small oligomers (arrows), which subsequently assembled into filamentous structures (dashed ellipses) similar to those observed under TEM (Fig. 1c), and eventually into ring oligomers (dashed circles) (Fig. 1f, Suppl. Movie 4). The CagM filamentous structures underwent continuous dissociation and re-association, resulting in the formation of different oligomeric species. At an even higher concentration (10 µM), stable ring structures were readily observed (Fig. 1g), indicating that surface immobilization at high concentration reduced the dynamicity of CagM, similar to the crosslinked species (Fig. 1d). These results collectively suggested that while both CagX and CagM possess self-oligomerization properties, the spontaneous formation of the more stable CagX ring functions as a docking platform triggering the CagT4SS synthesis.

### In vitro reconstitution of CagM-CagX complex revealing a double-ring complex

**Fig. 2:**
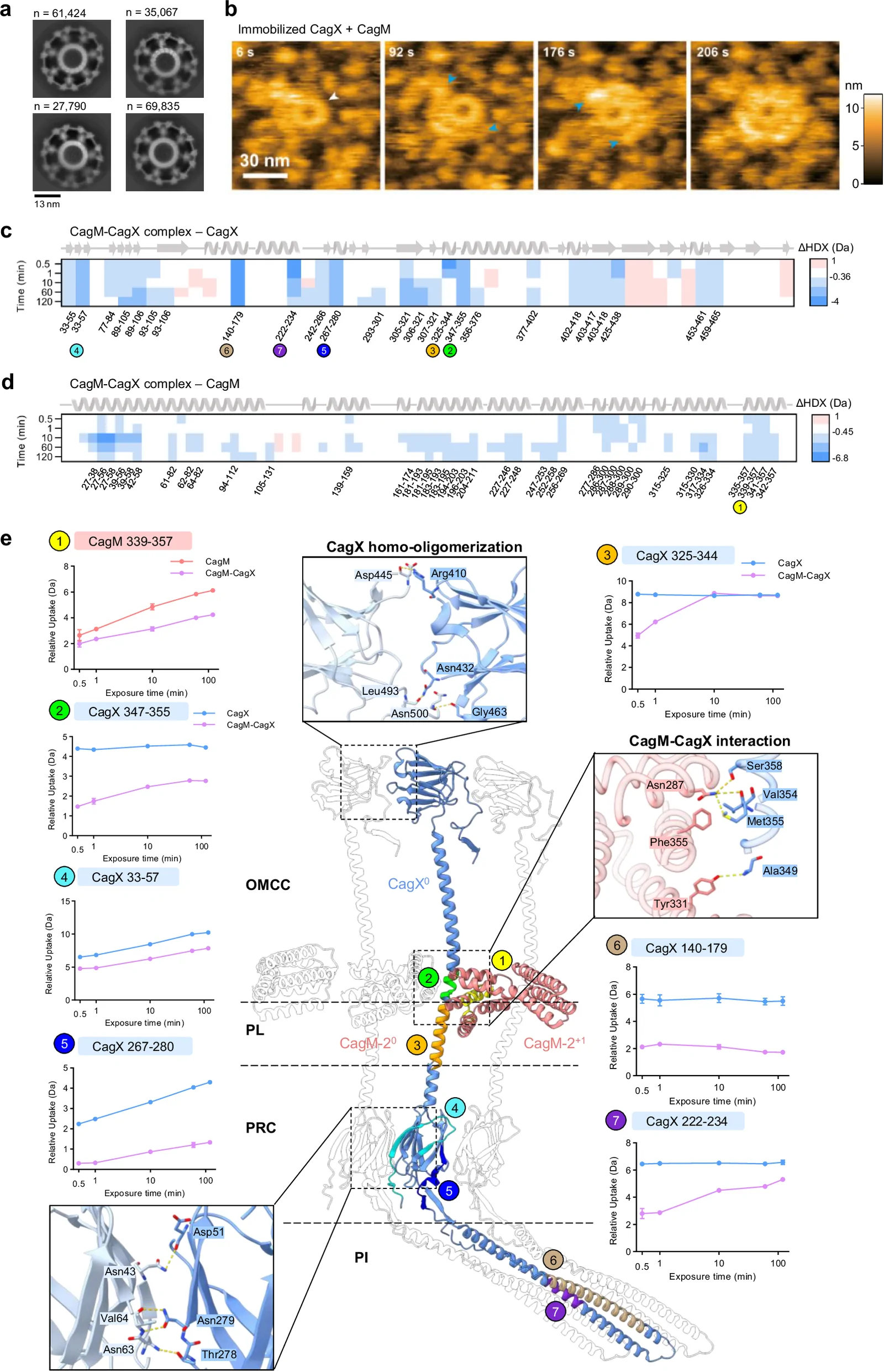
Structural dynamics involved in CagM-CagX complex formation. **a** Representative cryo-EM 2D classes of CagM-CagX complex showing a double-ring structure. **b** Time-lapsed HS-AFM images of adding CagM into CagX pre-immobilized on APTES-treated mica (intermittent scanning mode with 1 frame on and 3 frames off at an imaging speed of 0.5 s/frame) revealing the formation of CagM-CagX double ring complex. White arrow indicates CagX ring. Blue arrows indicate open ends of CagM binding to CagX ring, which change along the time until a complete double ring is formed. **c**, **d** Chiclet plots displaying the differential HDX (ΔHDX: complex state minus apo state) of (**c**) CagX and (**d**) CagM across all time points. Significant ΔHDX was defined using 95% confidence interval (shaded in red or blue). Secondary structure elements corresponding to the peptides are indicated above the plot. **e** CagM-2-CagX complex showing three ASUs is modelled by previous cryo-EM model (PDB ID: 6X6S and 6X6J) and AlphaFold3 prediction (PI and PL region of CagX) without considering symmetry mismatch, which CagM-2 is colored in light coral and CagX is colored in cornflower blue. Kinetic plots of colored regions, accounting for significant ΔHDX are shown. Error bars indicate standard deviations for each time point (*n* = 3) and the center of each error bar is defined as the mean. Molecular interactions involved in CagX homo-oligomerization and CagM-CagX interaction are highlighted in respective zoom-in boxes.

**Fig. 3:**
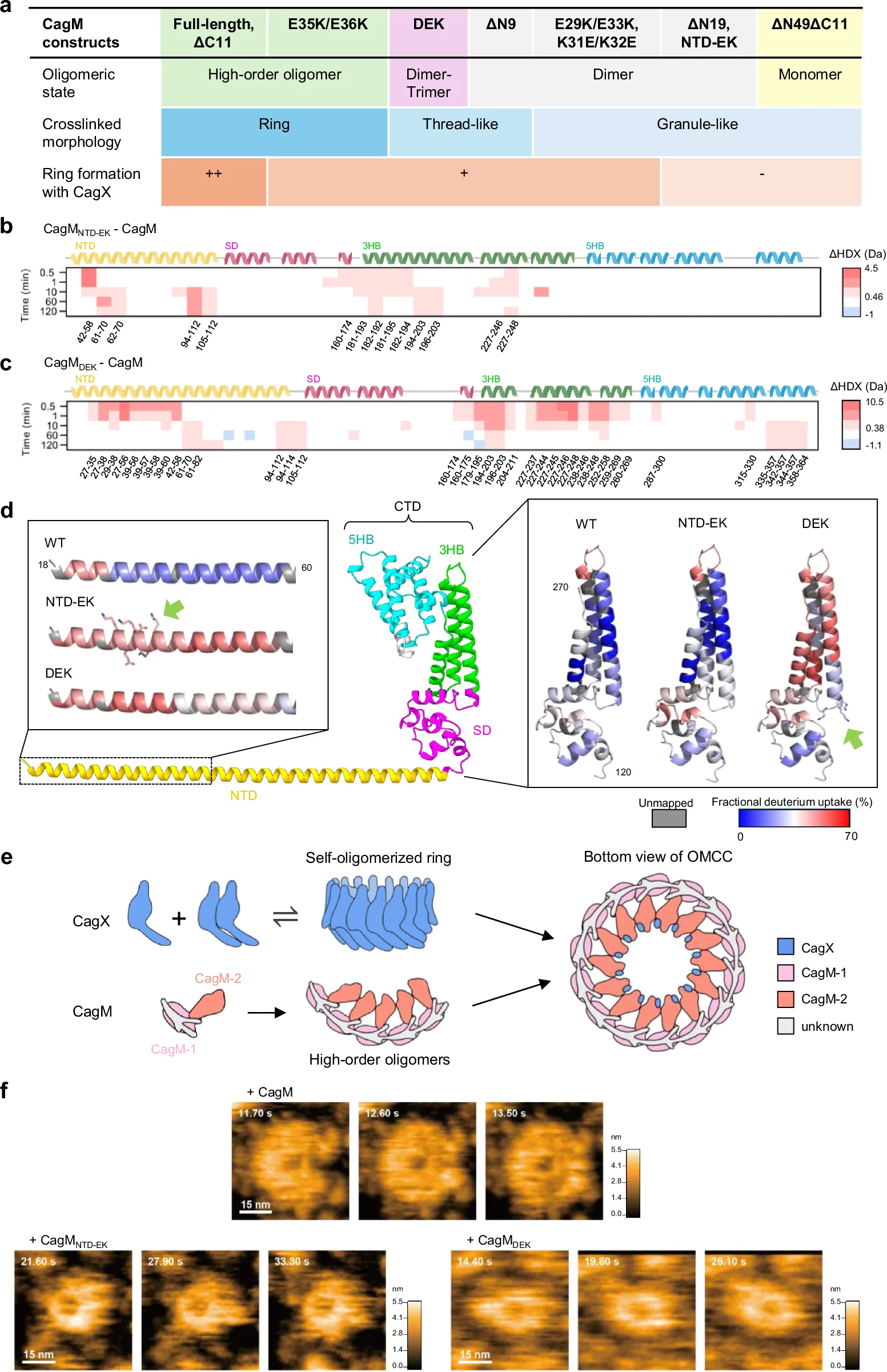
Characterization of CagM oligomerization and ring formation. **a** A summary table displaying the crosslinked morphologies and ring formation capability with CagX in different CagM mutants, categorized by the oligomeric states of CagM. ++: intact double ring; +: double ring with less integrity; -: no double ring formation. **b**, **c** Chiclet plots displaying the differential HDX (ΔHDX) of (**b**) CagM_NTD-EK_ - CagM and (**c**) CagM_DEK_ - CagM across all time points. Significant ΔHDX was defined using 95% confidence interval (shaded in red or blue). Secondary structure elements and domain organization corresponding to the peptides are indicated above the plot. **d** Predicted CagM structure using AlphaFold3 with mutated sites shown in sticks and indicated by arrows. The structure is colored based on its structural domains: NTD (yellow), SD (magenta), 3HB (lime) and 5HB (cyan). Deuterium uptake differences observed in residues 18–60 and 120–270 of wild-type CagM, CagM_NTD-EK_ and CagM_DEK_ using HDX-MS at 0.5 min time interval are mapped onto the structure, suggesting these regions are responsible for oligomerization and ring formation. **e** Schematic diagram of CagM-CagX double-ring formation. Monomeric and dimeric CagX (cornflower blue) self-oligomerize into a ring structure while dimeric CagM (CagM-1 in light pink and CagM-2 in light coral) oligomerizes into high-order oligomers. Upon interaction, double-ring complex possibly resembling the previously resolved cryo-EM OMCC model (PDB ID: 6X6S) is formed, showing both CagM copies and the unknown densities in light grey located at the circumference. **f** Time-lapse HS-AFM images of adding CagM (upper), CagM_NTD-EK_ (lower left), and CagM_DEK_ (lower right) to pre-immobilized CagX on APTES-treated mica, revealing their outer ring formation tendency in the presence of CagX. These images were acquired with the intermittent scanning mode with 1 frame on and 5 frames off at an imaging speed of 0.15 s/frame.

The current out-to-in CagT4SS assembly model proposes that CagM plays a crucial role in completing the central cylinder for the elaboration of OMCC and the inner membrane complex^12,14,26^. How this species-specific Cag protein interacts with CagX and helps define the periphery of OMCC has not been elucidated. Given the highly dynamic nature of CagM as shown by HS-AFM, deducing the coupling of CagM based on the OMCC cryo-EM model, which already consists of Cag3 and CagT, has limitations, not to mention that the structure of the N-terminal region of CagM is largely unknown. Here, we reconstituted CagM-CagX complex by co-incubating individually purified proteins in a molar ratio of 2:1 followed by SEC (Supple. Fig. 3a). Comparison with the elution profiles of individual proteins and the band intensities of CagM and CagX in the co-eluted fractions indicated the two proteins formed a complex with a stoichiometric ratio of 2:1. The sample was subjected to cryo-EM analysis (Suppl. Fig. 3b) and several rounds of reference-free 2D classification. When compared with the self-assembled CagX ring structure (Fig. 1b), a double-ring structure with outer ring diameter of ~33 nm was identified (Fig. 2a). Although not all asymmetric units in the inner ring were clearly shown, most particles had a 17-fold symmetry, resembling CagX PRC. The outer ring is composed of 14 globular structures connected by continuous density at the circumference, sharing similar structural features as the outer ring of the crosslinked CagM under TEM (Fig. 1d). By applying a CagM-focused mask and C7 symmetry, a 7.77 Å cryo-EM map was reconstructed (Suppl. Fig. 3). The two rings are bridged by seven pairs of intertwining spokes, instead of 14 spokes as in the previous cryo-EM model^18^. The underlying mechanism of this arrangement is unclear. Yet, we reasoned that the reconstituted CagM-CagX complex consisting of CagM and CagX in 2:1 stoichiometric ratio represents an intermediate state prior to the association of Cag3 and CagT.

We next conducted HS-AFM to monitor the CagM-CagX assembly in real time. CagX was first immobilized on APTES-treated mica, followed by the addition of CagM in the observation buffer. Wide-field imaging in continuous scanning mode revealed that CagM molecules progressively accumulated around the CagX rings, repeatedly associating with and dissociating from the ring periphery (Suppl. Fig. 4, Suppl. Movie 5). However, continuous scanning perturbed the loosely bound CagM through the mechanical force of the AFM tip, making it difficult to capture the complete formation of a double-ring complex under this imaging mode. To circumvent this, we employed intermittent scanning mode, in which imaging was performed every few frames to minimize tip-induced perturbation. Under this condition, CagM was observed to undergo repeated cycles of binding and dissociation, yet ultimately assembled into a stable double-ring complex (Fig. 2b, Suppl. Movie 6). Quantitative analysis of CagM binding kinetics will be discussed in the last result section.

### Molecular dynamics of CagM-CagX complex formation

To further unveil the binding interface and molecular dynamics involved in CagM-CagX complex formation, HDX-MS was applied (Suppl. Figs. 5, 6, Suppl. Table 1 and Suppl. Data 1, 2). By comparing the deuterium uptake of coincident peptides between apo- and complex states that covered 81.4% of CagX and 93.2% of CagM sequence, differential HDX chiclet plots were generated (Fig. 2c, d). Based on the partially resolved cryo-EM model^18^, CagM-CagX interaction lies on the cleft formed by the 3HB and 5HB of CagM-2 and the tip of the long helix (residue 349–393) upstream of the OMD of CagX in the neighbouring asymmetric unit (ASU) (Suppl. Fig. 7a). CagM-1 located at the periphery of OMCC is too distant and unlikely to form any interactions with CagX. Therefore, we combined AlphaFold3 prediction for the unresolved structure in CagX, namely PL and periplasmic insertion (PI), and created a CagM-2-CagX model (Fig. 2e and Suppl. Fig. 8a)^18,27^. We identified multiple regions with reduced HDX that map well to the CagM-CagX binding interface on the cryo-EM model. These include the CTD of CagM, especially residues 339-357, and the long helix (residues 347-376) within the OMCC of CagX (Fig. 2c–e). The importance of this helical region was also demonstrated by the pull-down assays, which revealed a direct interaction of CagM with CagX_OMCC_ but not CagX_OMD_ (Suppl. Fig. 7b). On this basis, we constructed additional CagX truncations to define the CagM binding sites. CagM interacted with CagX_27-363_ but not CagX_27-353_ (Suppl. Fig. 7b), suggesting residues 354–363 of CagX are critical. On the other hand, transient reduction in deuterium uptake was observed in CagX residues 325-344 corresponding to PL. It is possible that this region may undergo conformational changes temporarily to accommodate CagM and/or be related to PRC:OMCC symmetry mismatch (Fig. 2c, e and Suppl. Fig. 7a). Additionally, reduction of deuterium uptake in regions related to CagX homo-oligomerization were identified. Consistent with the cryo-EM model, these include the β-sandwich of PRC region (residues 33–57 and 267–280) and OMD (residues 402–418 and 453–465) in CagX (Fig. 2c, e), indicating CagM binding contributes to the stability of CagX self-oligomerization via enhanced hydrogen bond protection. A notable decrease in uptake in the PI region (residues 140–179 and 222–234) was also detected (Fig. 2c, e). It is plausible that the predicted helical hairpins of CagX are stacked with each other in the ring structure. On the other hand, reduced HDX was observed in two unresolved regions of CagM, residues 27–58 in the NTD and residues 160–203 spanning from upstream region of CTD to 3HB (Fig. 2d). Consistent with a previous study showing that the N-terminus of CagM is involved in homo-oligomerization^21^, our HDX-MS data revealed that an additional region participates in the CagM oligomerization, likely for CagM-1 and CagM-2 interactions.

### CagM ring formation requires multimerization of CagM dimers via charged-charged interactions

To delineate the molecular determinants for the self-assembly of CagM, different CagM mutants were constructed and their ability in ring formation was examined under TEM after DSSO crosslinking. We first affirmed that the unresolved 11 residues at the C-terminus in previous cryo-EM model^18^ are not involved in ring formation, as mutant CagM_ΔC11_ behaved similarly as full-length CagM (Fig. 3a and Suppl. Fig. 9a). Deletion of N-terminal residues 18–26 (CagM_ΔN9_) and residues 18–36 (CagM_ΔN19_) resulted in filamentous and granular particles, respectively, suggesting the N-terminus is required for ring formation while residues 27–36 are critical for oligomerization (Fig. 3a and Suppl. Fig. 9a). All six charged residues within this region (residues 29-36) were mutated to its opposite charge, generating mutant CagM_E29K/K31E/K32E/E33K/E35K/E36K_ (namely CagM_NTD-EK_) defective in ring formation. By using mutants CagM_E29K/E33K_, CagM_K31E/K32E_ and CagM_E35K/E36K_, we further defined the hotspots for CagM ring formation to residues Glu29, Lys31, Lys32 and Glu33 (Fig. 3a and Suppl. Fig. 9a). As indicated by the HDX-MS results, another charged region (residues 183-185) showing lower deuterium uptake was possibly responsible for CagM oligomerization (Fig. 2d). These residues upstream of CTD of CagM-2 may belong to the unassigned polyAla densities lying closely to CagM-1 in the cryo-EM model^18^ (Suppl. Fig. 9b). Therefore, CagM_D183E/E184K/E185K_ (namely CagM_DEK_) was constructed, and this mutant was defective in ring formation (Fig. 3a and Suppl. Fig. 10a). We further studied the relationship of oligomeric states and ring formation. Ring-forming mutants CagM_ΔC11_ and CagM_E__35K/E36K_ displayed as filamentous particles in the absence of crosslinkers (data not shown) and existed as higher-order oligomers (decamer or above) (Fig. 3a and Suppl. Fig. 10a, b). The dimeric and decameric forms of CagM_E35K/E36K_ observed by SEC-SLS and analytical ultracentrifugation analysis, respectively, suggested that mutation at residues 35–36 may attenuate CagM oligomerization, rendering it more susceptible to dissociation by the shearing force during SEC-SLS. Conversely, ring-defective constructs mainly existed in dimeric states (Fig. 3a and Suppl. Fig. 10a), potentially acting as the minimal building blocks as proposed previously^21^. Taken together, multimerization of CagM dimers to form a complete ring structure involves charged-charged interactions at the NTD and upstream of CTD.

We further applied HDX-MS to demonstrate the dynamic changes related to these two regions in CagM oligomerization. Mutants CagM_NTD-EK_ and CagM_DEK_ defective in ring formation but in different oligomeric forms were studied in parallel with wild-type CagM. Around 90% peptide coverage was obtained individually while peptides coincidental with CagM only yielded 84.1% and 92.6% coverage in CagM_NTD-EK_ and CagM_DEK_ respectively (Suppl. Figs. 11, 12, Suppl. Table 1 and Suppl. Data 3, 4). The lower coverage of overlapping peptides between wild-type and mutated CagM may be due to their differences in acid-denatured states caused by different oligomeric states, resulting in distinctive pepsin digestion patterns. When comparing the limited overlapped peptides between wild-type and mutants, significant HDX increase was mainly noticed in residues 18–60 and 120–270, likely resulting from the differences in oligomeric states (Fig. 3b, c). Therefore, we mapped their respective fractional deuterium uptake at the shortest time interval (0.5 min) in these two regions onto the CagM model predicted by AlphaFold3 (Fig. 3d and Suppl. Fig. 8b)^27^. The previously unresolved ~180 residues at the N-terminal region are predicted as a long α-helix followed by a small globular domain (SD). Compared with the wild-type, both mutants demonstrated significantly increased deuterium uptake in the long α-helix at NTD and slightly elevated uptake in the SD (Fig. 3d), implying greater solvent exposure and weakened hydrogen bond strength in these regions due to impaired high-order oligomerization. These findings align with the HDX-MS results on CagM-CagX complex (Fig. 2d, e). Another striking difference was solely observed in mutant CagM_DEK_ whose 3HB in CTD displayed a higher deuterium incorporation than wild-type (Fig. 3c, d). It is suspected that the mutation sites may disrupt the stability of the helical tip of 3HB, resulting in less ordered CTD.

### Distinct interfaces in CagM homo-oligomerization and CagM-CagX hetero-oligomerization

Next, we performed pull-down assays to assess the interaction of these CagM mutants with CagX. Intriguingly, all CagM mutants retained their interaction with CagX, demonstrating that CagM homo-oligomerization and hetero-oligomerization with CagX involve different interfaces (Suppl. Fig. 13a). To verify this, CagM truncations containing NTD-SD or CTD required for CagM homo-oligomerization and CagM-CagX hetero-oligomerization, respectively, were created. Unexpectedly, no CagM-CagX interaction was detected (Suppl. Fig. 13b). Instead, the minimum region for CagX interaction was mapped to residues 67–365 (CagM_ΔN49ΔC11_). These further implied that the CagM-CagX binding interface may be more extensive than revealed from the cryo-EM model^18^, encompassing part of the NTD and SD and contributing to complex formation and stability. Furthermore, CagM_ΔN49ΔC11_ was unable to form CagM ring after crosslinking and presented as a monomer in SEC-SLS analysis, indicating that CagM dimerization interface is distinct from high-order oligomerization (Fig. 3a and Suppl. Fig. 14).

Collectively, the assembly mechanism of CagM-CagX complex would require (i) self-oligomerization of CagX ring, (ii) high-order oligomerization of CagM and (iii) association of CagM onto CagX (Fig. 3e). To explore the interplay between the self-assembly of CagM and its incorporation into CagX, all CagM mutants and CagX were co-incubated and purified by SEC prior to negative stain TEM. Intact CagM-CagX double-ring particles were only observed when high-order oligomeric CagM (wild-type and CagM_ΔC11_) was added, highlighting NTD and SD are necessary for the formation of the outer ring in CagM-CagX complex (Fig. 3a and Suppl. Fig. 15). Two mutants, CagM_NTD-EK_ and CagM_DEK_ representing the fully abolished and weakened homo-oligomerization, respectively, were further subjected to HS-AFM studies. As expected, no intact outer ring was formed after adding CagM_NTD-EK_ or CagM_DEK_ to immobilized CagX. Yet, the coupling of CagM-2 onto the CagX ring was observed (Fig. 3f, Suppl. Movies 7–9), in line with our biochemical studies.

### Divergent dynamics of CagX-CagY complex featuring a stable PRC and highly flexible OMCC

**Fig. 4:**
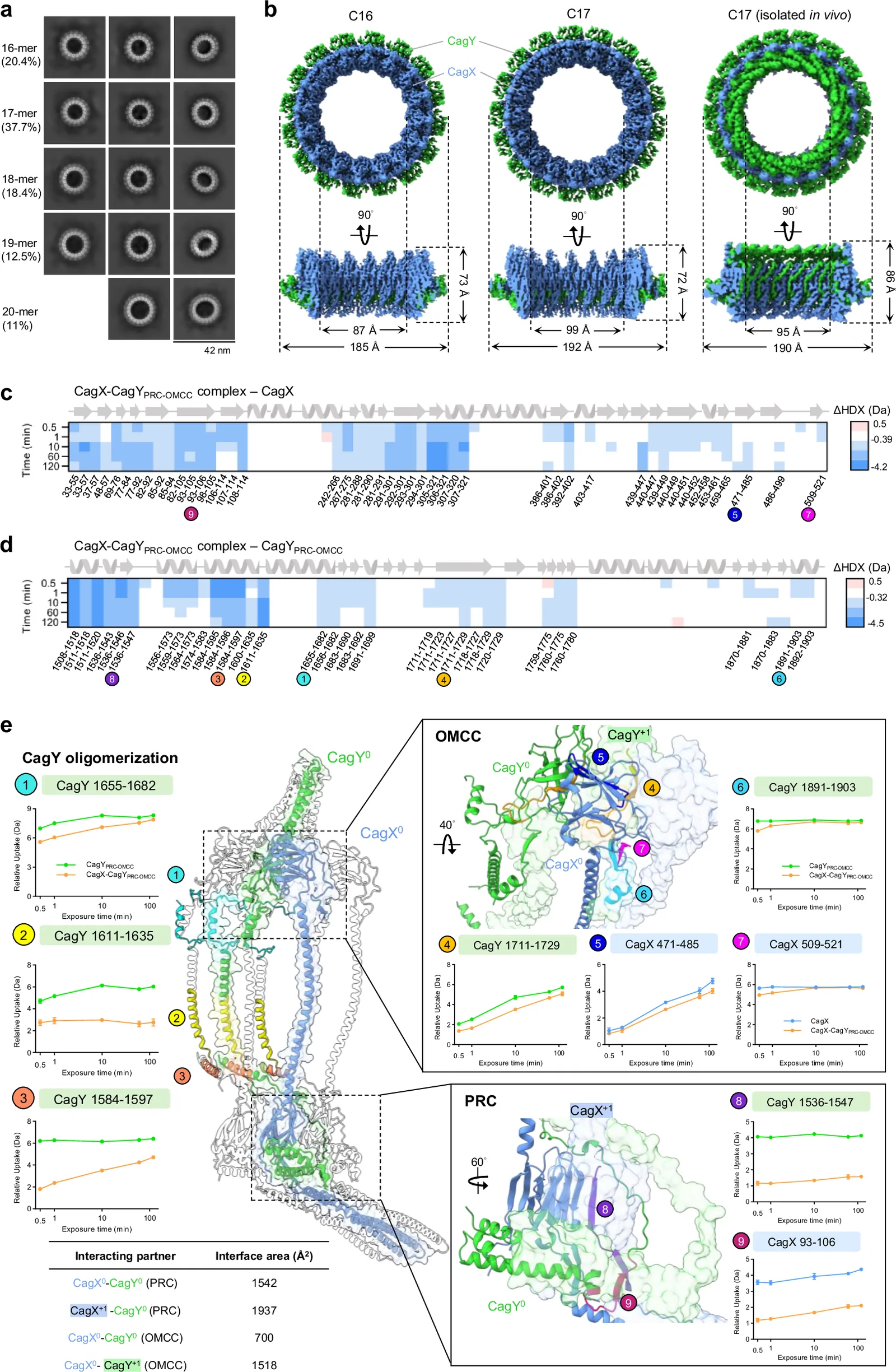
Architecture of CagX-CagY_PRC-OMCC_ complex and its dynamics analyzed by HDX-MS. **a** Representative cryo-EM 2D classes of CagX-CagY_PRC-OMCC_ complex showing different oligomeric states in various orientations. **b** Comparison of cryo-EM maps between our in vitro reconstituted CagX-CagY_PRC-OMCC_ complex applied with C16 and C17 symmetries and PRC isolated in vivo previously (EMD-20021), which CagX is colored in cornflower blue and CagY is colored in lime. **c**, **d** Chiclet plots displaying the differential HDX (ΔHDX: complex state minus apo state) of (**c**) CagX and (**d**) CagY_PRC-OMCC_ across all time points. Significant ΔHDX was defined using 95% confidence interval (shaded in red or blue). Secondary structure elements corresponding to the peptides are indicated above the plot. **e** CagX-CagY_PRC-OMCC_ complex showing three ASUs is modelled by previous cryo-EM model (PDB ID: 6X6S and 6X6J) and AlphaFold3 prediction (PI region of CagX and PL region of both CagX and CagY) without considering symmetry mismatch, which CagX is colored in cornflower blue and CagY is colored in lime. Kinetic plots of colored regions responsible for CagY oligomerization and CagX-CagY interaction, accounting for significant ΔHDX are shown. Error bars indicate standard deviations for each time point (*n* = 3), and the centre of each error bar is defined as the mean. A table displaying CagX-CagY_PRC-OMCC_ interface area in PRC or OMCC region within and across ASUs resolved in previous cryo-EM model.

Since CagX_PRC_ but not CagX_PL-OMCC_ was critical for CagX ring formation, we suspected the proposed CagX scaffold would also be sufficient to facilitate the incorporation of CagY, leading to the formation of PRC or the central cylinder of PRC-OMCC. To confirm this, CagX-CagY_PRC-OMCC_ complex was first reconstituted in vitro by co-incubating individually purified proteins in a molar ratio of 1:1 followed by SEC (Suppl. Fig. 16) and subjected to cryo-EM single-particle analysis. Cryo-EM 2D classification revealed multiple oligomeric forms, ranging from 16- to 20-mer (Fig. 4a). Ab initio reconstructions were generated from selected particles in C16 and C17 classes and yielded structural models with an overall resolution of 2.87 Å and 2.58 Å, respectively (Suppl. Figs. 17, 18 and Suppl. Table 2). Clearly, the maps only show the PRC substructure but not PL and OMCC (Fig. 4b). The dimensions of the C17 model are analogous to the previously reported PRC structure^18^. In both of our models, CagX (residues 32–124, 261–320) and CagY (residues 1469–1544) form the inner and the outer rings, respectively (Suppl. Fig. 18b). Alignment of the respective protomers in all models revealed a Cα RMSD of around 1 Å, suggesting the in vitro reconstituted CagX-CagY complex is comparable to the in vivo isolated model.

The missing densities of PL and OMCC in the cryo-EM maps imply that they are highly flexible structures in the co-purified CagX-CagY_PRC-OMCC_ complex. To further evaluate the interaction between CagX and CagY, GST-tagged CagY_PRC-OMCC_, CagY_PRC_, and CagY_OMCC_ were used as baits in pull-down assays with CagX and its various truncations (CagX_PRC_, CagX_PL-OMCC_, CagX_OMCC_, and CagX_OMD_). While GST-CagY_PRC-OMCC_ interacted with all CagX constructs, GST-CagY_PRC_ interacted only with CagX and CagX_PRC_ (Suppl. Fig. 19a). Though interaction between GST-CagY_OMCC_ and CagX was detected, the band intensity of CagX was much weaker than that pulled by GST-CagY_PRC-OMCC_ and GST-CagY_PRC_. These results indicated that the presence of the PRC domain in both CagX and CagY are crucial. We next performed cryo-EM analysis on purified CagX-CagY_PRC_ complex (Suppl. Fig. 19b). 2D class averages revealed ring architectures comparable to CagX-CagY_PRC-OMCC_, suggesting that the OMCC region had no effect on the assembly of PRC substructure (Suppl. Fig. 19c). Furthermore, the dissociation constants of CagX-CagY_PRC-OMCC_ (0.12 μM) and CagX-CagY_PRC_ (0.93 μM) were at least 3-fold lower than that of CagX-CagY_OMCC_ (3.44 μM) (Suppl. Fig. 19d). These findings illustrated that the primary binding interface of the CagX-CagY complex is located on the PRC domain.

We further performed HDX-MS to investigate the molecular dynamics involved in CagX and CagY_PRC-OMCC_ interaction (Suppl. Figs. 20, 21, Suppl. Table 1 and Suppl. Data 5, 6). Reduction in relative deuterium uptake in both CagX and CagY were observed in multiple regions (Fig. 4c, d), which lie on the CagX-CagY interfaces in PRC and OMCC as in the cryo-EM model (Fig. 4e)^18^. Specifically, persistent HDX decline in CagY (residues 1536–1547) and CagX (residues 93–106) highlighted CagX-CagY interaction across ASUs (CagX^+1^-CagY^0^), which has an equivalent interface area to CagX^0^-CagY^0^ (1937 Å^2^ vs 1542 Å^2^) (Fig. 4e inlet table). OMCC region (residues 1711–1729) of CagY showed a lower exchange rate since it becomes less exposed upon interaction with CagX in the same and neighbouring ASUs (Fig. 4e). Remarkably, peptides in OMCC often exhibited HDX decrease in a lesser extent and more temporarily than peptides in PRC, as denoted in residues 509–521 of CagX and residues 1891–1903 of CagY. Similar to the HDX-MS analysis on the CagM-CagX complex (Fig. 2c–e), deuterium uptake of CagX was reduced in the β-sandwich of the PRC region and OMD (Figs. 2c and 4c), in line with the homo-oligomerization interfaces of CagX^18^. The extensive buried interface in the PRC region compared to OMCC region (1014 Å^2^ vs. 411 Å^2^) and fortified hydrogen bond strength during CagX self-oligomerization was exemplified by an overall HDX protection in PRC (Fig. 4c). Conversely, significant HDX decrease in CagY_PRC-OMCC_ was found in PRC (residues 1584–1597), PL (residues 1611–1635) and OMCC (residues 1655–1682) regions. After mapping onto the model with PL predicted by AlphaFold3^27^, it was suspected that they were involved in the buried oligomerization interface induced by CagX (Fig. 4d, e and Suppl. Fig. 8a, c). Such HDX decline in homo-oligomerization interface was only observed in complex state but not in their apo state, suggesting that CagY may enhance the stability of the CagX ring and vice versa. These results demonstrated that the stability and integrity of PRC is independent from the PL-OMCC region.

### Real-time HS-AFM reveals CagY-mediated stabilization and sequential assembly of the central cylinder

To understand the full assembly process of the central cylinder, we first evaluated the structural stabilization of CagX rings by CagY under HS-AFM. After visualizing CagX-CagY_PRC-OMCC_ complex formation by adding CagY_PRC-OMCC_ onto immobilized CagX under HS-AFM, we quantified the impact of CagY on the structural stabilization of CagX rings by comprehensive dynamic and static analyses of CagX rings and CagX-CagY_PRC-OMCC_ rings (Suppl. Fig. 22, 23, Suppl. Movie 10). Time-resolved image correlation analysis revealed that CagX rings progressively lost structural coherence over time, whereas CagX-CagY_PRC-OMCC_ rings maintained high Pearson correlation values throughout the observation period (Suppl. Fig. 22c). Structural retention, defined as the mean correlation over the final 25% of observation, was significantly higher for CagX-CagY_PRC-OMCC_ than CagX rings (*p* = 0.0001, Mann-Whitney U test; Suppl. Fig. 22d). The coefficient of variation of circularity was also significantly lower for CagX-CagY_PRC-OMCC_ rings (*p* < 0.0001; Suppl. Fig. 22e, f), indicating that CagY suppresses shape fluctuations. Furthermore, independent static analysis of CagX and CagX-CagY_PRC-OMCC_ ring particles confirmed that CagX-CagY_PRC-OMCC_ rings were significantly taller (*p* < 0.001) and more circular (*p* < 0.001) than CagX rings while the FWHM diameter difference was not significant, possibly masked by tip broadening effects (Suppl. Fig. 23). These quantitative findings demonstrate that CagY binding structurally stabilizes CagX rings, consistent with the HDX-MS data showing reduced conformational dynamics of CagX upon CagY binding (Fig. 4e, peptide 9).

**Fig. 5:**
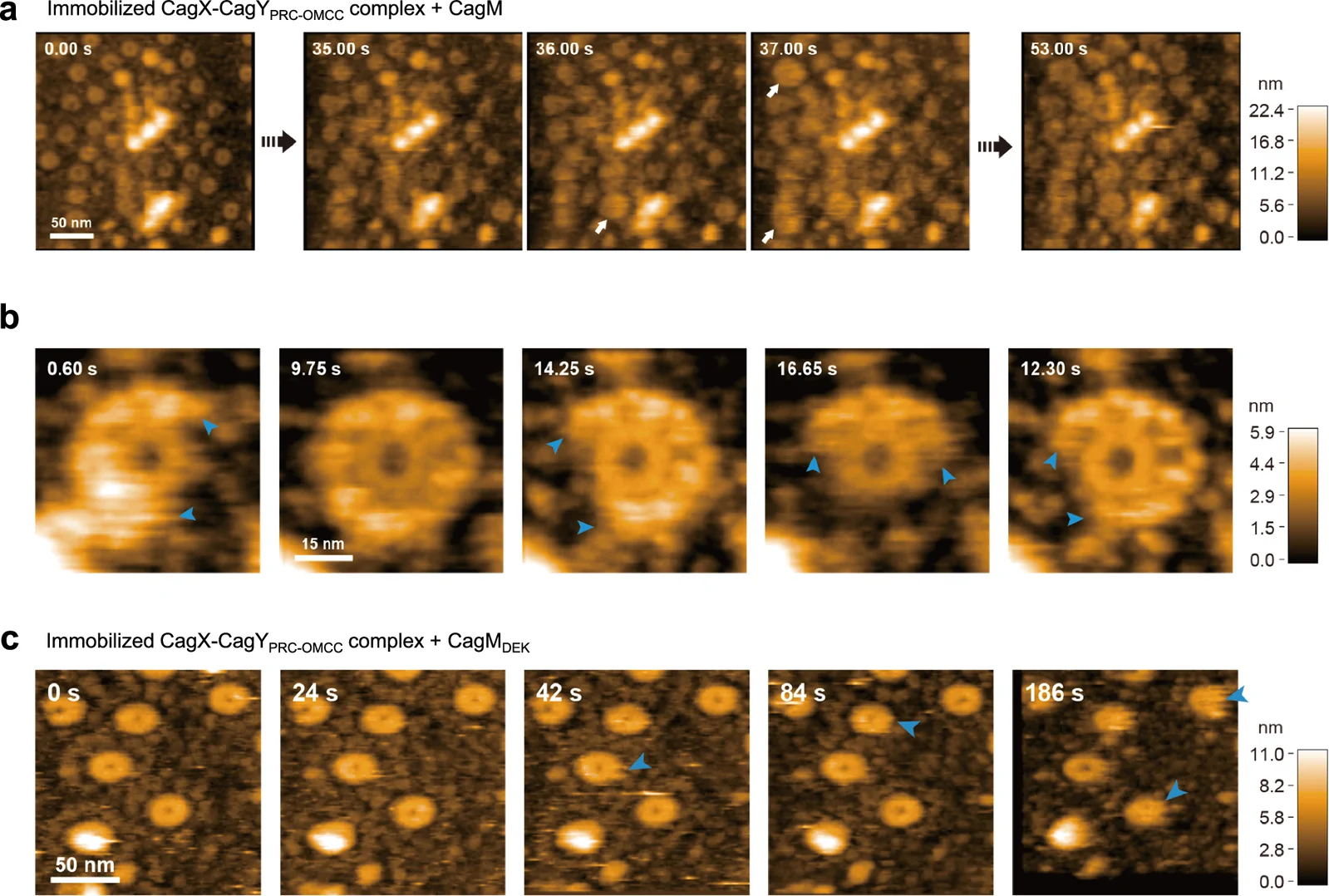
HS-AFM of CagX-CagY_PRC-OMCC_-CagM complex assembly. **a** Wide-field time-lapse HS-AFM images showing the assembly of CagM onto pre-mixed and immobilized CagX-CagY_PRC-OMCC_ complex on bare mica. White arrows indicate that CagM associates with the CagX-CagY_PRC-OMCC_ ring as an intact outer ring, rather than as individual subunits (continuous scanning mode at 1 s/frame). Scale bar, 50 nm. **b** High-resolution time-lapse HS-AFM images of a single CagX-CagY_PRC-OMCC_ complex. Blue arrows indicate dissociation of CagM subunits and openings on the outer ring (continuous scanning mode at 0.15 s/frame). Scale bar, 15 nm. **c** Time-lapse images of adding CagM_DEK_ into pre-mixed and immobilized CagX-CagY_PRC-OMCC_ complex on mica (intermittent scanning mode with 1 frame on and 5 frames off at an imaging speed of 1 s/frame). Blue arrows indicate the binding of CagM onto the inner ring without forming an outer ring. Scale bar, 50 nm.

Previously, isolation of CagX-CagY complex from *H. pylori* c*agM*-null strain was reported^14,28^. Here, we visualized the real-time assembly of the CagM-CagX-CagY complex by immobilizing pre-mixed CagX-CagY_PRC-OMCC_ complex followed by the addition of CagM. We observed the CagM outer ring bursting out from the CagX-CagY_PRC-OMCC_ ring over time, constituting a double-ring structure (Fig. 5a, Suppl. Movie 11). We also observed CagM dissociating from and re-associating with the CagX-CagY_PRC-OMCC_ complex in the form of granular units, behaving similarly as in CagM-CagX complex formation (Fig. 2b, Fig. 5b, Suppl. Movie 12). To further establish the assembly order, we performed sequential addition of CagY_PRC-OMCC_ and then CagM to immobilized CagX, showcasing the successful formation of central cylinder (Suppl. Fig. 24a, Suppl. Movie 13). On the other hand, no significant change in CagM-CagX ring morphology was observed in the reversed-order experiments by adding CagY_PRC-OMCC_ to pre-formed CagM-CagX complexes (Suppl. Fig. 24b, Suppl. Movie 14). Temporal standard deviation analysis confirmed that while CagY was present near the surface (elevated temporal SD: 0.82 nm vs. 0.53 nm for CagM-CagX alone, *p* < 0.001; Suppl. Fig. 25, Suppl. Movie 15), it failed to incorporate into the ring. This supports a model in which CagY incorporation precedes CagM binding, since CagY fails to bind when CagM has already docked on the CagX ring under our in vitro reconstitution condition. In addition, when CagM_DEK_ mutant was applied to the immobilized CagX-CagY_PRC-OMCC_ complex, the double ring structure formed was incomplete (Fig. 5c, Suppl. Movie 16). These data support the findings from the pull-down and TEM studies (Suppl. Figs. 13–15) and validate the role of high-order oligomerization of CagM in constructing the central cylinder.

**Fig. 6:**
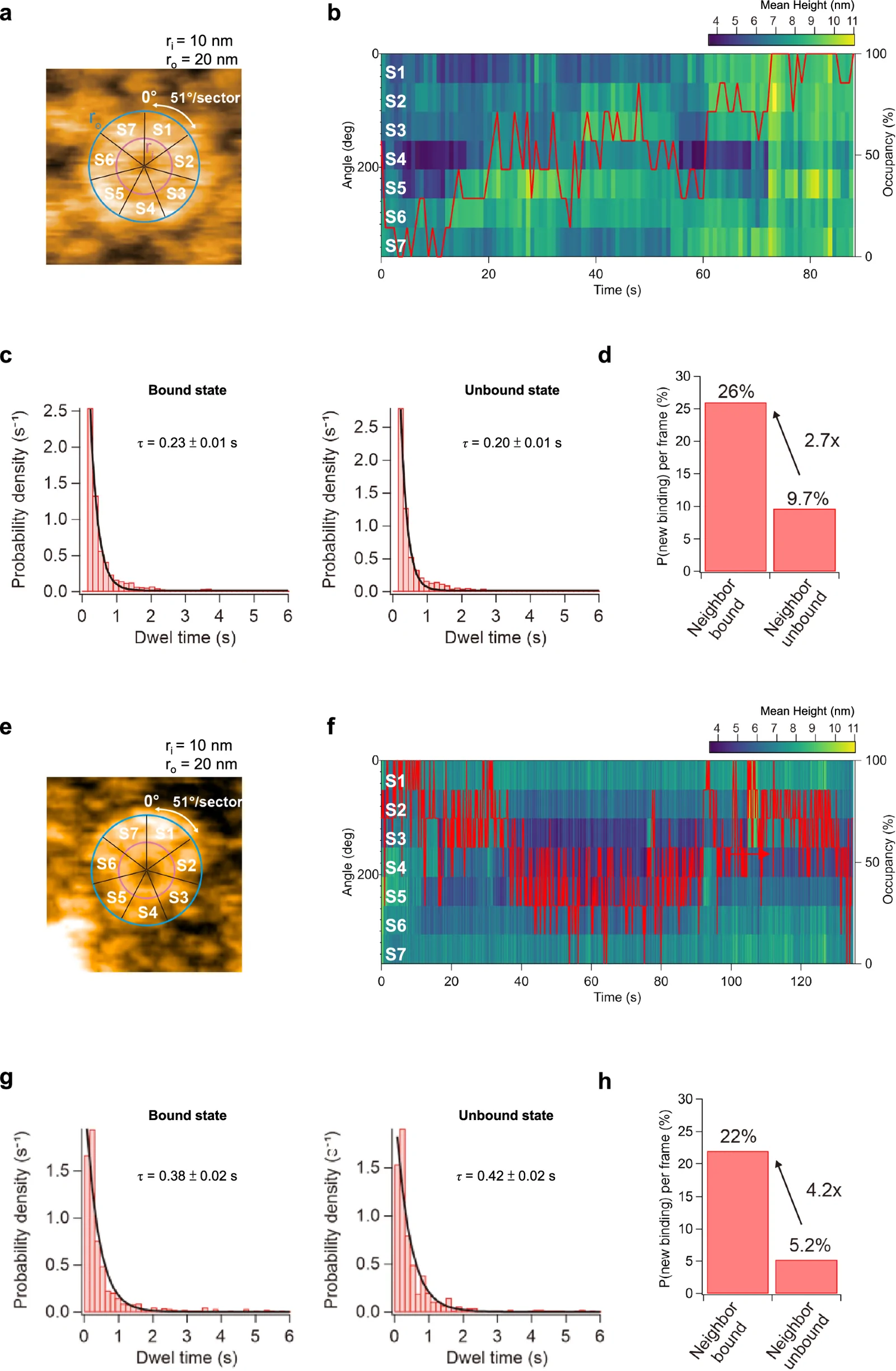
Quantitative dynamics of CagM binding to CagX and CagX-CagY_PRC-OMCC_ rings revealed by HS-AFM. **a** Sector segmentation scheme for angular kymograph analysis of CagM binding onto a CagX ring. The outer annular region (r_i_ = 10 nm, r_o_ = 20 nm) is divided into 7 sectors (51°/sector). **b** Angular kymograph showing the mean height of each sector of the CagX ring over time. High-intensity regions (yellow–green) correspond to CagM-bound sectors and low-intensity regions (blue–purple) to unbound sectors. The red line indicates overall occupancy. **c** Dwell time distributions of bound (τ = 0.23 ± 0.01 s) and unbound (τ = 0.20 ± 0.01 s) states, fitted with single-exponential decay functions. *N* = 2024/2033 events from 10 molecules. **d** Conditional binding probability showing 2.7-fold cooperativity: the probability of new CagM binding when an adjacent sector is occupied (26%) versus unoccupied (9.7%). **e** Sector segmentation scheme for angular kymograph analysis of CagM binding onto a CagX-CagY_PRC-OMCC_ ring (r_i_ = 10 nm, r_o_ = 20 nm; 7 sectors, 51°/sector). **f** Angular kymograph of a CagX-CagY_PRC-OMCC_ ring during CagM binding, with overall occupancy indicated by the red line. **g** Dwell time distributions of bound (τ = 0.38 ± 0.02 s) and unbound (τ = 0.42 ± 0.02 s) states, fitted with single-exponential decay functions. *N* = 951/947 events from 6 molecules. **h** Conditional binding probability showing 4.2-fold cooperativity: the probability of new CagM binding when an adjacent sector is occupied (22.0%) versus unoccupied (5.2%). The longer bound-state dwell time and higher cooperativity on CagX-CagY_PRC-OMCC_ rings compared with CagX rings alone suggest that CagY modulates CagM association/dissociation kinetics and enhances cooperative binding.

To quantify the CagM binding kinetics during the assembly process, we performed an angular kymograph analysis. As CagM-CagX complex exhibits as C7 symmetry in our cryo-EM structure (Fig. 2a), the outer annular region of the CagX ring was divided into seven sectors (51°/sector). CagM binding in each sector was estimated from the mean height within an annular region defined by the approximate outer radius of CagX (r_i_ = 10 nm) and the outer radius of CagM (r_o_ = 20 nm) (Fig. 6a). The angular kymograph and occupancy time course of a representative molecule are shown in Fig. 6b. Although the higher scanning frequency precluded complete double-ring formation due to increased tip perturbation, the kinetic parameters inherently reflect these conditions. Dwell time analysis of 2024 bound-state and 2033 unbound-state events across 10 molecules (imaged at 0.15 s/frame) revealed characteristic timescales of τ_bound_ = 0.229 ± 0.005 s and τ_unbound_ = 0.195 ± 0.005 s (exponential fitting errors) (Fig. 6c), indicating that binding and dissociation occur with approximately equal probability, thus suggesting the system is near equilibrium. Notably, the probability of new CagM binding when an adjacent sector was already occupied (26%) was 2.7-fold higher than when neighbouring sectors were unoccupied (9.7%) (Fig. 6d), demonstrating a positive cooperativity in CagM assembly onto CagX rings. Quantitative dynamics analysis of CagM binding on CagX-CagY_PRC-OMCC_ rings revealed an approximately 1.7-fold longer bound-state dwell time (τ = 0.380 ± 0.016 s, exponential fitting error) and an enhanced cooperativity (4.2-fold) compared to CagX-only rings (Fig. 6e–h), indicating that CagY not only stabilizes the ring scaffold but also promotes the incorporation of CagM.

## Discussion

CagT4SS is indispensable in the translocation of oncogenic protein CagA, and its biogenesis marks a critical determinant in the pathogenesis of *H. pylori*. Although recent structural analyses have revealed the molecular architecture of CagT4SS as well as most of the atomic details in OMCC and PRC substructures, the order of assembly of CagT4SS components and the underlying conformation dynamics remain largely unknown. The central cylinder, composed of CagX, CagY and CagM, is of particular interest, as it serves as the initiator of the nanomachine, contacts with host receptors, and is postulated to function as gateway for the pilus biogenesis and substrates translocation^8,11,13,20,29,30^. In this study, we report the in vitro reconstitution of different subcomplexes in the central cylinder that share key structural features with the native CagT4SS, including the ring diameters and molecular interfaces of native CagT4SS. The findings from different in vitro biophysical analysis further provide temporal and spatial details of the dynamic assembly states beyond the fully assembled PRC-OMCC structure and allow us to propose a sequential assembly order^18^.

**Fig. 7:**
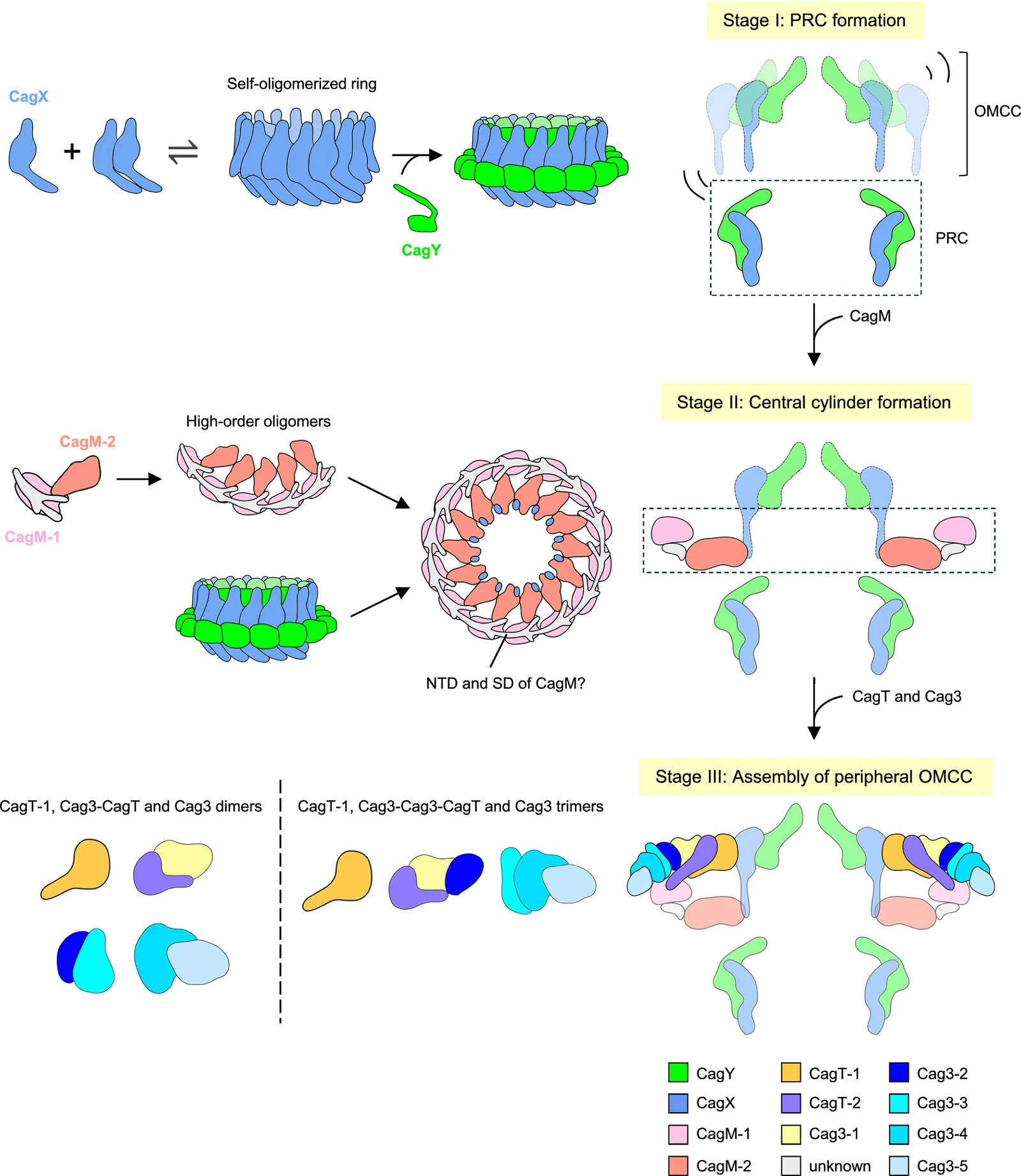
Proposed model of CagT4SS assembly. A schematic diagram depicting a proposed model of PRC-OMCC in CagT4SS assembly. CagX_PRC_ (cornflower blue) self-oligomerizes into ring structure and interacts with monomeric CagY (lime) to form the PRC. CagX-CagY interaction only stabilizes PRC region while OMCC region remains highly flexible. CagM dimers (CagM-1 in light pink and CagM-2 in light coral) undergo high-order oligomerization via NTD and SD, and interact with CagX-CagY complex, displaying a double-ring structure (viewing from the bottom of OMCC). Finally, peripheral component CagT-1 (orange) is associated to the central cylinder followed by dimers and trimers of Cag3-CagT and Cag3, resulting in a fully assembled PRC-OMCC substructure.

Our study starts by assessing the self-oligomerizing properties and dynamic behaviour of CagX, CagY and CagM by TEM and HS-AFM, respectively. The spontaneous formation of ring structure in CagX and characterization of reconstituted sub-structures CagM-CagX, CagX-CagY and CagX-CagY-CagM suggests that CagX serves as the scaffold for the incorporation of CagY and CagM. While CagY does not self-oligomerize, quantitative HS-AFM analysis demonstrated that CagX-CagY_PRC-OMCC_ rings exhibited significantly higher structural retention and lower circularity fluctuation compared to CagX-only rings, providing direct evidence for CagY-mediated stabilization (Suppl. Fig. 22). Further integration of the findings from mutagenesis, HDX-MS and cryo-EM, we propose that the assembly of the PRC-OMCC central cylinder is initiated with self-oligomerization of the PRC region of CagX, followed by coupling of CagY to generate the PRC (Fig. 7). Although the HDX-MS results show that the CagX-CagY interface at OMCC retains, higher binding affinity of CagX-CagY_PRC_ than CagX-CagY_OMCC_ in MST assays and the missing density of the entire OMCC in cryo-EM map suggest that OMCC and the linker connecting PRC and OMCC remain highly flexible in CagX-CagY complex (Fig. 4a, b and Suppl. Fig. 18, 19). These findings may explain previous observation that both CagX and CagY were not co-immunoprecipitated in their respective individual knockout strains^14^. The intact PRC then serves as a molecular hub to facilitate central cylinder formation through docking of CagM to the OMCC-proximal region of CagX. Upon binding, CagM forms the outer rim of the double-ring structure as observed in both cryo-EM and quantitative HS-AFM studies and becomes more stable. As supported by mutagenesis, SEC-SLS, TEM and HDX-MS analysis, association of CagM to form the outer ring formation requires charged-charged interactions at its N-terminus (Figs. 2, 3 and Suppl. Figs. 9a, 10, 13–15). All CagM mutants constructed show interaction with CagX, indicating that they were properly folded. It is unlikely that their disability in CagM-CagX double ring formation was caused by protein misfolding. However, how the charged residues at N-terminus participate in CagM high-order oligomerization will require a high-resolution structural model. Based on our findings and previous fully assembled cryo-EM model, we reason that the CagM dimer composed of CagM-1 and CagM-2 within an asymmetric unit serves as a stable building block for multimerization. As such, CagM-1 containing peripheral ring is formed concurrently during CagM-2-CagX interaction. Likely, the unassigned density in the periphery near CagM-1 in the reported cryo-EM structure corresponds to the N-terminal segment of CagM-1 (Fig. 3d, e)^18^. This proposed order of assembly is also demonstrated by sequential reconstitution under HS-AFM and the enhanced binding kinetics of CagM onto CagX-CagY ring (Fig. 6h and Suppl. Fig. 24a), aligning with previous studies showing that isolation of CagT4SS core complex required the presence of both CagX and CagY, and PRC remained intact in *cagM* and *cagT* knockout strains^14,26^. Assembly of CagX-CagY-CagM to form the central cylinder allows stepwise association of CagT, Cag3-CagT complex and Cag3 oligomers, yielding a complete PRC-OMCC substructure^14,15,26^. We speculate that stabilization of the C-terminal region of CagY promotes oligomerization and hierarchical assembly of its N-terminal 1400-residue region distinct to *H. pylori*. Prior research indicates that this region containing repetitive sequence is intrinsically disordered, and the middle repeat region (MRR) may form an additional ring-like structure beneath PRC^23^. We envisage that the stabilization of CagY_PRC-OMCC_ would facilitate IMC organization and the association with other Cag proteins.

By comparing the previous cryo-EM model of OMCC and our low-resolution cryo-EM map of CagM-CagX complex, the differential arrangement of CagM spokes further indicates the dynamics during the assembly process. In HS-AFM, the cooperative binding of CagM revealed by our angular kymograph analysis provides mechanistic insights into outer ring formation. The 2.7-fold cooperativity on CagX rings (Fig. 6d) suggests that CagM binding induces local structural changes that facilitate adjacent CagM association, possibly through direct CagM-CagM interactions via the NTD or conformational accommodation of the CagX scaffold. Remarkably, this cooperativity was enhanced to 4.2-fold on CagX-CagY_PRC-OMCC_ rings (Fig. 6h), consistent with the stabilized ring scaffold providing a more favourable template for ordered CagM multimerization. The approximately 1.7-fold extension of CagM bound-state dwell time on CagX-CagY_PRC-OMCC_ rings (Fig. 6) further indicates that CagY increases the dissociation barrier for CagM, establishing a kinetic basis for the ordered assembly pathway (Fig. 7). It is important to point out that, in this study, bare mica and APTES-treated mica were screened in parallel. We found that CagM could only bind to CagX immobilized on APTES-treated mica or pre-formed CagX-CagY_PRC-OMCC_ complex on bare mica (Suppl. Fig. 26; Suppl. Movie 17). As shown by localization AFM^31^ and pseudo-AFM analyses, the OMC-to-PR face of the CagM-CagX complex was oriented upward on APTES-treated mica (Suppl. Fig. 27). This orientation-dependent binding is structurally consistent with the known topology of the PRC-OMCC complex, implying that the availability of OMC face is crucial for CagM binding. Similarly, the successful assembly of CagM onto CagX-CagY_PRC-OMCC_ complex is strictly dependent on the proper orientation of the pre-formed complex, as placing it on APTES-mica inverts its orientation and abrogates outer ring assembly (Suppl. Fig. 26f, Suppl. Movie 18). Taken together, our observations revealed that the substrate surface chemistry controls ring orientation rather than oligomerization itself. We reasoned that it involves a complex mechanism, likely combining electrostatic, hydrophobic, contact-area and flexibility effects, with a rigorous dissection acknowledged as beyond the scope of this study.

In summary, our proposed model complements the existing static structures and underscores the importance of PRC in CagT4SS assembly. Owing to the absence of PRC in minimized T4SSs and experimental evidence of OMCC-initiated Dot/Icm T4SS assembly in *L. pneumophila*^25,32^, the PRC-initiated CagT4SS assembly is possibly unique to *H. pylori*. The mechanical coupling of stable PRC may act as an anchoring base, providing structural support for the flexible OMCC and potentially contributing to substrate translocation and pilus formation. Aligning with previous observations, we speculate that the conformational change of OMCC is indispensable for partially unfolded CagA to translocate via the narrow central channel with diameter of ~3.5 nm facing outer membrane and ~10 nm facing inner membrane^17,18,33–35^. In other T4SS, OMCCs composed of only VirB9 (CagX homologue) and VirB10 (CagY homologue) are structurally unstable^25^, suggesting that these two conserved core proteins may share some common structural and functional properties. The in vitro reconstitution of sub-complexes of PRC-OMCC using recombinant proteins provides a tool that overcomes the technical challenges posed by inherent structural flexibility to isolate intermediate subassemblies. For instance, previous LC-MS study detected CagM in *cagT* knockout strain but could not identify its presence in the isolated PRC-OMCC substructure^26^. However, the assembly pathway observed here may not fully recapitulate the process in situ, where additional factors such as the periplasmic and inner and outer membrane environment, other T4SS components may influence the assembly sequence and kinetics. The current low-resolution map of the CagM-CagX complex also limits the elucidation of its atomic details. Future investigation could address these limitations by pursuing higher-resolution structural analysis of the reconstituted complexes and by examining the assembly dynamics during substrate translocation and host cell interactions. Our study also opens avenues for the development of therapeutic strategies on blocking CagT4SS biogenesis directly, leading to a more comprehensive inhibition of *H. pylori* pathogenicity.

## Methods

### Plasmid construction and mutagenesis

Genes of CagM, CagX, and CagY were amplified from the genomic DNA of *H. pylori* G27 strain. Signal peptides were all removed and not counted in mutant nomenclature. Their full-length (without signal peptide) and truncated constructs were cloned into pGEX-6p3 vector consisting of a N-terminal GST tag. CagX constructs were also cloned into pAC28m vector with a 6xHis tag at its N-terminal. Mutations were introduced by site-directed mutagenesis. All plasmids were confirmed by DNA sequencing.

### Protein expression and purification

*E. coli* BL21 (DE3) was used for protein expression. Transformed bacteria were grown at 37 °C and induced with 0.2 mM isopropyl β-d-thiogalactopyranoside (IPTG) at 20 °C until OD reached 0.6. After overnight expression, cells were harvested and resuspended with ice-cold lysis buffer containing 50 mM Tris, 500 mM NaCl, pH 7.5 supplemented with protease inhibitors. It was then subjected to sonication and centrifugation to obtain the soluble fraction. The clear lysate was loaded to the glutathione sepharose pre-equilibrated with lysis buffer and incubated for 2 h at 4 °C with rocking. The beads were washed thoroughly, and PreScission Protease (PP) was added to cleave the GST tag overnight. For His-tagged proteins, 20 mM imidazole was added to the lysis buffer and Ni sepharose was used for affinity chromatography. After washing, lysis buffer with 500 mM imidazole was used for elution. The eluates were concentrated and further purified by either HiLoad 16/600 Superdex 200 pg or HiLoad 16/600 Superdex 75 pg column (Cytiva). For the purification of CagX-CagY_PRC_ complex, pGEX-6p3-CagY_PRC_ and pAC28m-CagX were co-transformed and co-expressed. The purification procedures were similar except that the complex was purified by tandem affinity chromatography: Ni affinity followed by GST affinity column and incubated with PP overnight. Concentrated eluates were injected to Superose 6 increase 10/300 GL column. Fractions with high purities were concentrated in 20 mM HEPES, 300 mM NaCl, pH 7.5 buffer and used for subsequent analysis.

### Cross-linking experiments

Purified CagM at 0.5 mg/ml concentration and 100-fold molar excess of disuccinimidyl sulfoxide (DSSO, ThermoFisher Scientific) were mixed and incubated on ice for 2 h. A final concentration of 20 mM Tris was added to quench the reaction.

### Negative stain electron microscopy sample preparation and data processing

Purified proteins were diluted to 0.05–0.08 mg/ml for negative staining. 7 μl of protein aliquots were applied to glow-discharged, carbon-coated 300-mesh copper grids (Ted Pella) for 30 s. Grids were then stained with 1% uranyl acetate for 30 s. The samples were examined under transmission electron microscope H-7650 (Hitachi) operated at 80 kV. Images were manually collected using NanoSprint12 camera system (Advanced Microscopy Techniques).

The micrographs of cross-linked CagM were first processed in EMAN2^36^. Subsequently, they were subjected to manual picking in Relion 4.0.1^37^. Reference-free 2D classification was performed using a total of 2737 particles.

### Size exclusion chromatography/static light scattering (SEC-SLS)

Protein samples were injected into either Superdex 200 increase 10/300 GL or Superose 6 increase 10/300 GL column (Cytiva) pre-equilibrated with buffer containing 20 mM HEPES, 300 mM NaCl, pH 7.5. The system was connected to an in-line miniDawn light scattering detector and an Optilab DSP refractometer (Wyatt Technology). The molecular masses of protein samples were determined using ASTRA software package (Wyatt Technology).

### Analytical ultracentrifugation

The sedimentation velocity analysis was performed using Beckman Coulter ProteomeLab XL-I analytical ultracentrifuge equipped with a 60-Ti rotor. Around 1 mg/ml of purified protein was loaded and spun at 36,288 x g at 16 °C. The buffer containing 20 mM HEPES, 300 mM NaCl, pH 7.5 was used as reference. Absorbance at 280 nm was continuously measured for 300 scans. The data was analyzed by Sedfit^38^ using continuous sedimentation coefficient distribution model.

### Co-incubation assays

Purified proteins were mixed at 1:1 molar ratio and incubated at 4 °C overnight. The mixture was injected into Superose 6 increase 10/300 GL column (Cytiva) pre-equilibrated with 20 mM HEPES, 300 mM NaCl, pH 7.5 buffer. Individually purified proteins were run as control. The eluted fractions were then analyzed by SDS-PAGE.

### Pull-down assays

Bacterial cells transformed with corresponding plasmids were grown, expressed and lysed similarly as mentioned above. To define the CagM-CagX interaction interface in CagM, GST-CagX was bound onto glutathione sepharose beads for 2 h at 4 °C. The beads were then washed. Truncated and mutated CagM constructs were added with the same protein amount as the bait and incubated for 2 h in the GST binding buffer of 20 mM HEPES, 300 mM NaCl 4 mM DTT, pH 7.5. GST binding buffer with 0.1% Tween 20 was used as washing buffer to reduce non-specific binding. Controls of all CagM constructs binding to immobilized GST and GST beads alone were set, respectively. On the other hand, the interface in CagX was mapped using His-CagX constructs as bait and immobilized on Ni sepharose. Procedures were similar as mentioned above except binding buffer of 20 mM HEPES, 300 mM NaCl, 40 mM imidazole, pH 7.8 was used. CagM was then added in 1:1 amount for interaction and binding buffer in addition to 0.1% Tween 20 was used to wash the beads. A control of CagM mixed with Ni sepharose was set. For the interaction between CagX and CagY, GST-CagY constructs were immobilized to GST beads similarly as GST-CagX above. After washing, different CagX truncations with 2-fold excess in amount were added. 0.25% Tween 20 was supplemented to GST binding buffer for washing. Controls were also set up in parallel by adding CagX into immobilized GST. All washed beads were then resuspended and loaded onto SDS-PAGE for analysis.

### Hydrogen-deuterium exchange mass spectrometry (HDX-MS)

All HDX-MS experiments were performed on a nanoACQUITY UPLC system coupled with Synapt G2-Si Mass Spectrometer (Waters Corporation). Reactions comparing wild-type and mutated CagM were conducted with a final protein concentration of 2 μM. Hydrogen-deuterium exchange was initiated by mixing 2 μl of CagM with 48 μl of deuterated buffer containing 20 mM HEPES, 300 mM NaCl, pD 7.5 and 96% D_2_O (Cambridge Isotope Labs). While for reactions comparing CagM and CagX in their apo- and complex-states, 3 μl of protein was mixed with 47 μl of deuterated buffer with 94% D_2_O, yielding a protein concentration of 1.2 μM. The same protein concentration was used for reactions comparing CagX and CagY. The mixtures were incubated for various time periods (0.5, 1, 10, 60, and 120 min) at room temperature and quenched by adding equal volume of ice-cold quenching buffer, resulting in a final pH of 2.5. For non-deuterated samples, same procedures were performed by replacing the deuterated buffer by buffer containing 20 mM HEPES, 300 mM NaCl, pH 7.5. Quenched protein mixtures were immediately digested online by Waters Enzymate Protein Pepsin column (300 Å, 5 µm, 2.1 mm X 30 mm) at a flow rate of 100 μl/min. The peptides were trapped on a Waters ACQUITY UPLC BEH C18 VanGuard Pre-column (130 Å, 1.7 µm, 2.1 mm X 5 mm) for 3 min before being applied to a Waters ACQUITY Premier BEH C18 column (1.7 µm, 2.1 x 100 mm). Peptides were eluted in an acetonitrile gradient of 0–0.5 min: 92% A/8% B, 0.5 to 6.5 min: 92% A/8% B to 60% A/40% B, 6.5–8 min: 60% A/40% B to 5% A/95% B and 8 min–12 min: 5% A/95% B, in which solvent A was 0.1% formic acid and solvent B was 0.1% formic acid in acetonitrile. The whole trapping and chromatographic process was kept at 0 °C to minimize back exchange. Purified peptides were electrosprayed onto the mass spectrometer in MS^E^ mode and the mass spectra were acquired in the range of 100–3000 m/z. The instrument was calibrated using sodium iodide and lock mass accuracy correction was made by intermittent infusion of leucine enkephalin. All samples were performed in triplicate.

### HDX-MS data analysis

Peptides in non-deuterated samples were identified using ProteinLynx Global Server 3.0.2 (Waters). They were searched against the database containing their primary sequences and filtered by the following parameters: intensity > 1000, number of product ions per amino acids > 0.25, PLGS score > 6 and precursor ion (MH + ) mass error < 10 ppm. Data was manually inspected via DynamX 3.0 (Waters). Relative deuterium uptake between two states were determined by calculating the deuterium uptake differences of the identical peptides. Statistical significance was determined by 95% confidence interval. The deuterium uptake of each peptide was mapped onto the protein structure by PyMol 3.1.1^39^.

### Microscale thermophoresis (MST)

Purified CagX was fluorescently labelled using Alexa Fluor 647 NHS Ester dye (Thermo Fisher Scientific). Serial dilution of CagY (PRC-OMCC: 5.42 μM to 0.165 nM; PRC: 6.14 μM to 0.187 nM; OMCC: 143 μM to 4.35 nM) was performed and added to 20 nM of labelled CagX in a volume ratio of 1:1. 20 mM HEPES, 300 mM NaCl, 0.05% Tween 20, pH 7.5 was used as the reaction buffer. The assay was conducted using Monolith NT.115 instrument (NanoTemper Technologies) at 25 °C. The excitation power and MST power were adjusted to 20% and medium respectively. Experiments were performed in triplicate. The K_d_ value and its confidence level were determined using MO. Affinity v2.2.6 (NanoTemper Technologies). Statistical significance in K_d_ difference was calculated by Student’s t-test.

### Cryo-EM sample preparation and image acquisition

Cryo-EM grids were prepared using Vitrobot Mark IV (Thermo Fisher) operated at 4 °C and 100% humidity. UltrAuFoil R1.2/1.3 grids were glow discharged at 15 mA current for 1 min. Then, 4 μl of sample aliquots were applied onto the glow-discharged grids. Protein concentrations of CagM-CagX, CagX, CagX-CagY_PRC_ and CagX-CagY_PRC-OMCC_ at 1 mg/ml, 2.5 mg/ml, 3 mg/ml and 1.6 mg/ml were used respectively. The blotting time of CagM-CagX and CagX-CagY_PRC-OMCC_ is 4 s while CagX and CagX-CagY_PRC_ were blotted for 4.5 s and 3 s. After blotting, grids were plunged frozen directly into liquid ethane. Data acquisition was performed using Titan Krios microscope (Thermo Fisher) operated at 300 kV, equipped with a GIF Quantum energy filter and a Gatan K3 direct electron detector. Images were automatically collected using EPU software (Thermo Fisher) with a slit width of 20 eV on the energy filter and in the super-resolution counting mode at a nominal magnification of x105,000. The calibrated pixel size was 0.83 Å except for CagX-CagY_PRC_ (0.855 Å). The defocus range was set from −1.5 to −2.5 μm. Total electron dose for each movie stack was 50 e/Å^2^. To alleviate the preferred orientation problem, extra tilted datasets were collected for CagM-CagX (at 30° and 40° tilts), CagX (at 20° and 40° tilts), CagX-CagY_PRC_ (at 40° tilt) and CagX-CagY_PRC-OMCC_ (at 30° and 40° tilts) using SerialEM^40^.

### Cryo-EM data processing

Collected movies were motion-corrected by MotionCor2^41^ and images were imported to cryoSPARC v4.5.0^41^ for image processing. Contrast transfer function (CTF) estimation was performed using CTFFIND4^42^ and patch CTF estimation for non-tilted and tilted datasets respectively. Particles from non-tilted dataset were first auto-picked and processed with 2D classification. After several rounds of 2D classification, a subset of selected particles was used as a training set for Topaz^43,44^. Particles were re-picked from the micrographs in all datasets using trained Topaz model and subjected to 2D classification. Noise and junk particles were removed after iterative rounds of 2D classification. To generate a 3D map of CagM-CagX and CagX-CagY_PRC-OMCC_, ab-initio reconstruction and heterogeneous refinement were performed. The best class was further refined by non-uniform and local refinement with symmetry imposed. An additional 3D variability analysis was performed on symmetry-expanded CagM-CagX particles and the best cluster was used to generate a CagM-focused mask. After particle orientation rebalancing and duplicates removal, local refinement was performed with CagM-focused mask and symmetry imposed.

For CagX, a total of 6337 micrographs were collected, and the final round of 2D classification contained 10,128 particles. For CagX-CagY_PRC_ complex, 6482 micrographs were collected and the final round 2D classification contained 162,329 particles. For CagM-CagX complex, a total of 1,767,379 particles were picked from 11,335 micrographs collected at 0°, 30°, and 40° tilt. In particular, CagM-CagX particles were down-sampled to a pixel size of 1.494 Å and the final round of 2D classification contained 403,728 particles. The selected classes were subjected to ab-initio reconstruction. After heterogeneous refinement, one class showed intact structural features subsequently subjected to non-uniform and local refinement with C7 symmetry. The particles were further symmetry expanded and used for 3D variability analysis. To study the arrangement of CagM spokes at the outer ring, a CagM-focused mask was created on the best cluster using ChimeraX. Before the final map generation, particle orientation rebalancing was performed to mitigate the preferred orientation issue followed by subsequent duplicate removal. The final CagM-CagX map was refined using 190,251 particles supplied with the CagM-focused mask and C7 symmetry, generating a map with an overall resolution of 7.77 Å (EMD-69626). For CagX-CagY_PRC-OMCC_ complex, a total of 1,277,522 particles were initially picked from 9058 micrographs collected at 0° and 30° tilt. Selected classes after 2D classification were subjected to ab-initio reconstruction. Among the three classes, the best class with 373,053 particles was selected and performed non-uniform refinement with C17 symmetry followed by local refinement. A C17 map with an overall resolution of 2.58 Å was yielded (EMD-65048). An additional dataset collected at 40° tilt containing 1044 micrographs was collected. 454,267 particles were picked and subjected to 2D classification. Duplicated particles from ab-initio construction were removed and merged with the selected particles in the new dataset, yielding 1,066,541 particles. Heterogeneous refinement was performed on the selected classes, and the best class with 406,996 particles was used for non-uniform refinement imposed with C16 symmetry. After local refinement, a C16 map with an overall resolution of 2.87 Å was yielded (EMD-65051).

### Model building and coordinate refinement

The initial CagX-CagY_PRC-OMCC_ model was constructed using previously published PRC cryo-EM model (PDB ID: 6X6J)^18^. An ASU from the C16 map was first segmented in ChimeraX v1.8, followed by the fitting the one-ASU model into the segmented map in Phenix v1.21.1^45^. Cycles of model building and refinement were performed using Coot v0.9.6^46^ and real_space_refine in Phenix. The model in segmented C16 map was then manually docked into densities of the remaining ASUs and further fitted using map_to_model in Phenix. For the C17 map, the initial model was directly fitted into the map using map_to_model in Phenix. After several rounds of refinement, the final refined models were validated using MolProbity^47^. The protein coordinates for C16 and C17 models have been deposited into Protein Data Bank (PDB ID: 9VGU and 9VGF).

### High-speed atomic force microscopy (HS-AFM)

HS-AFM observations were performed using a laboratory-built instrument based on tapping-mode AFM. For AFM, an Olympus BL-AC7 cantilever was used, with specifications including a spring constant of approximately 0.2 N/m, resonant frequencies ranging from 500 to 800 kHz, and a Q-value of about 2 in aqueous environments. The cantilever’s native apex lacks the requisite sharpness for high-resolution AFM imaging; therefore, amorphous carbon was deposited to construct a columnar protrusion on the apex via focused electron beam exposure in a scanning electron microscope. The apex’s sharpness was subsequently refined by plasma etching in argon gas, thus ensuring the acuity necessary for high-resolution imaging^48^.

All HS-AFM image analyses, including pseudo-AFM image generation, ring stability quantification, angular kymograph analysis, and temporal standard deviation analysis, were performed using custom-developed Python-based software (pyNuD).

As substrates, freshly cleaved mica substrates or mica substrates treated with 0.1% APTES (3-aminopropyltriethoxysilane) were used. Bare mica was used for observations of isolated CagX monomers, while APTES-treated mica was used for isolated CagY monomers to achieve appropriate surface adsorption. For CagX ring formation and CagM binding onto CagX rings, APTES-treated mica was used, which is inferred to orient the OMC face of the CagX ring upward, allowing CagM access to its binding site. Notably, CagY binding reverses the ring orientation on each substrate; therefore, bare mica was used for CagM binding onto CagX-CagY_PRC-OMCC_ rings to maintain the OMC face upward. The specific substrate and buffer conditions for each experiment are detailed below.

Two primary imaging modes were utilized: the standard continuous scanning mode and an intermittent scanning mode. The latter was used to minimize probe-induced perturbations during assembly processes, particularly when imaging mechanically fragile CagX rings and CagM mutant interactions. In the intermittent scanning mode, after acquiring one frame, the scan was paused for a defined number of frame intervals (typically 3–5) before imaging the next frame, repeating this cycle of imaging and pausing. The imaging mode used will be specified for each dataset.

### Detailed sample and buffer conditions for HS-AFM observations

The specific sample concentrations, buffer compositions, and incubation conditions for each experiment in HS-AFM observations are detailed below. Unless otherwise specified, protein samples were diluted in the following buffers and applied to the designated substrates. Basic buffer compositions included 20 mM HEPES, 100 mM NaCl (pH 7.2) referred to as Buffer A, and 20 mM HEPES, 300 mM NaCl (pH 7.5) referred to as Buffer B.

For isolated protein observations, 0.05 μM CagM was diluted in Buffer A and applied onto a bare mica substrate. Similarly, 0.05 μM CagX was diluted in Buffer A and applied onto a bare mica substrate. For isolated CagY_PRC-OMCC_ observations, 0.05 μM CagY_PRC-OMCC_ was diluted in Buffer A and applied onto a 0.1% APTES-treated mica substrate. For the observation of CagM oligomerization dynamics, 0.6 μM CagM was prepared in Buffer B and added onto 0.1% APTES-treated mica. For immobilized CagX observations, 3.33 μM CagX was prepared in Buffer B and immobilized onto 0.1% APTES-treated mica for 3 min. Immobilized CagM observations involved preparing 10 μM CagM in Buffer B and immobilizing it onto a bare mica substrate for 1 min.

In real-time observation of complex formation, experiments involving the addition of CagM to immobilized CagX were performed by immobilizing 3.33 μM CagX onto 0.1% APTES-treated mica for 3 min (in Buffer B), followed by the addition of 142 nM CagM. Similarly, for the addition of CagM_NTD-EK_ to immobilized CagX, 3.33 μM CagX was immobilized onto 0.1% APTES-treated mica for 3 min (in Buffer B), followed by the addition of 714 nM CagM_NTD-EK_. Experiments involving the addition of CagM_DEK_ to immobilized CagX were carried out by immobilizing 3.33 μM CagX onto 0.1% APTES-treated mica for 3 min (in Buffer B), followed by the addition of 571 nM CagM_DEK_. The addition of CagM to pre-mixed and immobilized CagX-CagY_PRC-OMCC_ complex (pre-mixed method) involved pre-mixing CagX and CagY_PRC-OMCC_ in a 1:1 molar ratio (10 μM) in Buffer B, immobilizing 1 μM of this mixture onto bare mica, and then adding 571 nM CagM. Analogously, for the addition of CagM_DEK_ to the pre-mixed and immobilized CagX-CagY_PRC-OMCC_ complex, CagX and CagY_PRC-OMCC_ were pre-mixed in a 1:1 molar ratio (10 μM) in Buffer B, 1 μM of the mixture was immobilized onto bare mica, and then 571 nM CagM_DEK_ was added.

For the additional sequential experiment, 3.33 μM CagX was immobilized onto 0.1% APTES-treated mica, followed by the addition of 0.3 μM CagY_PRC-OMCC_. After CagY_PRC-OMCC_ association was confirmed, 0.3 μM CagM was subsequently added to observe the complete assembly of the central cylinder. For the reverse-order experiment, CagM-CagX double-ring complexes were first assembled on APTES-treated mica as described above, followed by the addition of CagY_PRC-OMCC_ at 0.3 μM. As no structural change was observed, the concentration was increased to 0.6 μM, but no incorporation was detected at either concentration. For temporal standard deviation analysis, CagM-CagX rings were imaged before and after the addition of 0.6 μM CagY_PRC-OMCC_.

For substrate-dependent orientation controls, CagM addition to CagX rings on bare mica and CagM addition to pre-mixed CagX-CagY_PRC-OMCC_ on APTES-treated mica were also performed under the same protein concentrations as described above for the respective complexes, but with reversed substrates. For dynamic stability comparison of CagX and CagX-CagY_PRC-OMCC_ rings, pre-formed rings were imaged by continuous scanning at 0.15 s/frame.

### Construction of pseudo-AFM images

Pseudo-AFM images were generated based on collision simulations between the atomic coordinates of protein structures and a model AFM probe. The low-resolution cryo-EM map of CagM-CagX complex obtained in this study (EMD-69626) was used as the coordinate data. The AFM probe was modelled as a cone with a 10-degree angle and a tip radius of 1.0 nm. To approximate the spatial resolution of the actual HS-AFM images, a low-pass filter with a cutoff spatial frequency of 0.5 nm⁻¹ was applied to the generated images.

### Ring stability analysis

Structural stability of CagX and CagX-CagY_PRC-OMCC_ rings was quantified by tracking image correlation and circularity over time. For each ring particle, Pearson correlation coefficients were calculated between the first frame and all subsequent frames to monitor structural coherence. Structural retention was defined as the mean correlation over the final 25% of the observation period. Circularity was calculated as 4πA/P², where A and P are the area and perimeter of the ring boundary detected by thresholding. The coefficient of variation (CV) of circularity over time was used to quantify shape fluctuations. Statistical comparisons between CagX (n = 15) and CagX-CagY_PRC-OMCC_ (*n* = 11) rings were performed using the Mann-Whitney U test.

### Angular kymograph and dwell time analysis

For quantitative analysis of CagM binding dynamics, the outer annular region of the ring (inner radius r_i_ = 10 nm, outer radius r_o_ = 20 nm) was divided into 7 sectors (51°/sector). The inner and outer radii were chosen based on the structural models of CagX and the CagM-CagX complex to define an annular region covering the CagM-binding zone. The same values were applied to both CagX and CagX-CagY ring analyses, as the measured FWHM diameters of the two rings were not significantly different under HS-AFM (Suppl. Fig. 23), ensuring that the kinetic comparison is not biased by the choice of geometry. Mean height in each sector was calculated for each frame and visualized as an angular kymograph. Bound and unbound states were determined by thresholding the mean height. Dwell times for bound and unbound states were extracted and fitted with single-exponential decay functions. Cooperativity was assessed by comparing the conditional binding probability of a sector when its adjacent sector was bound versus unbound. Analyses were performed on 10 CagX ring molecules (2024 bound-state and 2033 unbound-state events) and 6 CagX-CagY_PRC-OMCC_ ring molecules (951 bound-state and 947 unbound-state events).

### Temporal standard deviation analysis

For analysis of CagY binding to CagM-CagX complexes, temporal mean and temporal standard deviation (SD) maps were computed from HS-AFM image sequences. Comparison of temporal SD values between CagM-CagX only and CagM-CagX + CagY_PRC-OMCC_ conditions was performed using the Mann-Whitney U test.

### Statistics and reproducibility

Determination of sample sizes follows standard practice and the specific n values are given in the corresponding figure legends. All experiments reported in this study were reproduced at least twice. For ring-based quantifications in HS-AFM analysis, only particles displaying a closed annular structure at the first analysed frame and remaining within the field of view throughout the observation period were included; partially imaged, drifting, or fragmented particles were excluded. Frames affected by tip detachment or severe scan artefacts were also excluded. The experiments were not randomized, as conditions were defined by the protein composition added to the substrate; the investigators were not blinded, since the identity of the protein samples had to be known to the operator during imaging. To minimize operator-dependent bias, all quantitative metrics were extracted automatically using the same custom Python software (pyNuD) under identical parameter settings for all conditions. Each HS-AFM imaging condition was reproduced in at least two independent experiments, and the molecule counts reported in the figure legends pool data acquired across these sessions. Statistical comparisons between two groups were performed using the two-sided Mann-Whitney U test, and dwell-time distributions were fitted with single-exponential decay functions by least-squares regression using Igor Pro (WaveMetrics).

### Reporting summary

Further information on research design is available in the Nature Portfolio Reporting Summary linked to this article.

## Supplementary information

Supplementary Information

Description of Additional Supplementary Files

Supplementary Data 1

Supplementary Data 2

Supplementary Data 3

Supplementary Data 4

Supplementary Data 5

Supplementary Data 6

Supplementary Movie 1

Supplementary Movie 2

Supplementary Movie 3

Supplementary Movie 4

Supplementary Movie 5

Supplementary Movie 6

Supplementary Movie 7

Supplementary Movie 8

Supplementary Movie 9

Supplementary Movie 10

Supplementary Movie 11

Supplementary Movie 12

Supplementary Movie 13

Supplementary Movie 14

Supplementary Movie 15

Supplementary Movie 16

Supplementary Movie 17

Supplementary Movie 18

Reporting Summary

Transparent Peer Review file

## Source data

Source Data

## Data Availability

The cryo-EM maps generated in this study have been deposited in Electron Microscopy Data Bank under accession code EMD-69626 (CagM-CagX complex), EMD-65051 (CagX-CagY complex in C16 symmetry) and EMD-65048 (CagX-CagY complex in C17 symmetry). The atomic coordinates have been deposited in Protein Data Bank under accession code 9VGU (CagX-CagY complex in C16 symmetry) and 9VGF (CagX-CagY complex in C17 symmetry), respectively. Previously published structures used in this study are available in the Protein Data Bank under accession code 6X6S (OMCC) and 6X6J (PRC). The HDX-MS data generated in this study have been deposited to the ProteomeXchange Consortium via the PRIDE^49^ partner repository with the dataset identifier PXD075968 (CagM-CagX complex), PXD075969 (CagM mutants) and PXD075970 (CagX-CagY complex), respectively. All additional data generated in this study are provided in the Source Data file. Source data are provided within the Source Data file. Source data are provided with this paper.

## Code availability

All HS-AFM image analyses in this study — including pseudo-AFM image generation, ring stability quantification, angular kymograph and dwell-time analysis, temporal standard deviation analysis, and radial profile comparison — were performed using pyNuD, a Python-based software for high-speed AFM image analysis. The pyNuD installer is publicly available on GitHub [https://github.com/uchihast/pyNuD-installer]. The custom analysis plug-in developed for this study, which implements all study-specific analysis routines, has been deposited at Zenodo [10.5281/zenodo.20779251]^50^. Both are released under an open-source license approved by the Open Source Initiative.
