# Supplementary material for: Deciphering the initiation pathway of CagT4SS assembly by in vitro reconstitution of subcomplexes
Source: Nat Commun. 2026 Jul 23;17:8987. doi: 10.1038/s41467-026-75820-0 (PMC13507270; doi:10.1038/s41467-026-75820-0)
Supplement: Supplementary file 1 [file 41467_2026_75820_MOESM1_ESM.pdf]

Supplementary information for

Deciphering the initiation pathway of CagT4SS assembly by *in vitro* reconstitution of subcomplexes

Hoi Yee Chu, Chin Yu Mok, You-Rong Lin, Huawei Zhang*, Takayuki Uchihashi* and Shannon Wing Ngor Au*

*Co-correspondence: hw.zhang@siat.ac.cn, uchihast@nagoya-u.jp, shannon-au@cuhk.edu.hk

This PDF file includes:

- Supplementary Figures 1-27
- Supplementary Tables 1 and 2

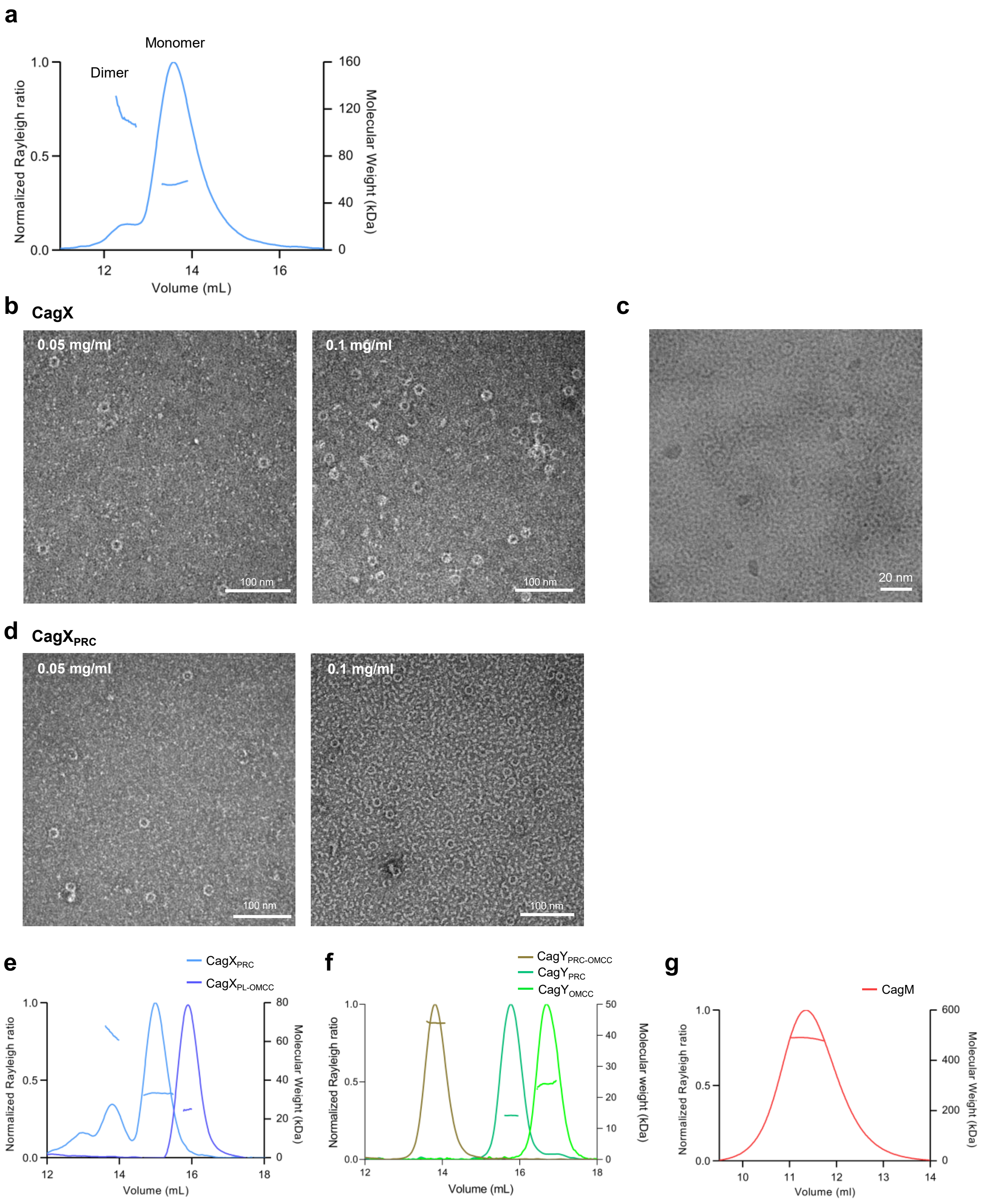

Supplementary Figure 1. Characterization of CagX, CagY and CagM on ring formation. a SEC-SLS analysis of CagX showing it exhibits as dimer and monomer. **b** Representative negative stain EM micrographs of CagX in different concentrations. **c** Representative cryo-EM micrograph of CagX. **d** Representative negative stain EM micrographs of $CagX_{PRC}$ in different concentrations. **e** Oligomeric states of $CagX_{PRC}$ and $CagX_{PL\text{-}OMCC}$ characterized by SEC-SLS showing only $CagX_{PRC}$ exists as both dimer and monomer, similar to CagX. **f** SEC-SLS analysis of CagY constructs demonstrating they all exist as monomers. **g** CagM displays as high-order oligomer as analyzed by SEC-SLS.

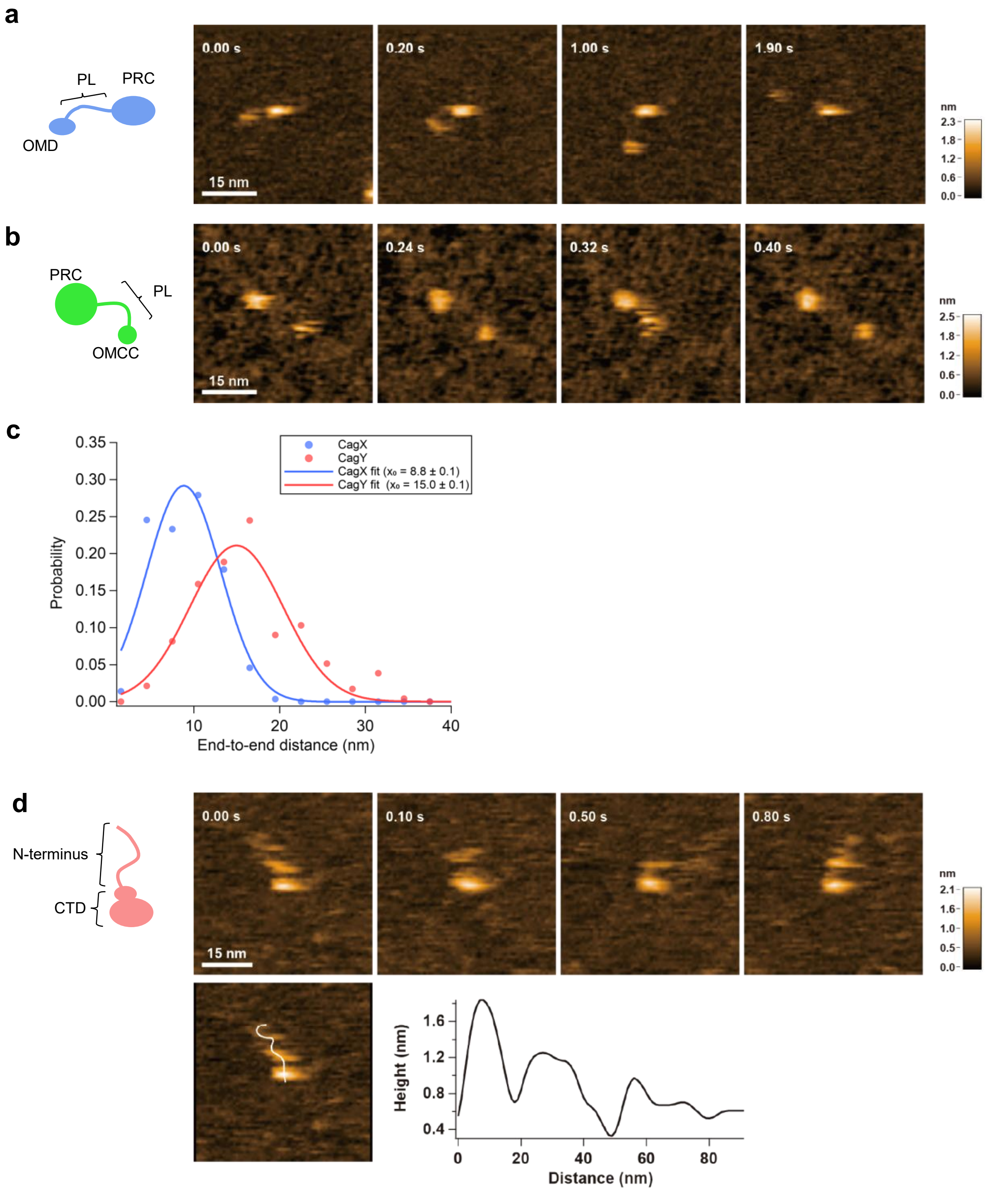

Supplementary Figure 2. HS-AFM of monomeric CagX, CagY and CagM. a HS-AFM images of CagX immobilized on bare mica showing two globular domains connected by a linker. Schematic representation of the observed CagX, in which the larger globular domain, smaller globular domain and linker may correspond to the PRC, OMD and PL, respectively. **b** HS-AFM images of $CagY_{PRC\text{-}OMCC}$ immobilized onto APTES-treated mica showing a globular structure linked to a flexible domain. Schematic representation of the two domains observed in CagY which likely correspond to PRC and PL-OMCC domains, respectively. **c** Histograms of end-to-end distance measured between the two domains in snapshots of HS-AFM movies for CagX (n = 566) and CagY (n = 233). The solid lines represent fit curves following a Gaussian distribution. Values are expressed as mean ± Gaussian fitting error. **d** HS-AFM images of CagM immobilized on mica and the schematic representation of the observed CagM. The two domains likely correspond to the CTD while the flexible tail is contributed by the N-terminus. A representative distance profile of the tail measured along the white line is shown. The average contour length of the tail is 24.4 ± 4.6 nm (s.d., n = 35).

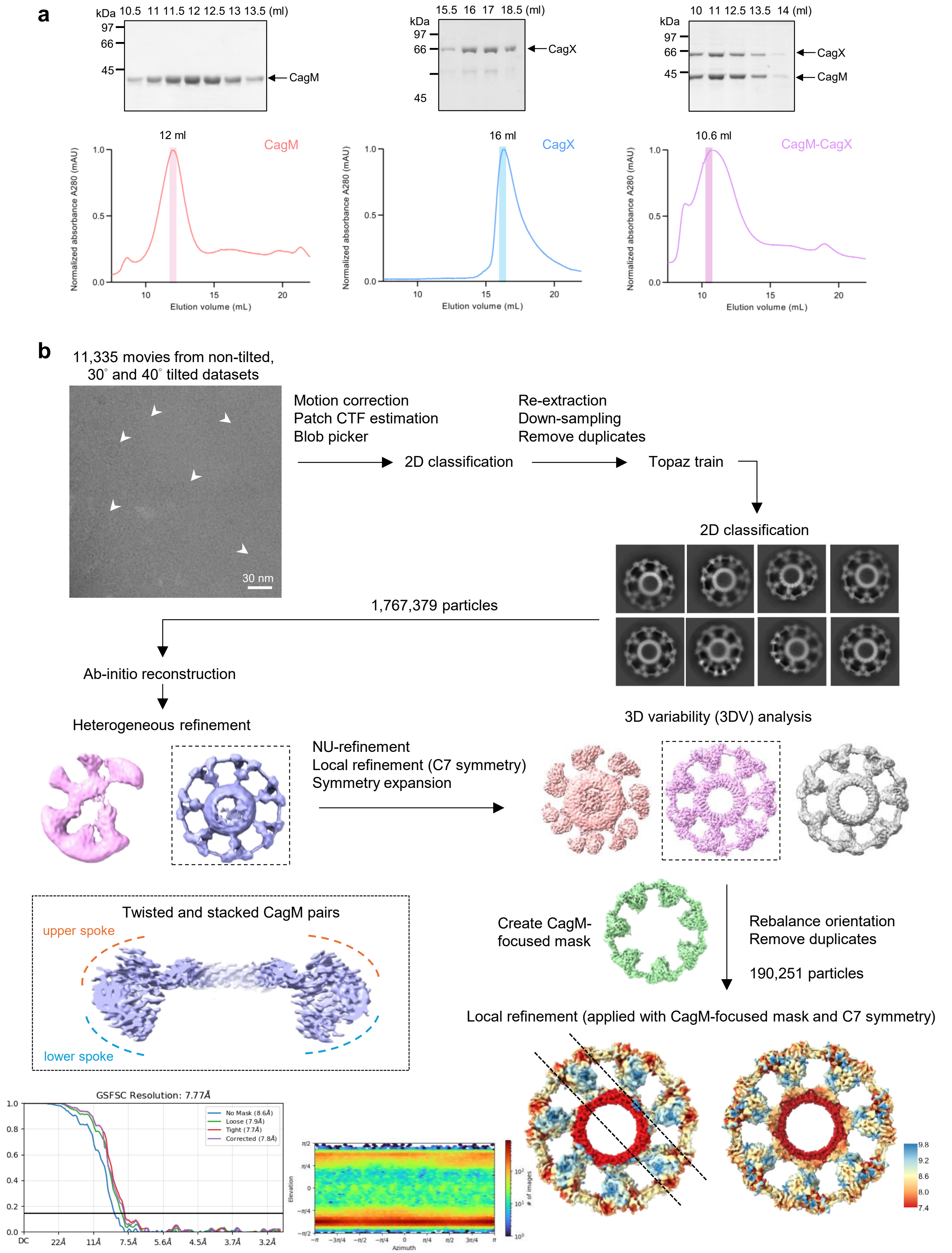

Supplementary Figure 3. Purification and single particle cryo-EM processing workflow of CagM-CagX complex. a Elution profiles and SDS-PAGE analysis of purified CagM, CagX and co-incubated CagM-CagX complex after Superose 6 column. An early shift in elution volume is observed in CagM-CagX complex. **b** Representative cryo-EM micrograph of CagM-CagX complex showing double-ring particles as indicated in arrows. The resolution of CagM-CagX map with CagM-focused mask was determined according to Fourier shell correlation (FSC) with a criterion of 0.143. 2D angular distribution is shown. The map is colored by local resolution. The cross-sectional view of CagM-CagX map (clipping planes indicated in dash lines) shown in the inset box highlighting the twisted and stacked pairs of CagM.

Immobilized CagX + CagM

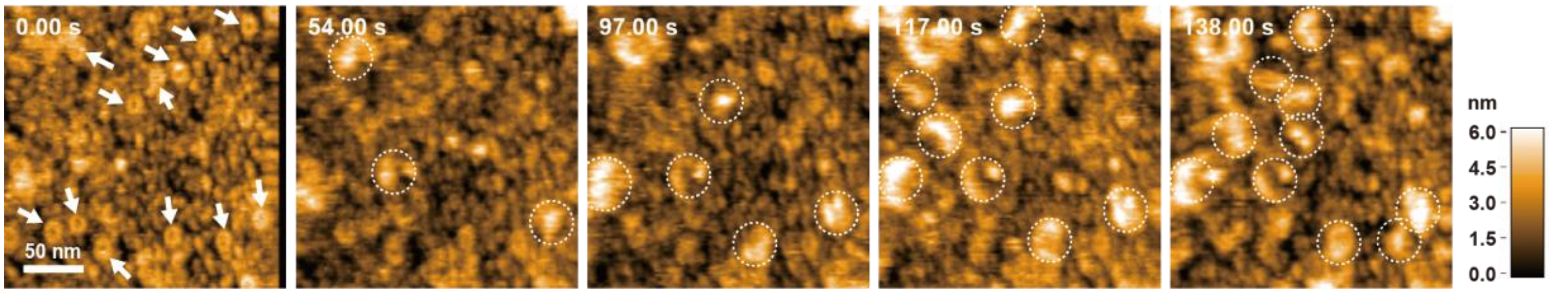

Supplementary Figure 4. Real-time visualization of CagM binding to CagX rings by HS-AFM. Wide-field time-lapse HS-AFM images showing the assembly of CagM (142 nM) onto immobilized CagX rings on APTES-mica. White arrows indicate CagX rings and dotted circles highlight CagM binding onto CagX rings. Images were acquired in continuous scanning mode at 1 s/frame. Scale bar, 50 nm.

a

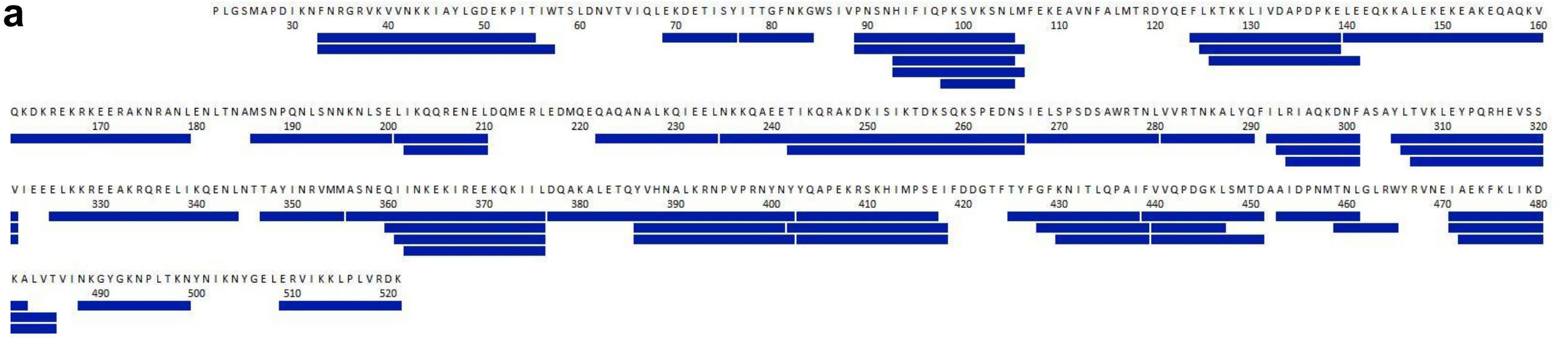

52 peptides, 81.4% coverage, 1.91 redundancy

b

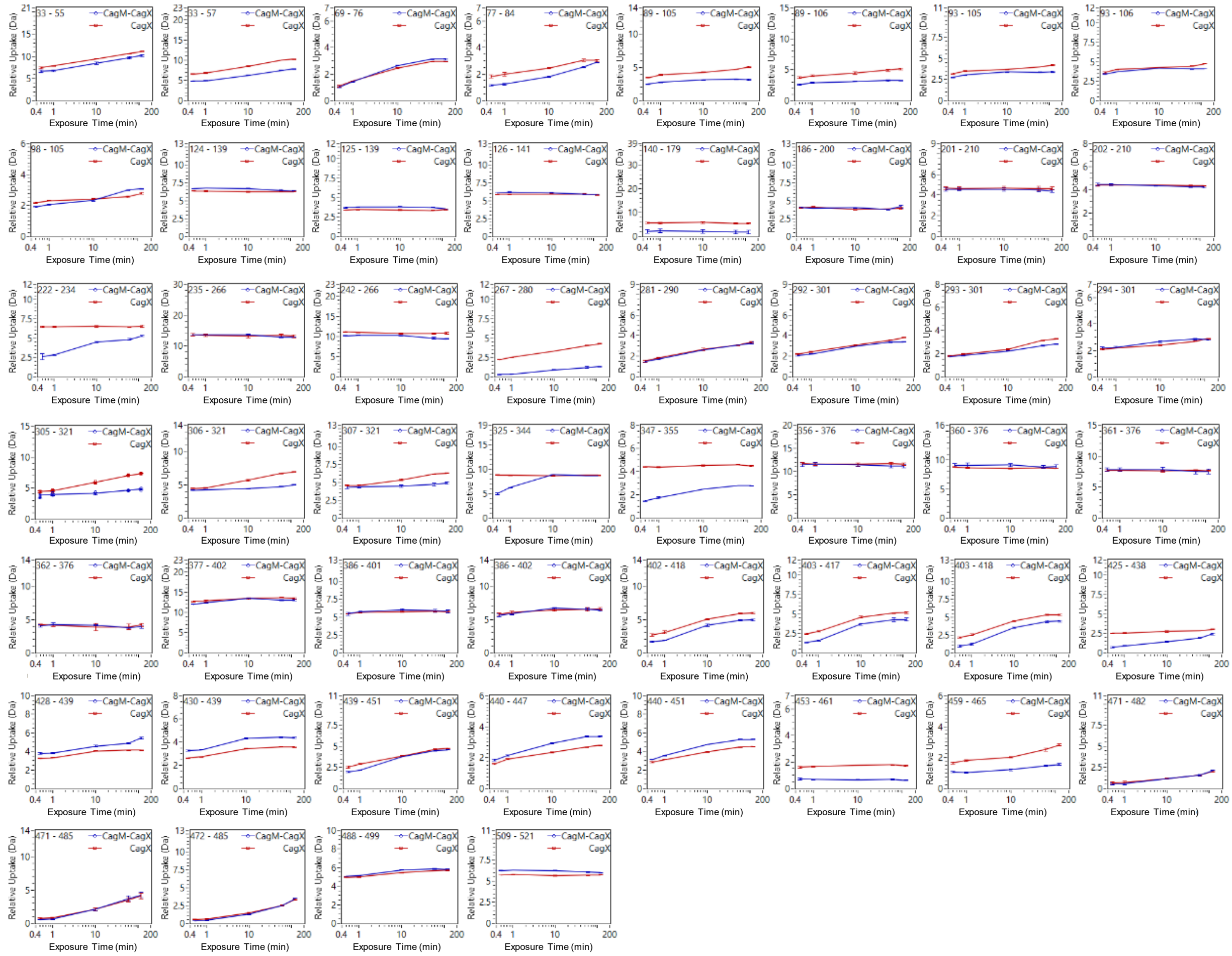

Supplementary Figure 5. HDX-MS of CagX alone and in complex with CagM. a Sequence coverage of CagX. **b** HDX-MS time courses for all peptides analyzed in apo- and complex states. Error bars indicate standard deviations for each time point (n = 3).

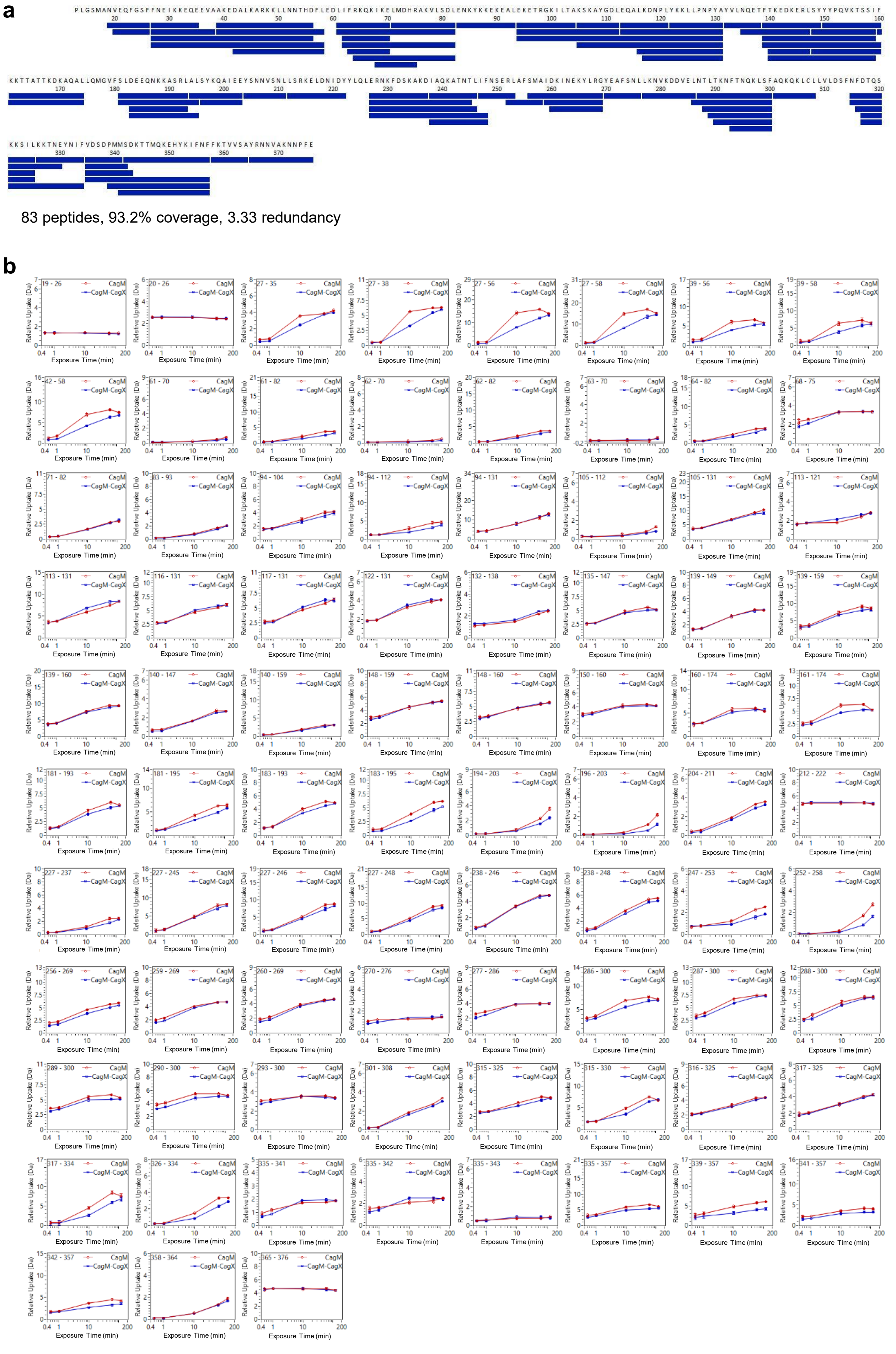

Supplementary Figure 6. HDX-MS of CagM alone and in complex with CagX. a Sequence coverage of CagM. **b** HDX-MS time courses for all peptides analyzed in apo- and complex states. Error bars indicate standard deviations for each time point (n = 3).

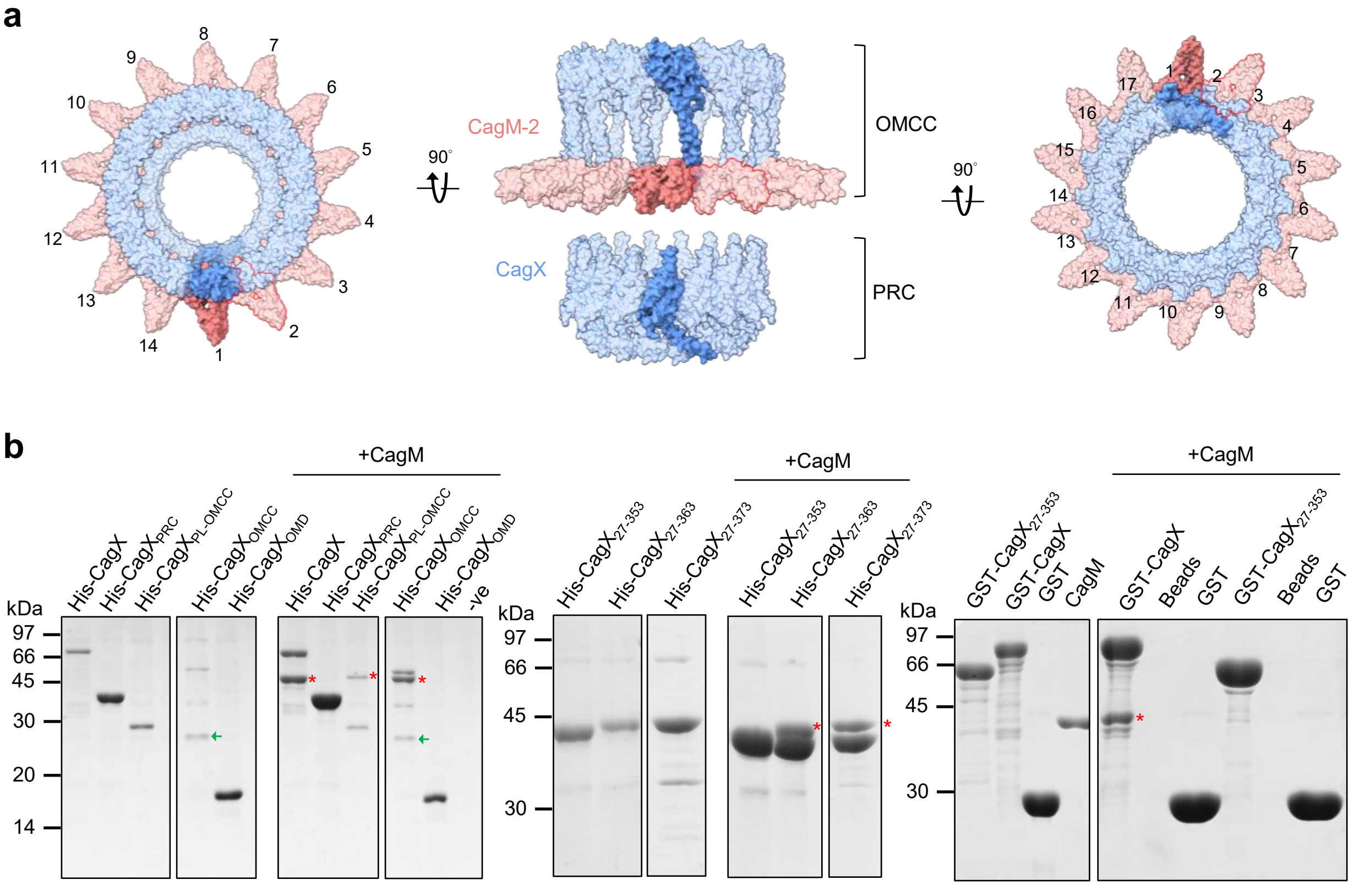

Supplementary Figure 7. Interaction interface of CagM-CagX complex. a Partially resolved cryo-EM model in previous study (PDB ID: 6X6S and 6X6J) showing CagX interacts with CagM-2 in neighbouring ASU (outlined in red) instead of within same ASU (non-transparent), and the C14:C17 symmetry mismatch between OMCC and PRC. **b** Nickel-NTA pull-down assays using His-CagX and its truncated constructs against purified CagM (left and middle). Arrows indicate band of His-CagX$_{OMCC}$ and the CagM band is indicated by asterisk. Full-length CagX was used as positive control and nickel sepharose was used as negative control. Pull-down assays using GST-CagX$_{27\text{-}353}$ as bait and purified CagM as prey with controls were set (right). CagM band is indicated by asterisk. The additional GST tag provides a distinguishable molecular size difference between them.

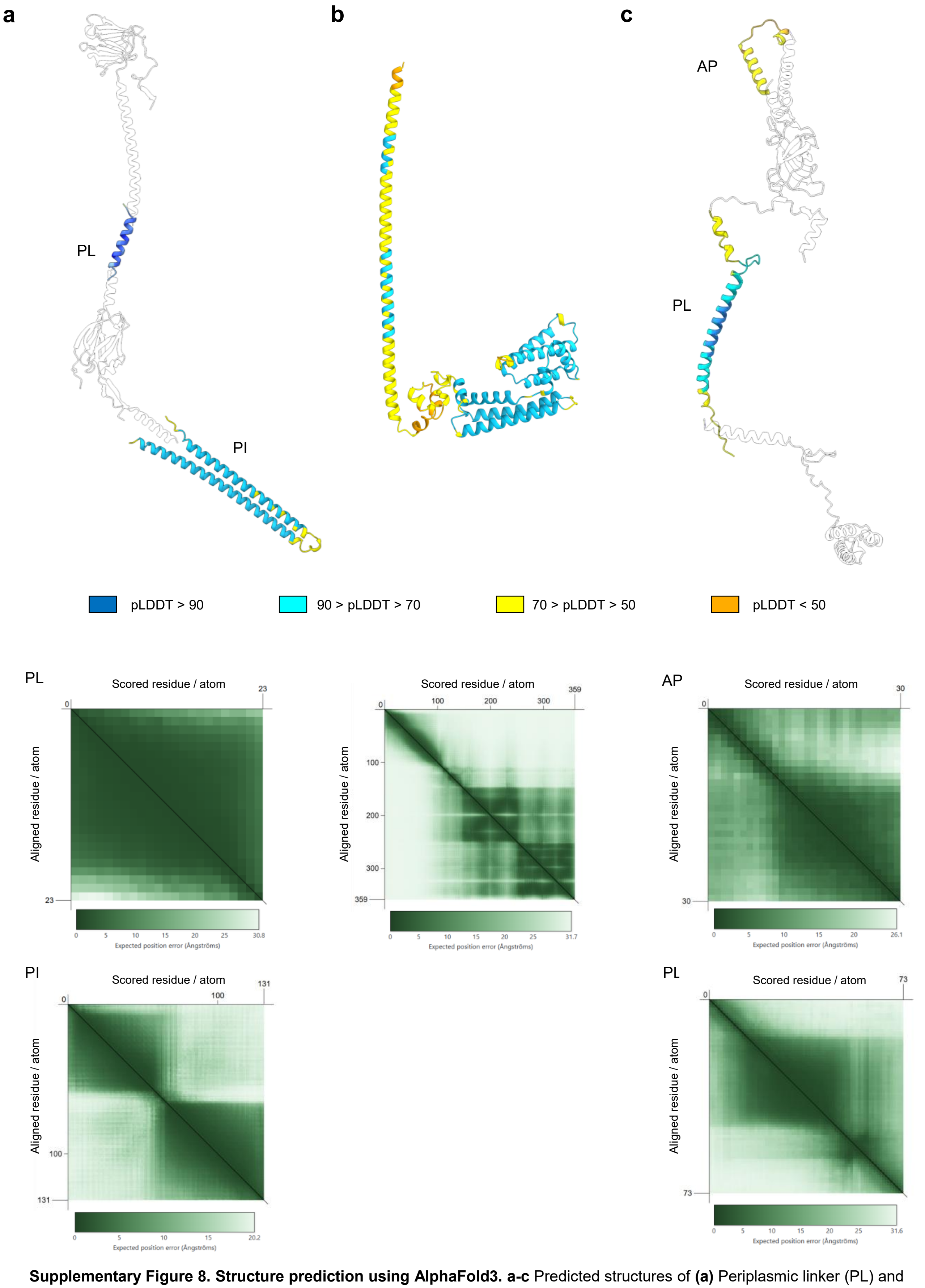

Supplementary Figure 8. Structure prediction using AlphaFold3. a-c Predicted structures of **(a)** Periplasmic linker (PL) and periplasmic insertion (PI) regions of CagX, **(b)** CagM, and **(c)** antenna projection (AP) and PL regions of CagY are colored based on pLDDT scores. Respective predicted aligned error (PAE) are shown. CagX and CagY structures predicted were integrated with the previously resolved cryo-EM structure (transparent) for modelling.

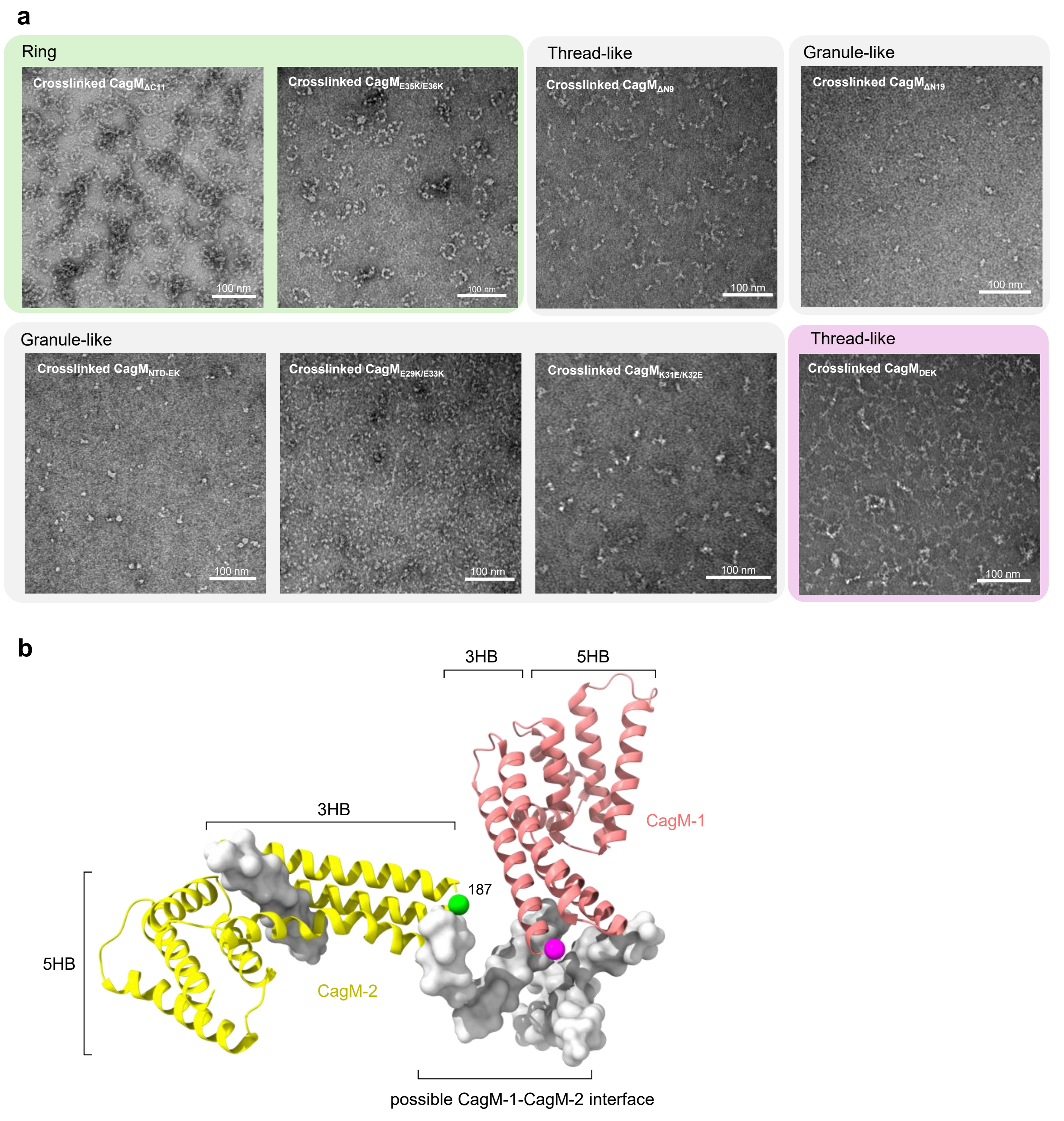

Supplementary Figure 9. Effects of different CagM mutants on ring formation. a Representative negative stain EM micrographs of different mutated CagM constructs after DSSO-crosslinking, grouped by their oligomeric states with the background coloured according to Fig. 3A. Distinct structural features were observed from these mutants, including ring, thread-like and granule-like particles. **b** A previously published OMCC cryo-EM model (PDB ID: 6X6S) showing the unresolved residues 183-185 of CagM-2 may occupy the unknown polyAla model (light grey surface) and lie close to CagM-1. The first resolved residue (residue 187) of both CagM-1 and CagM-2 is shown in sphere.

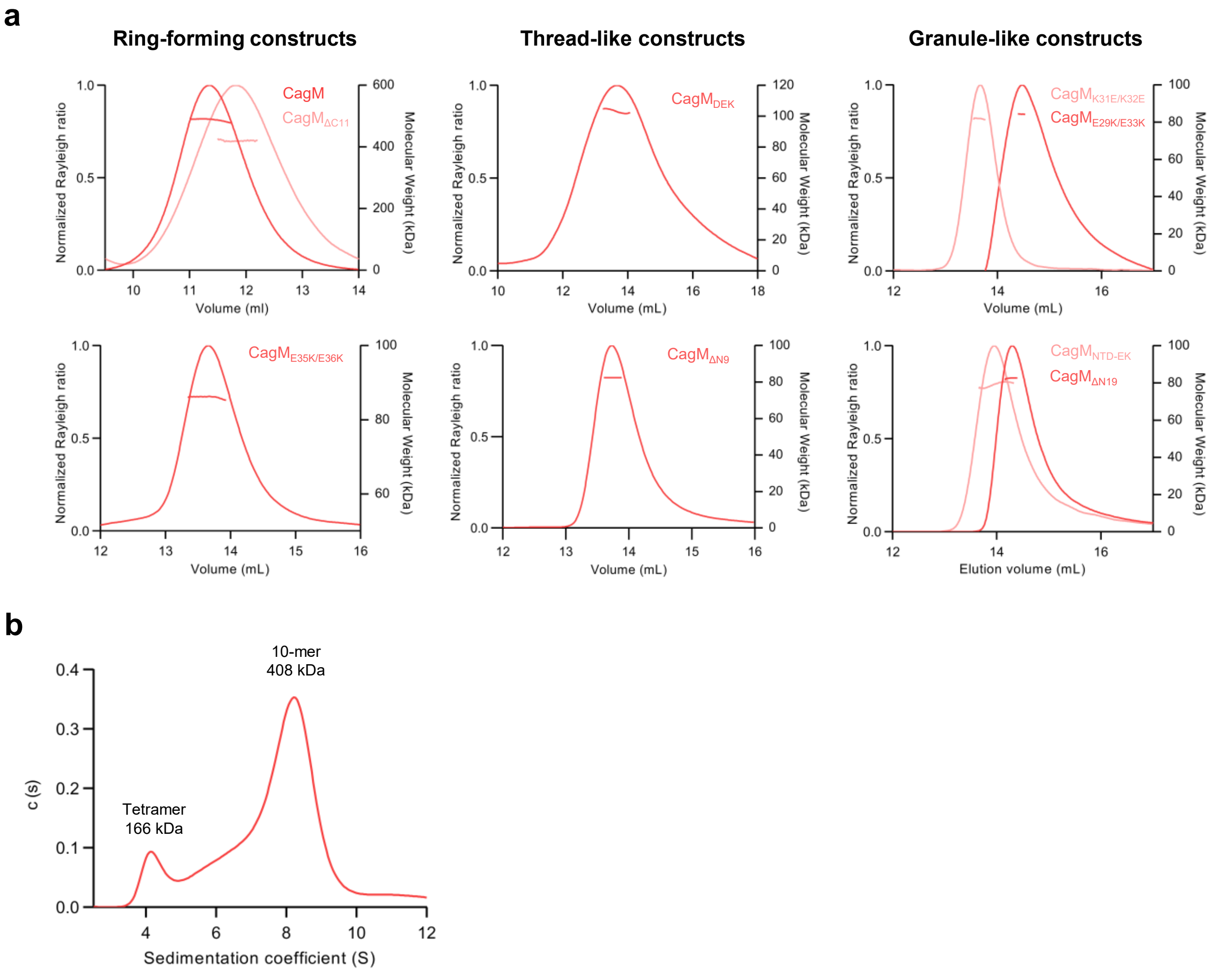

Supplementary Figure 10. CagM ring formation is dependent on its oligomeric state. a Oligomeric states of CagM mutants analyzed by SEC-SLS and grouped based on their ring-forming capabilities under crosslinking condition. **b** AUC analysis of $CagM_{E35K/E36K}$ showing it mainly exists as high-order oligomer.

a

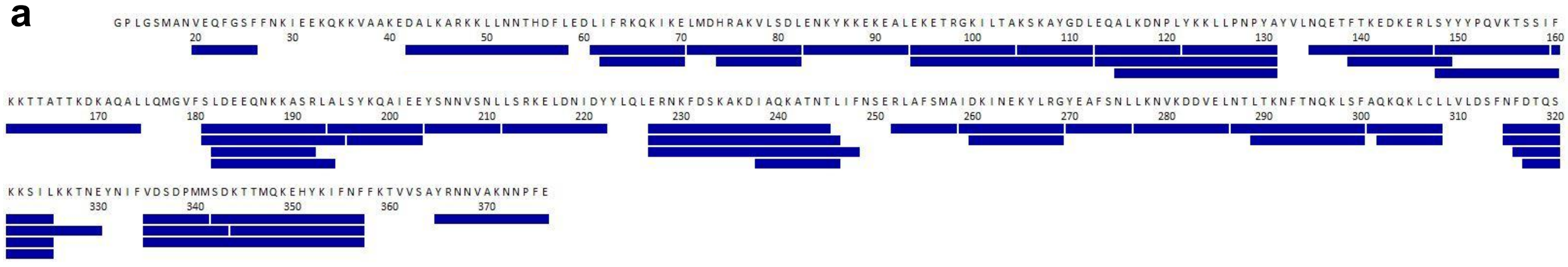

50 peptides, 84.1% coverage, 1.97 redundancy

b

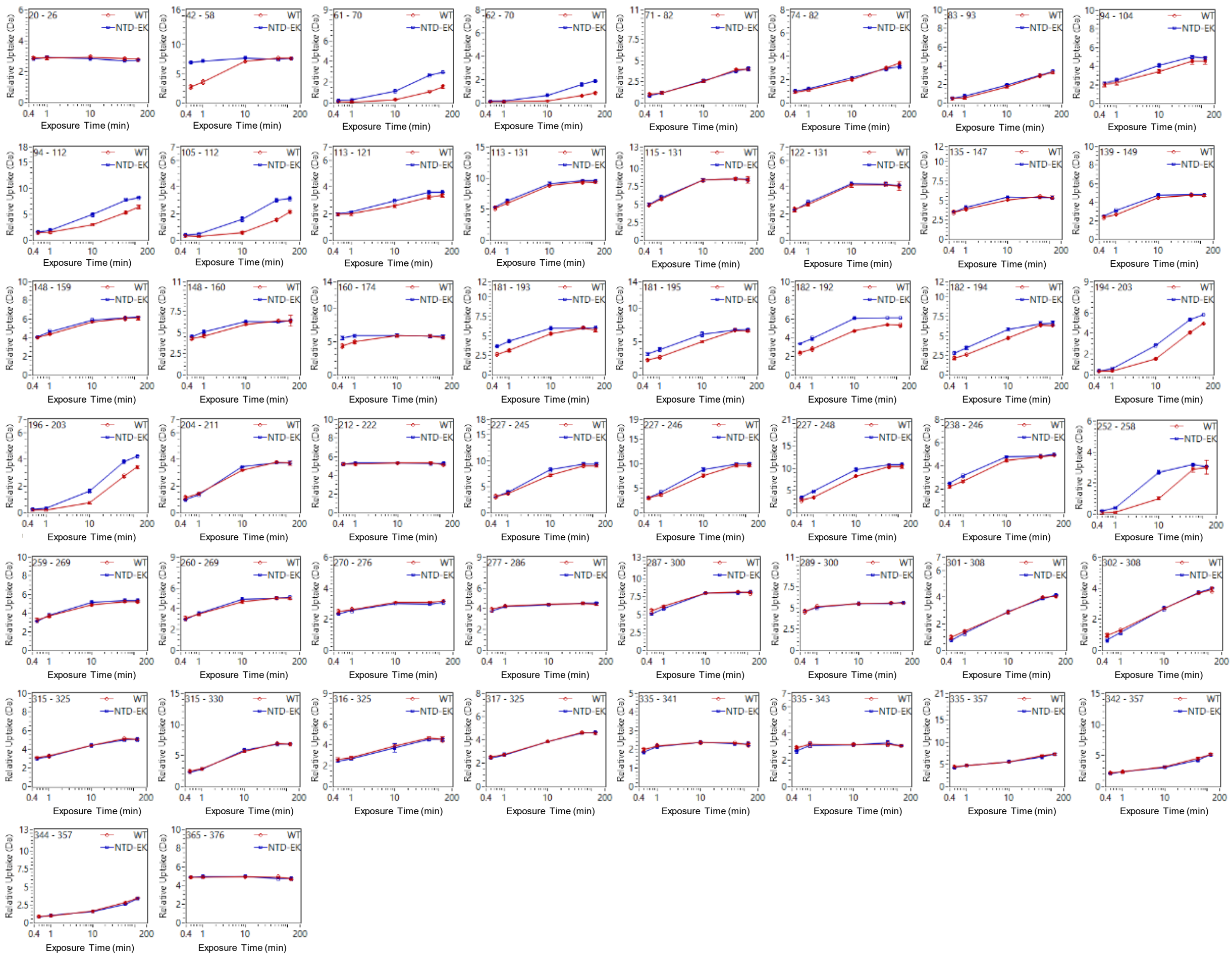

Supplementary Figure 11. HDX-MS of CagM$_{NTD-EK}$ mutant. a Sequence coverage. **b** HDX-MS time courses for all peptides analyzed in CagM wild-type and CagM$_{NTD-EK}$ mutant. Error bars indicate standard deviations for each time point (n = 3).

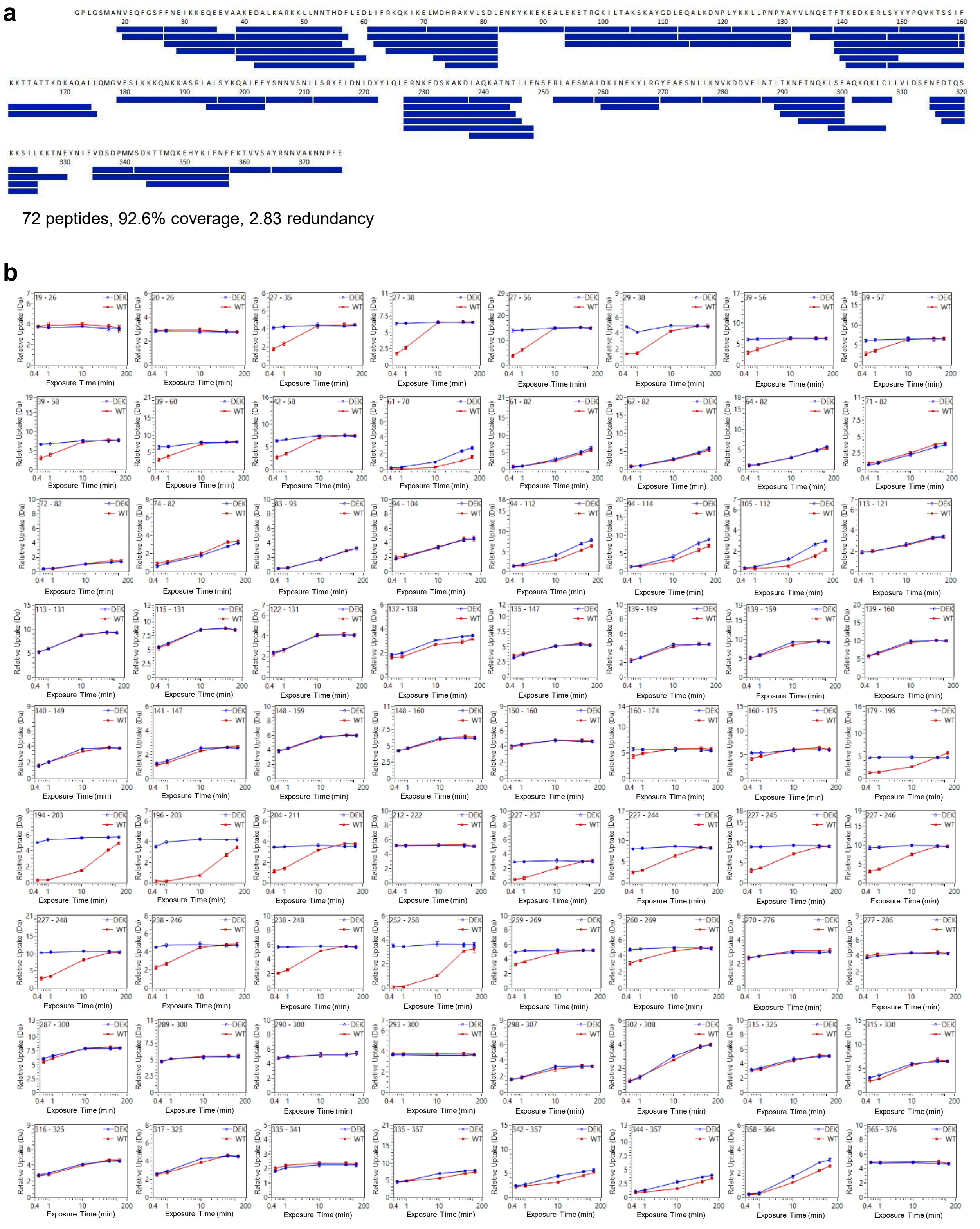

Supplementary Figure 12. HDX-MS of CagM$_{DEK}$ mutant. a Sequence coverage. **b** HDX-MS time courses for all peptides analyzed in CagM wild-type and CagM$_{DEK}$ mutant. Error bars indicate standard deviations for each time point (n = 3).

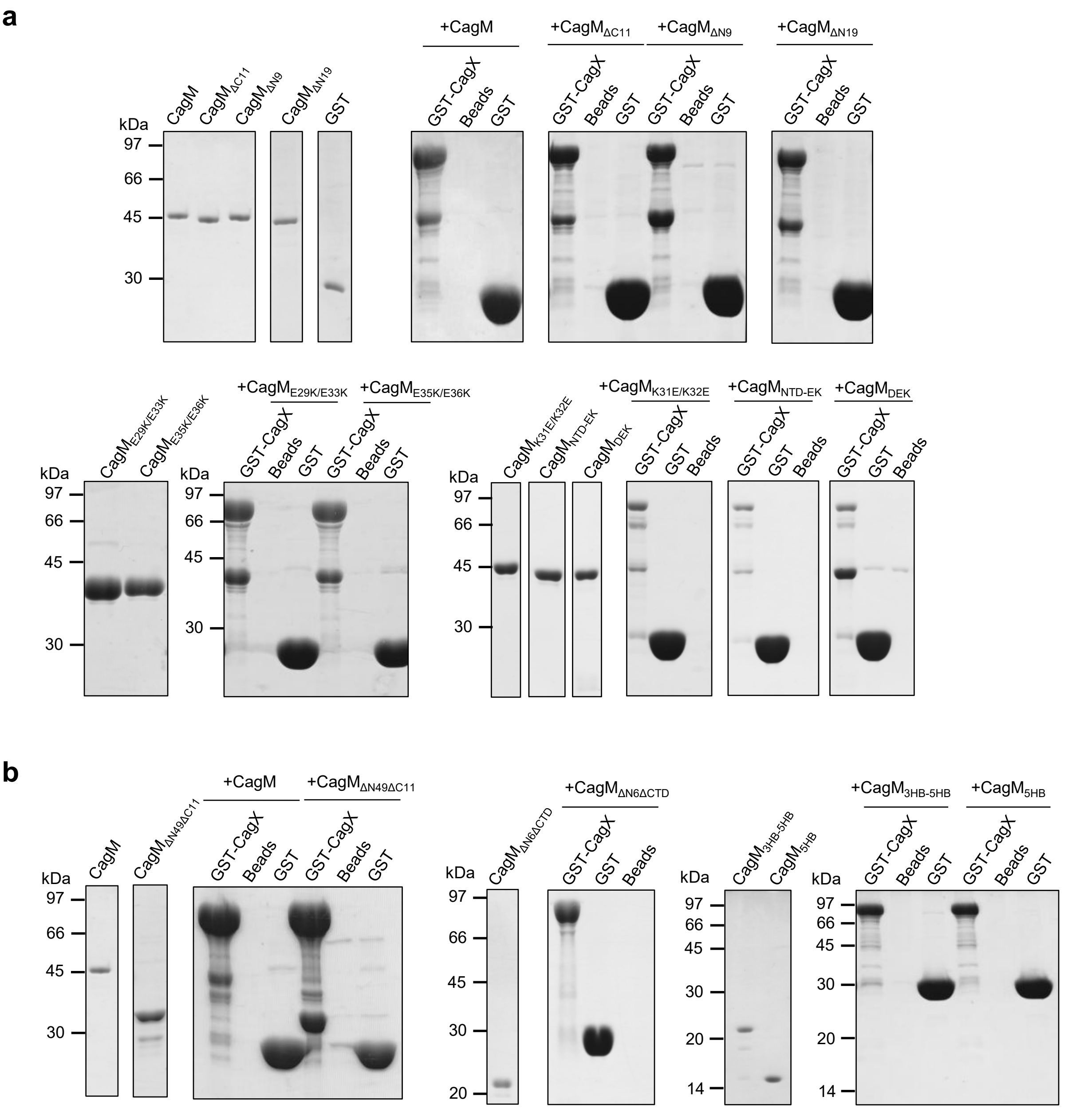

Supplementary Figure 13. Systematic pulldown assays defining the interaction interface of CagM-CagX complex. a-b Pull-down assays using GST-CagX as bait and purified (**a**) CagM mutants or **(b)** specifically designed CagM truncations as prey. Wild-type CagM was used as positive control while CagM mutants to GST proteins and glutathione sepharose were used as negative controls.

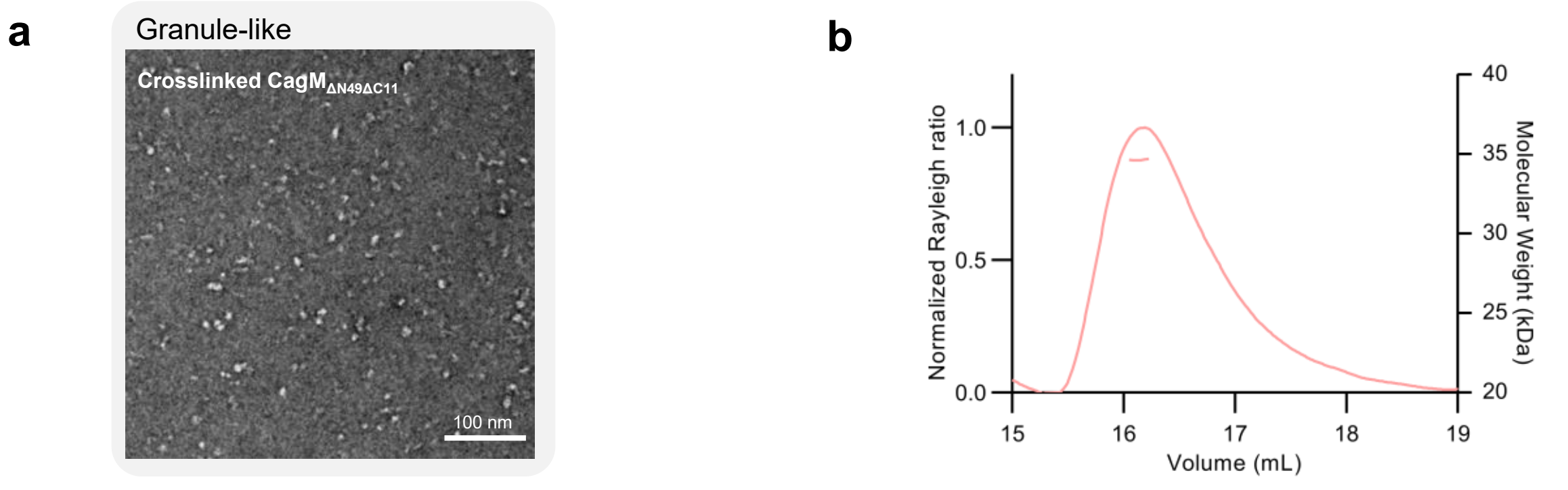

Supplementary Figure 14. Characterization of CagM$_{\Delta N49\Delta C11}$. **a** Representative negative stain EM micrographs of CagM$_{\Delta N49\Delta C11}$ after DSSO-crosslinking showing granule-like particles as CagM$_{\Delta N19}$. **b** SEC-SLS of CagM$_{\Delta N49\Delta C11}$ revealing its monomeric state.

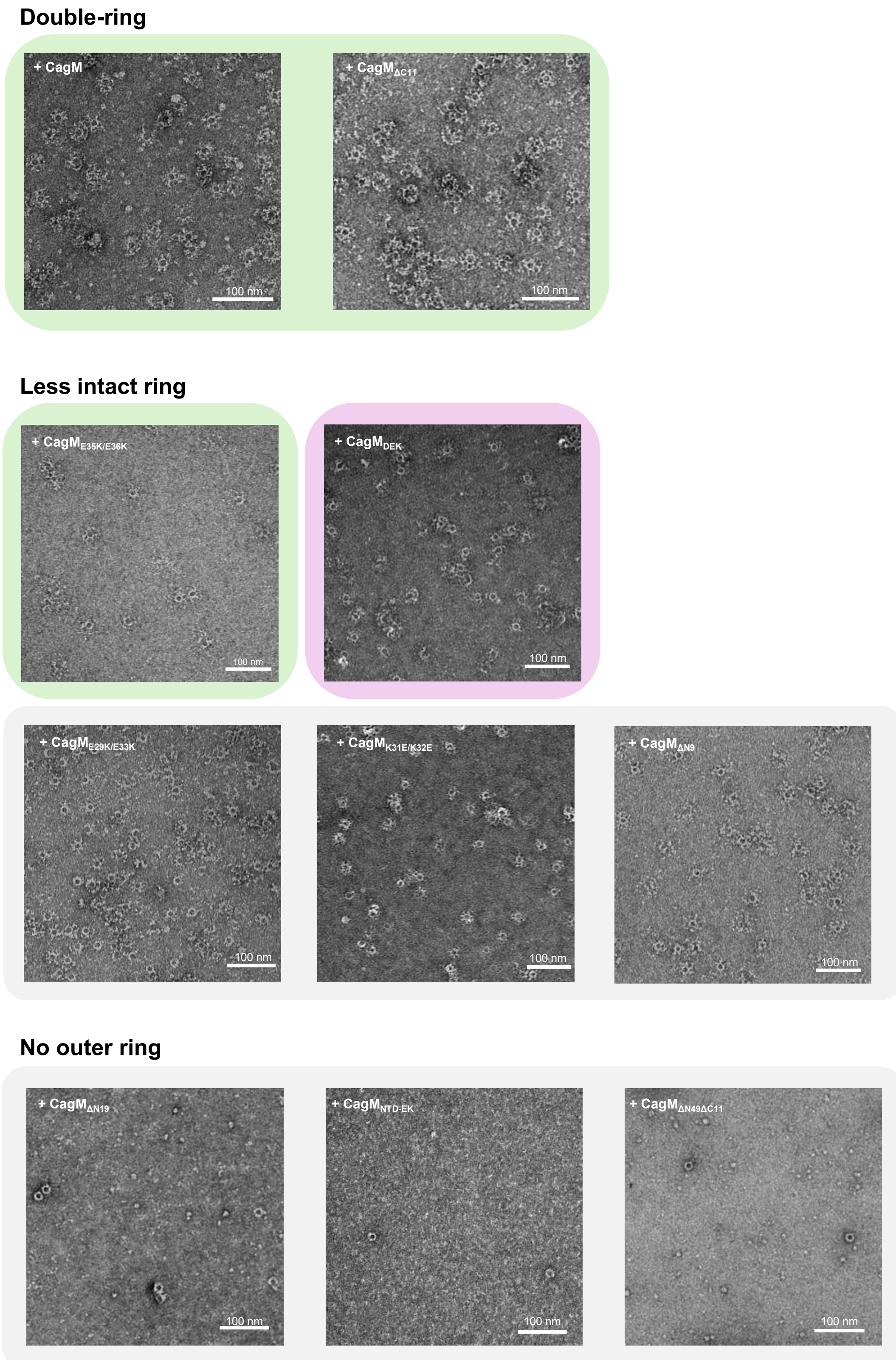

Supplementary Figure 15. Effects of mutated CagM on CagM-CagX complex double-ring formation. Representative negative stain EM images of different CagM mutants co-incubated with CagX followed by purification using SEC, grouped by their oligomeric states with the background coloured according to Fig. 3A. Only high-order oligomeric CagM forms intact double-ring complex with CagX, implying the importance of high-order homo-oligomerization in reconstituting the double-ring complex.

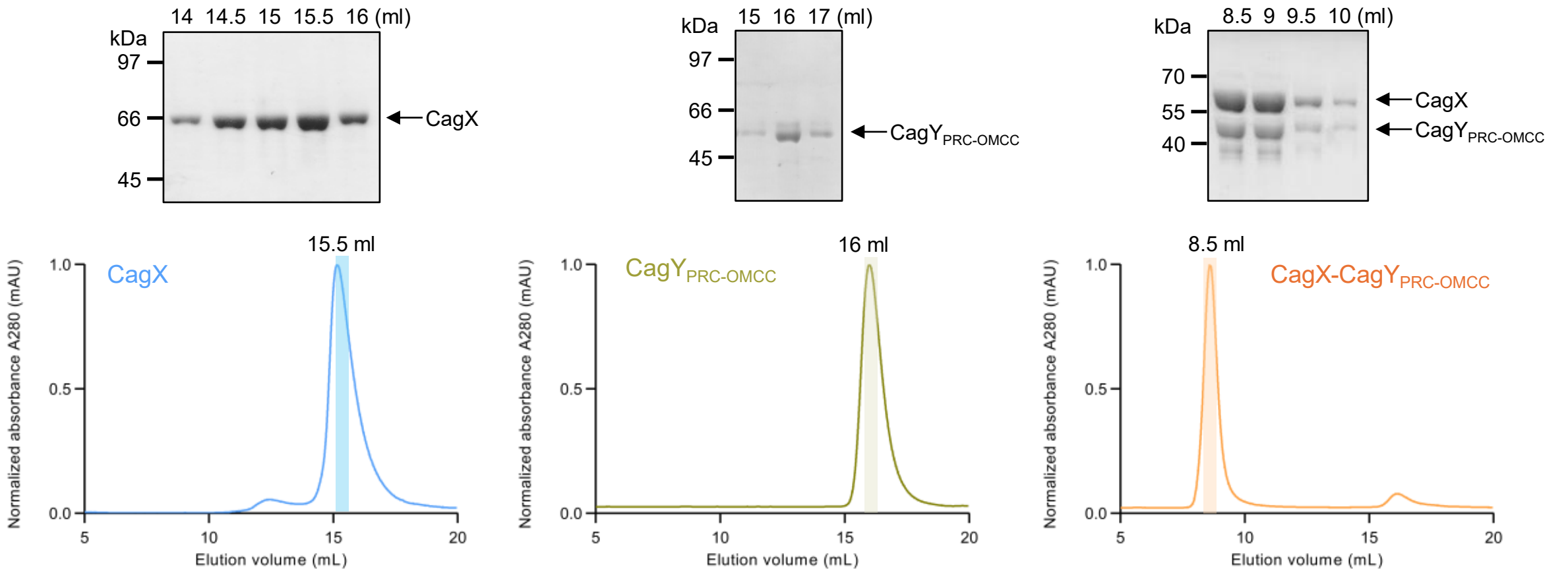

Supplementary Figure 16. Purification of CagX-CagY$_{\mathbf{PRC\text{-}OMCC}}$ complex. Elution profiles and SDS-PAGE analysis of purified CagX, CagY$_{PRC\text{-}OMCC}$ and co-incubated CagX-CagY$_{PRC\text{-}OMCC}$ complex after Superose 6 column.

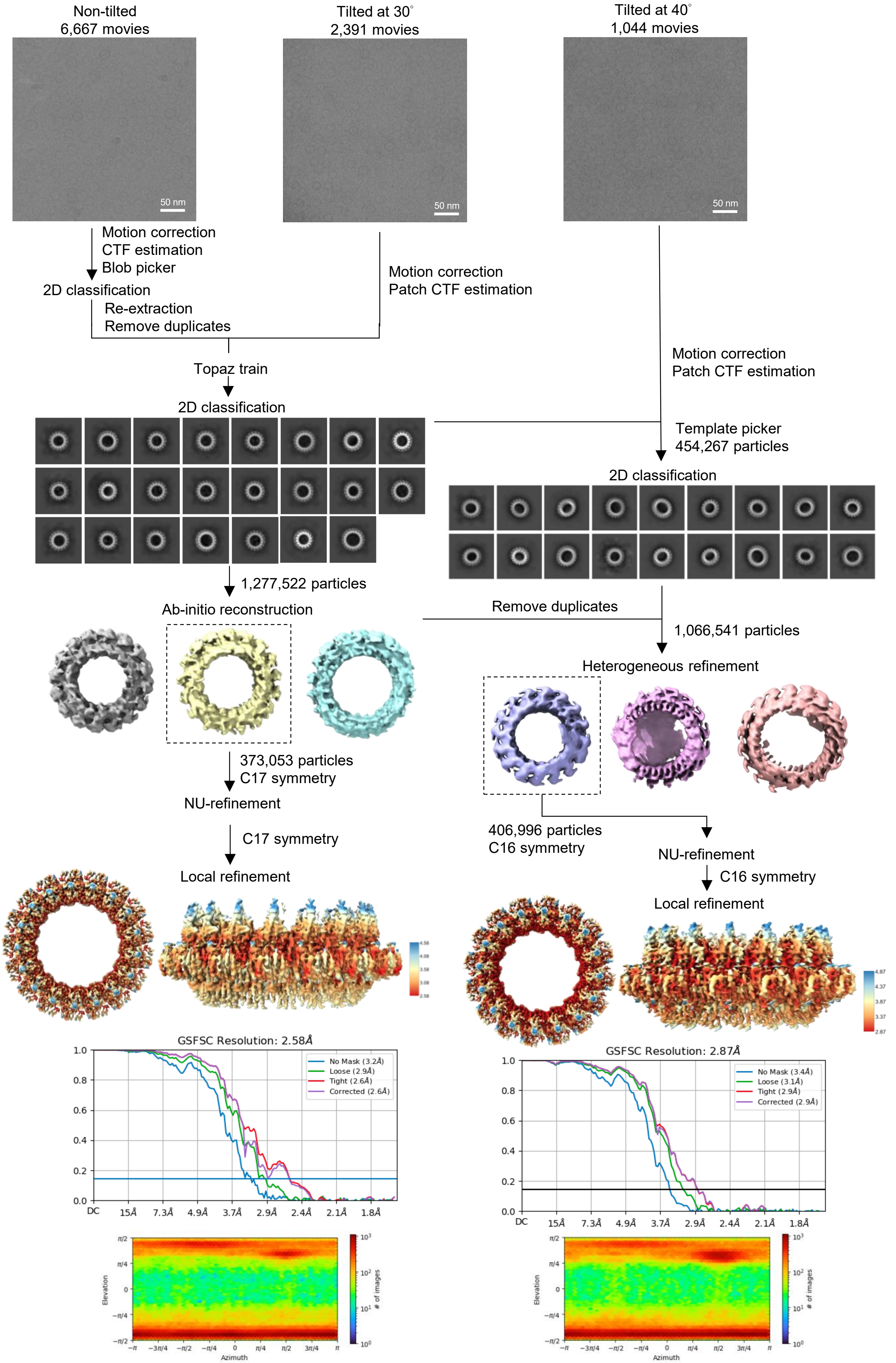

Supplementary Figure 17. Single particle cryo-EM processing workflow of CagX-CagY$_{PRC\text{-}OMCC}$ complex. The resolutions of the final C16 and C17 maps were determined according to Fourier shell correlation (FSC) with a criterion of 0.143. Respective 2D angular distributions are shown. Cryo-EM maps are colored by local resolution.

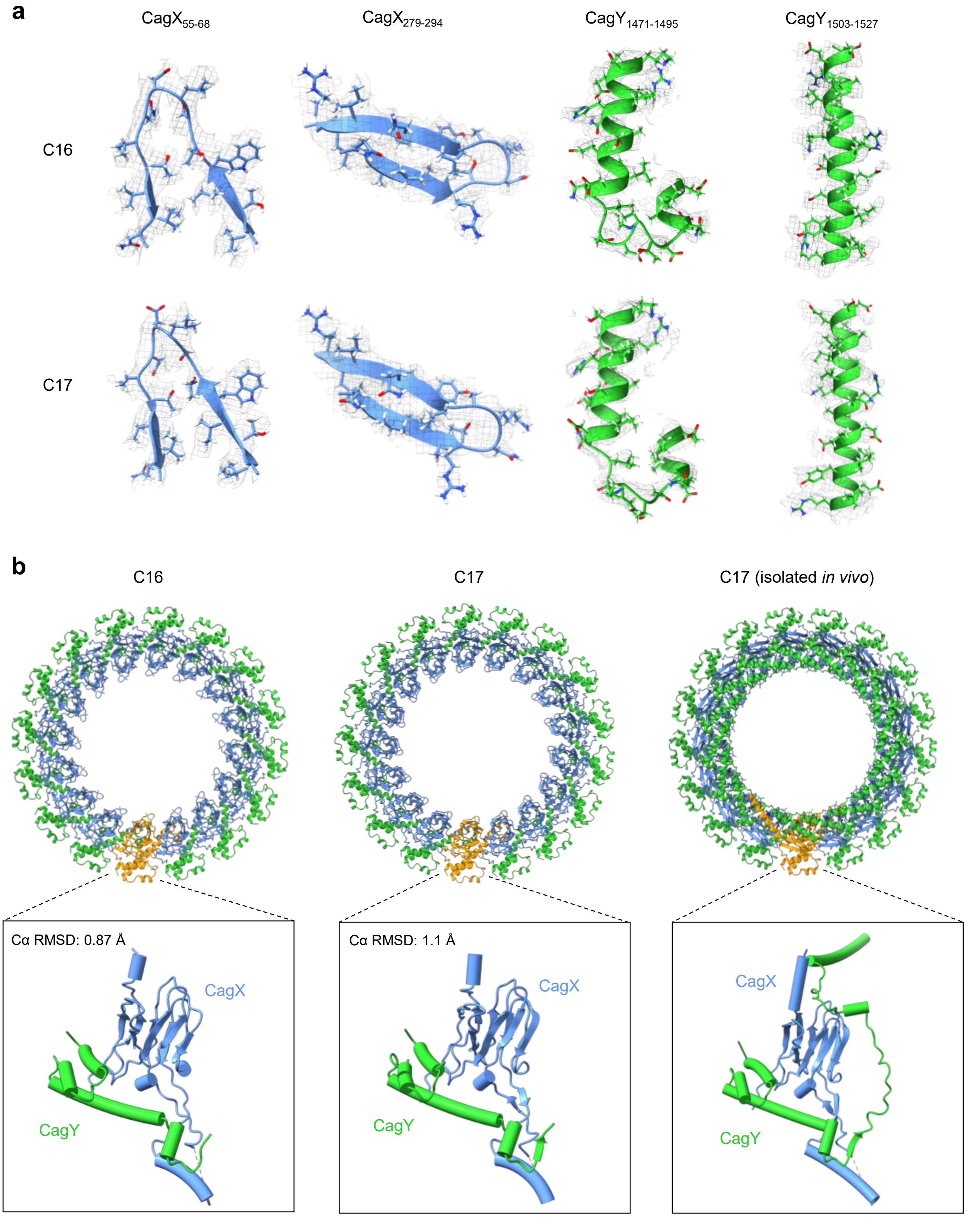

Supplementary Figure 18. Cryo-EM structures of CagX-CagY$_{PRC\text{-}OMCC}$ complex in C16 and C17 symmetries. a Representative densities with atomic models of C16 and C17 structures. **b** Cryo-EM structures of CagX-CagY$_{PRC\text{-}OMCC}$ complex in C16 and C17 symmetries in comparison with the PRC model isolated *in vivo* (PDB ID: 6X6J). One ASU is highlighted in orange and the arrangements of CagX and CagY within the ASU are shown in zoom-in box. The Cα RMSD between our *in vitro*-isolated complexed and *in vivo*-isolated PRC are shown.

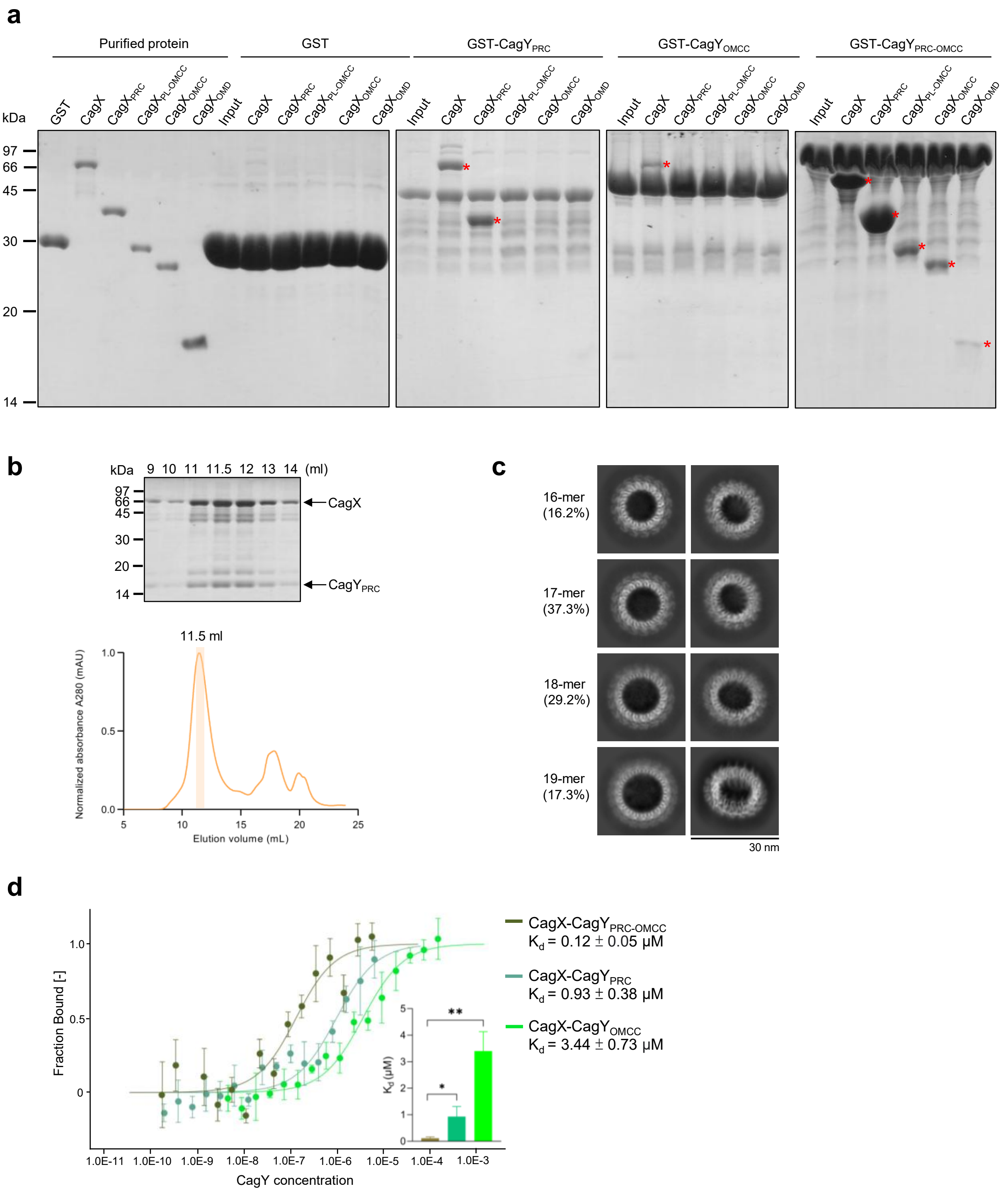

Supplementary Figure 19. Characterization of CagX-CagY complex. a Pull-down assays demonstrating the major interaction interface of CagX-CagY complex lies on PRC. Glutathione sepharose pull-down using GST-CagY$_{PRC}$, GST-CagY$_{OMCC}$ and GST-CagY$_{PRC-OMCC}$ were performed against different CagX truncations. Bands of CagX truncations are indicated as asterisks. Negative controls using CagX truncations to GST protein to glutathione sepharose were set (left). Inputs were run in parallel before the addition of purified CagX proteins. **b** Elution profile and SDS-PAGE analysis of co-expressed and co-purified CagX-CagY$_{PRC}$ complex after Superose 6 column. **c** Representative cryo-EM 2D class averages of CagX-CagY$_{PRC}$ complex showing similar structural features and oligomeric states as CagX-CagY$_{PRC-OMCC}$ complex. **d** Microscale thermophoresis assays showing the binding affinities between CagX and different CagY constructs. Statistical K_d differences are indicated in bar chart, calculated using Student's t-test (** $p < 0.01$ and * $p < 0.05$).

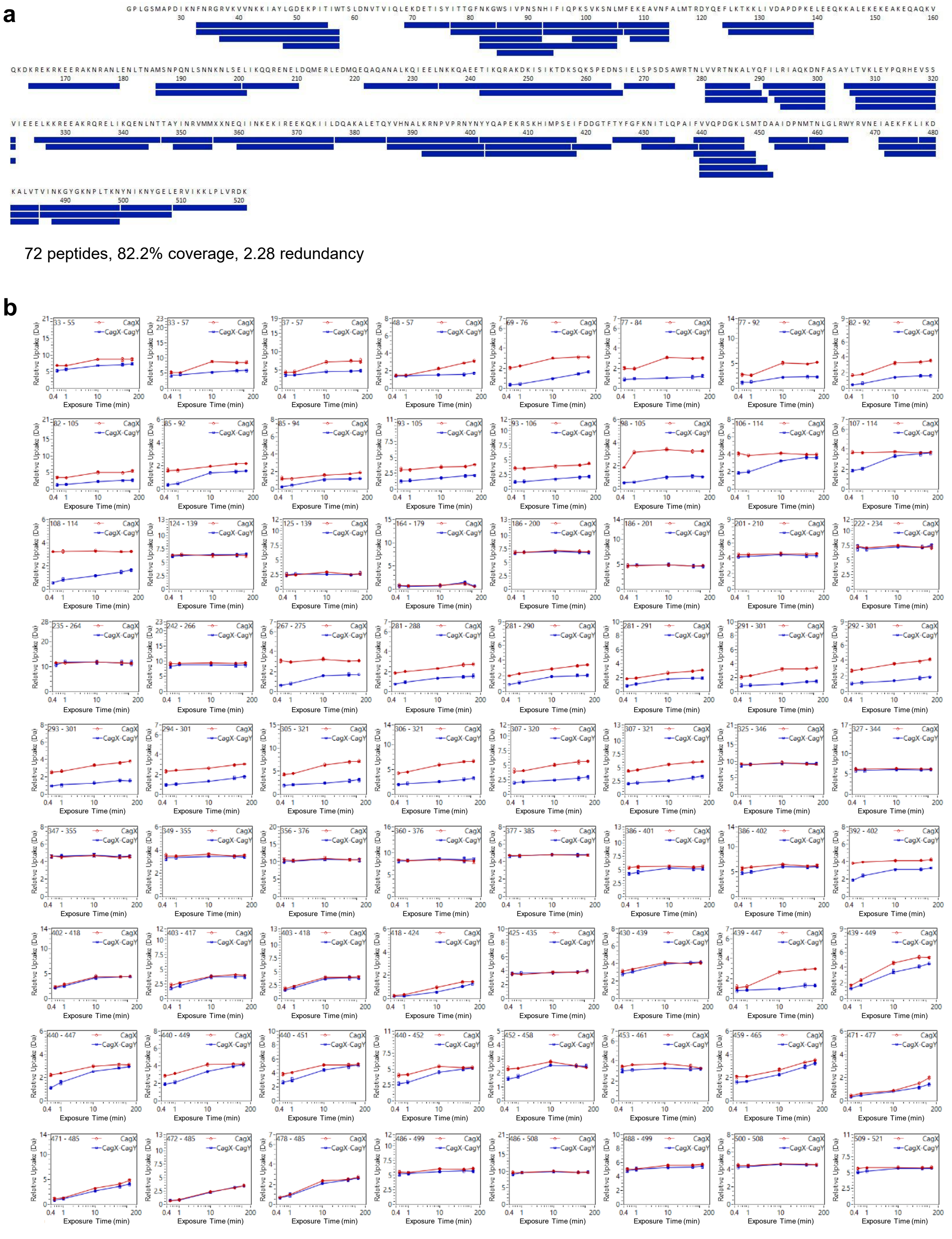

Supplementary Figure 20. HDX-MS of CagX alone and in complex with CagY$_{PRC-OMCC}$. a Sequence coverage of CagX. **b** HDX-MS time courses for all peptides analyzed in apo- and complex states. Error bars indicate standard deviations for each time point (n = 3).

a

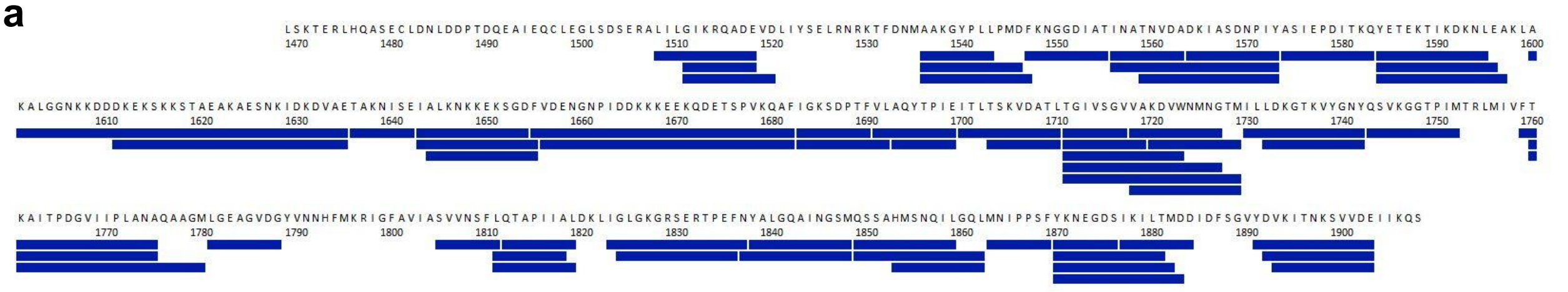

64 peptides, 79.1% coverage, 2.27 redundancy

b

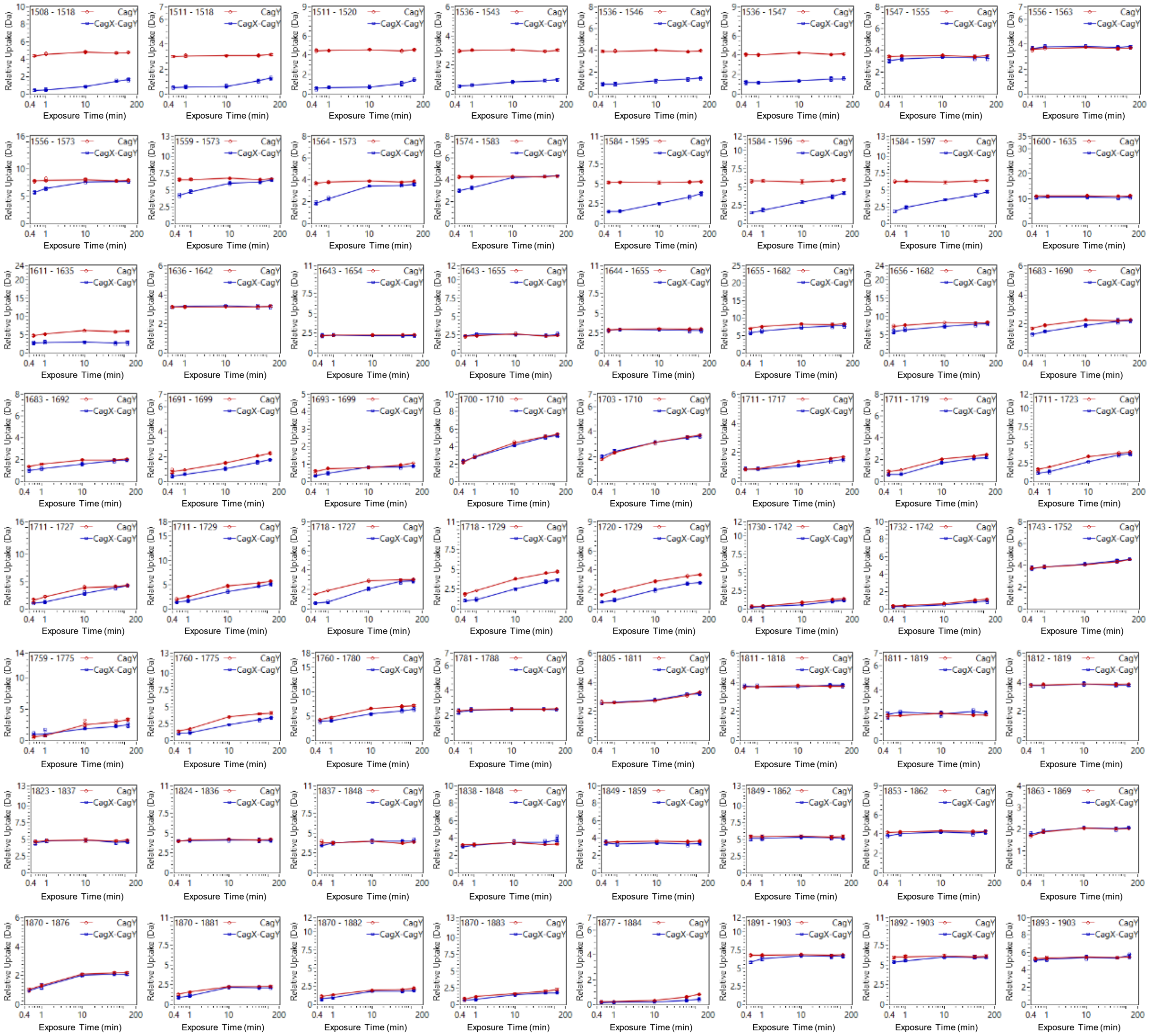

Supplementary Figure 21. HDX-MS of CagY$_{PRC\text{-}OMCC}$ alone and in complex with CagX. a Sequence coverage of CagY$_{PRC\text{-}OMCC}$. **b** HDX-MS time courses for all peptides analyzed in apo- and complex states. Error bars indicate standard deviations for each time point (n = 3).

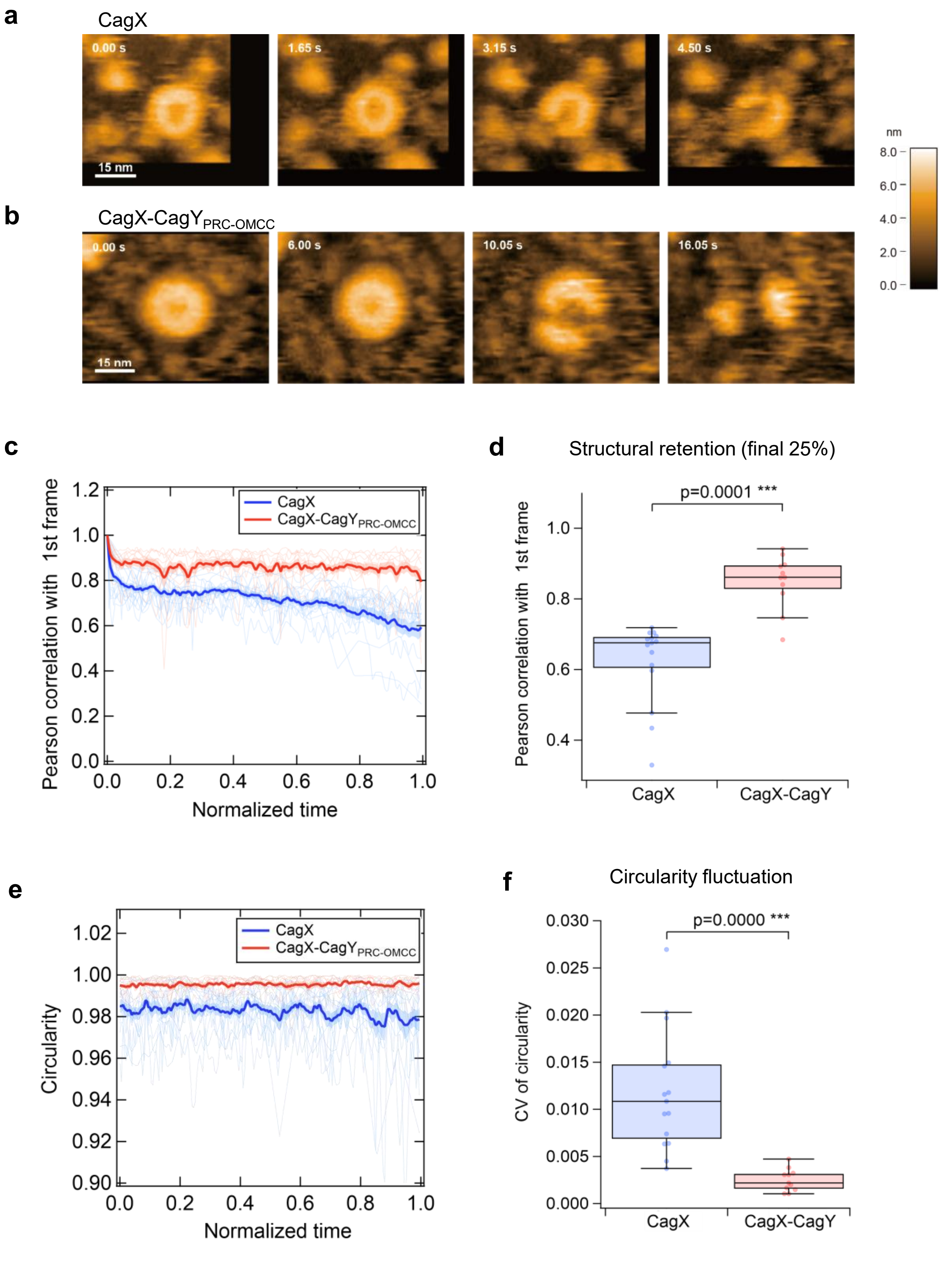

Supplementary Figure 22. Dynamic stability comparison of CagX and CagX-CagY$_{PRC-OMCC}$rings under continuous HS-AFM scanning. a-b Time-lapse HS-AFM images showing the progressive disassembly of **(a)** a CagX ring on APTES-treated mica and **(b)** a CagX-CagY$_{PRC-OMCC}$ ring on bare mica during continuous scanning at 0.15 s/frame. The repeated tip–sample interaction gradually disrupts the ring structure over successive frames. Scale bars, 15 nm. **c** Time evolution of the Pearson correlation coefficient between each frame and the first frame for CagX (blue; n = 15) and CagX-CagY$_{PRC-OMCC}$ (red; n = 11) rings. The time axis is normalized to the total observation period of each molecule. Thin lines represent individual molecules and bold lines represent the mean. The progressive decline in both traces reflects the gradual structural disruption during scanning, with the CagX-CagY$_{PRC-OMCC}$ ring maintaining structural integrity over a greater number of frames. **d** Structural retention rate, defined as the fraction of frames maintaining a Pearson correlation coefficient over the final 25% of frames. CagX-CagY$_{PRC-OMCC}$ rings exhibited significantly higher structural retention than CagX rings alone (median: 0.86 vs. 0.68; p = 8.0 × 10^{-5}, Mann–Whitney U test; n = 11 and 15, respectively). **e** Time evolution of circularity for CagX (blue; n = 15) and CagX-CagY$_{PRC-OMCC}$ (red; n = 11) rings. The time axis is normalized as in **(c)**. Thin lines represent individual molecules and bold lines represent the mean. CagX rings exhibit larger temporal fluctuations in circularity compared to CagX-CagY$_{PRC-OMCC}$ rings. **f** Coefficient of variation of circularity for CagX and CagX-CagY$_{PRC-OMCC}$ rings. CagX-CagY$_{PRC-OMCC}$ rings showed significantly lower circularity fluctuation than CagX rings (median: 0.0022 vs. 0.0108; p = 4.1 × 10^{-5}, Mann–Whitney U test), indicating that CagY binding confers mechanical stability to the ring architecture.

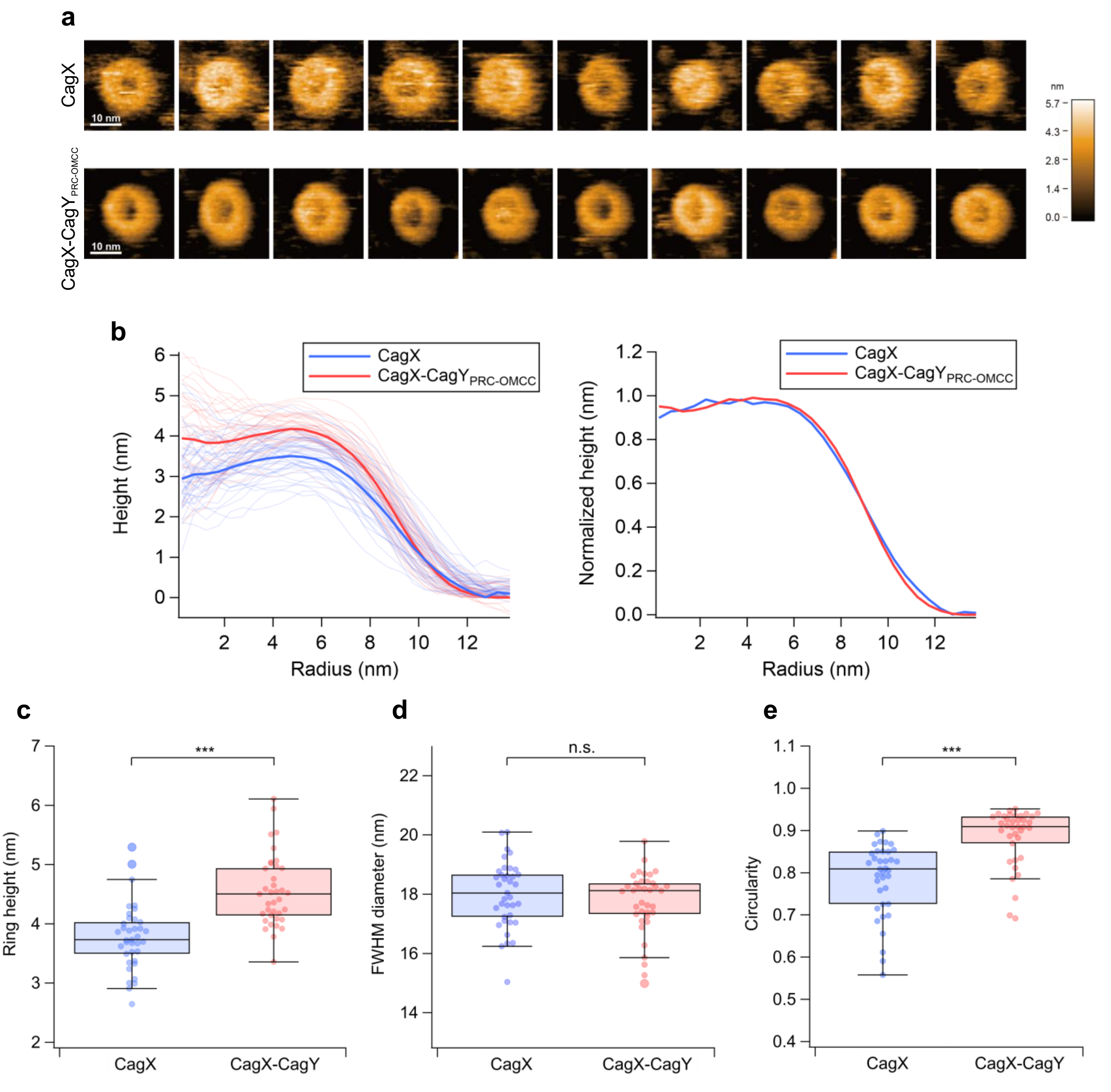

Supplementary Figure 23. Structural comparison of CagX and CagX-CagY$_{PRC\text{-}OMCC}$ rings by HS-AFM. a Representative HS-AFM images of CagX (upper) and CagX-CagY$_{PRC\text{-}OMCC}$ (lower) rings, each showing 10 individual molecules. Both datasets were acquired consecutively using the same AFM tip, ensuring that tip geometry effects are minimal for comparison. Scale bars, 10 nm. **b** Radial height profiles of CagX (blue; n = 45) and CagX-CagY$_{PRC\text{-}OMCC}$ (red; n = 39) rings. Thin lines represent individual molecules and bold lines represent the mean. Left: absolute height; right: normalized height (0–1). The normalized profiles are nearly superimposable, indicating that the overall ring shape is indistinguishable between CagX and CagX-CagY$_{PRC\text{-}OMCC}$ by AFM. **c** Ring height comparison. CagX-CagY$_{PRC\text{-}OMCC}$ rings are significantly taller than CagX rings (median: 4.10 vs. 3.68 nm; $p = 2.7 \times 10^{-9}$, Mann–Whitney U test), likely reflecting the greater structural rigidity of the CagX-CagY$_{PRC\text{-}OMCC}$ complex, which resists tip-induced deformation. **d** FWHM diameter comparison. No significant difference was observed between CagX and CagX-CagY$_{PRC\text{-}OMCC}$ rings (median: 17.5 nm for both; p = 0.077, Mann–Whitney U test), confirming that ring diameter alone cannot distinguish the two complexes. **e** Mean circularity comparison. CagX-CagY$_{PRC\text{-}OMCC}$ rings exhibited significantly higher circularity than CagX rings (median: 0.907 vs. 0.794; $p = 1.5 \times 10^{-7}$, Mann–Whitney U test), consistent with the greater structural stability conferred by CagY binding. Statistical significance: *** $p < 0.001$; n.s., not significant. Taken together, these results indicate that CagX and CagX-CagY$_{PRC\text{-}OMCC}$ rings cannot be distinguished by ring diameter in HS-AFM, but differences in ring height and circularity reflect the enhanced structural stability upon CagY binding.

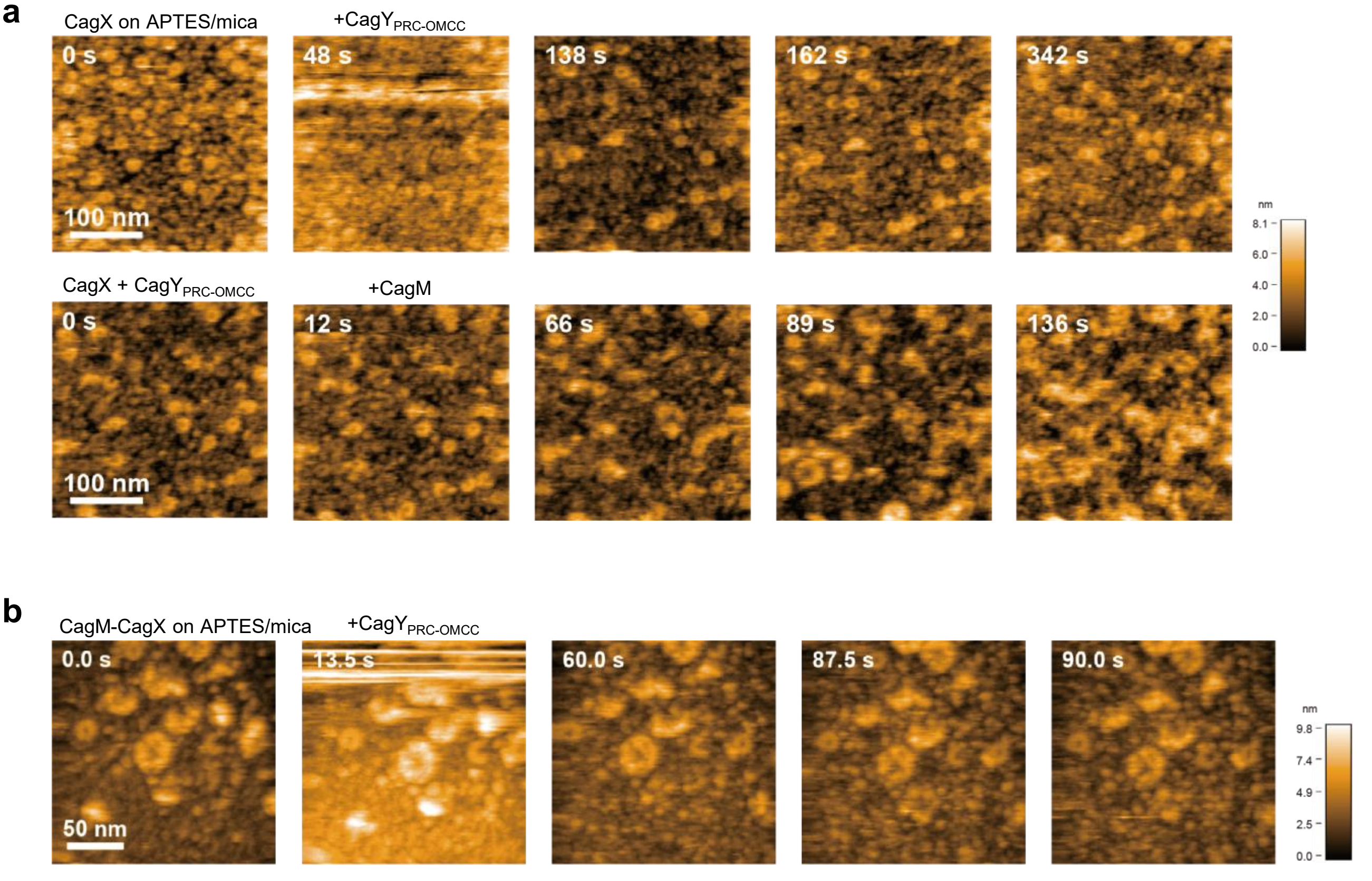

Supplementary Figure 24. Effect of assembly order on CagT4SS complex formation. a Sequential assembly in the canonical order. Upper row: time-lapse HS-AFM images of CagX rings immobilized on APTES-treated mica, followed by addition of $CagY_{PRC\text{-}OMCC}$. Images were acquired in intermittent scanning mode (1 frame on, 5 frames off) at 1 s/frame to minimize disruption of the mechanically fragile CagX rings. Lower row: subsequent addition of CagM to the CagX-$CagY_{PRC\text{-}OMCC}$ complexes, resulting in the formation of double-ring structures. Images were acquired in continuous scanning mode at 1 s/frame, as the CagX-$CagY_{PRC\text{-}OMCC}$ complex is sufficiently stable for continuous observation. Scale bars, 100 nm. **b** Reverse assembly order. Pre-formed CagM-CagX double-ring complexes were immobilized on APTES-treated mica, followed by addition of $CagY_{PRC\text{-}OMCC}$. No discernible structural change was observed in the CagM-CagX double rings upon $CagY_{PRC\text{-}OMCC}$ addition, indicating that CagY cannot incorporate into the pre-assembled CagM-CagX complex. Images were acquired in continuous scanning mode at 0.5 s/frame. Scale bar, 50 nm. These results demonstrate that CagT4SS core complex assembly follows a strict sequential order-CagX, then CagY, then CagM- and that CagY binding requires access to the CagX ring prior to CagM association.

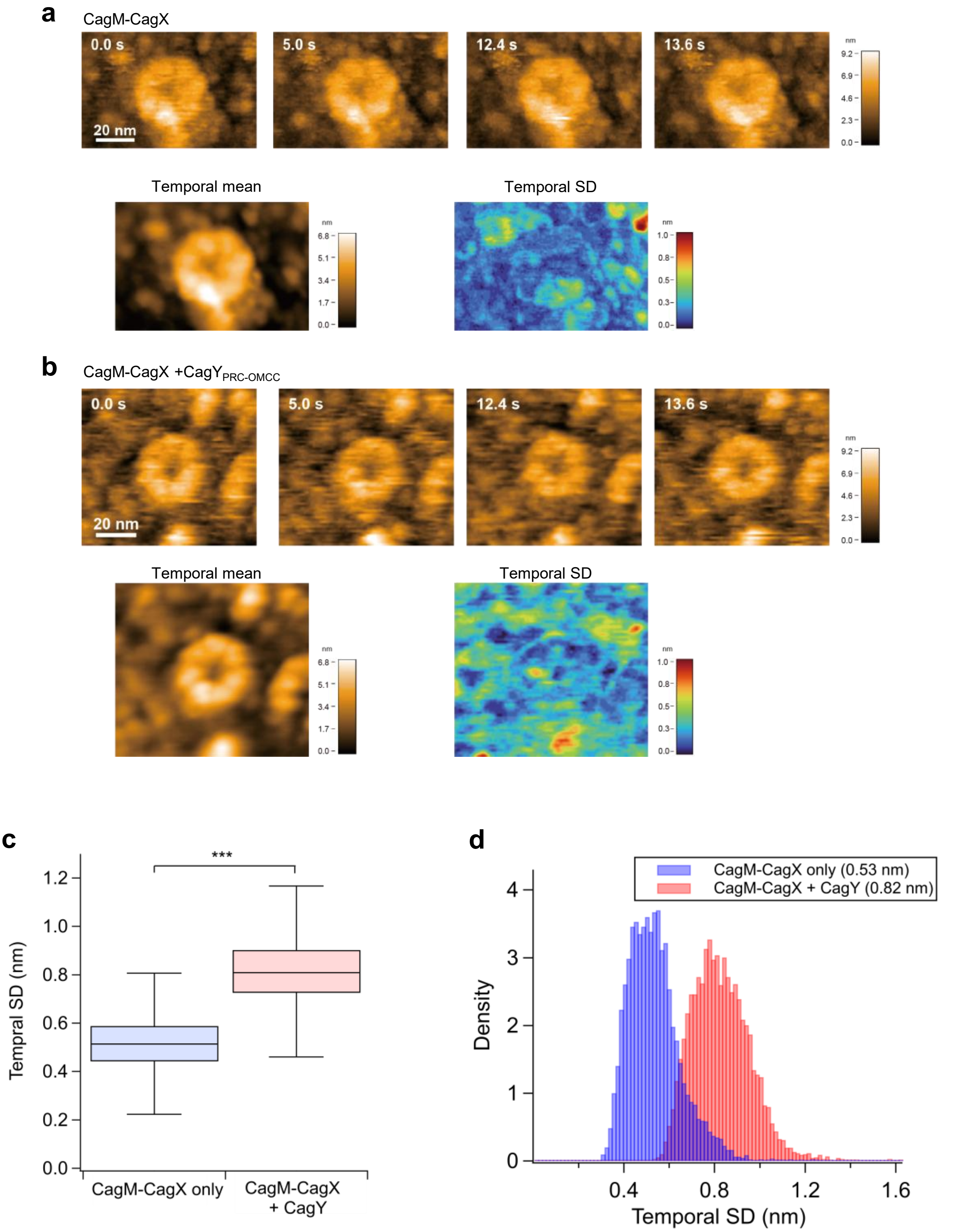

Supplementary Figure 25. Effect of CagY$_{PRC-OMCC}$ addition on pre-assembled CagM-CagX rings assessed by temporal SD analysis. **a** High-resolution time-lapse HS-AFM images of a CagM-CagX ring without CagY$_{PRC-OMCC}$ (upper row), with the corresponding temporal mean and temporal standard deviation (SD) maps computed from 95 consecutive frames (lower row). Images were acquired in continuous scanning mode at 0.2 s/frame. Scale bar, 20 nm. **b** A CagM-CagX ring after addition of CagY$_{PRC-OMCC}$, shown in the same format as **(a)**. Images were acquired under the same conditions (continuous scanning mode at 0.2 s/frame). The temporal SD is markedly elevated compared to **(a)**, indicating increased height fluctuations caused by CagY$_{PRC-OMCC}$ molecules transiently interacting with the AFM tip as they diffuse near the ring surface. Scale bar, 20 nm. **c** Box plot comparison of temporal SD values within the ring region. CagY$_{PRC-OMCC}$ addition significantly increased the temporal SD (median: 0.81 vs. 0.51 nm; $p < 10^{-10}$, Mann–Whitney U test). **d** Histogram of temporal SD distributions for CagM-CagX only (blue; peak at 0.53 nm) and CagM-CagX + CagY$_{PRC-OMCC}$ (red; peak at 0.82 nm). The rightward shift upon CagY$_{PRC-OMCC}$ addition confirms the presence of freely diffusing CagY$_{PRC-OMCC}$ near the ring. The temporal mean images remain essentially unchanged between **(a)** and **(b)**, indicating that CagY$_{PRC-OMCC}$ does not stably incorporate into the pre-assembled CagM-CagX complex, consistent with the strict sequential assembly order demonstrated in Supplementary Figure 24.

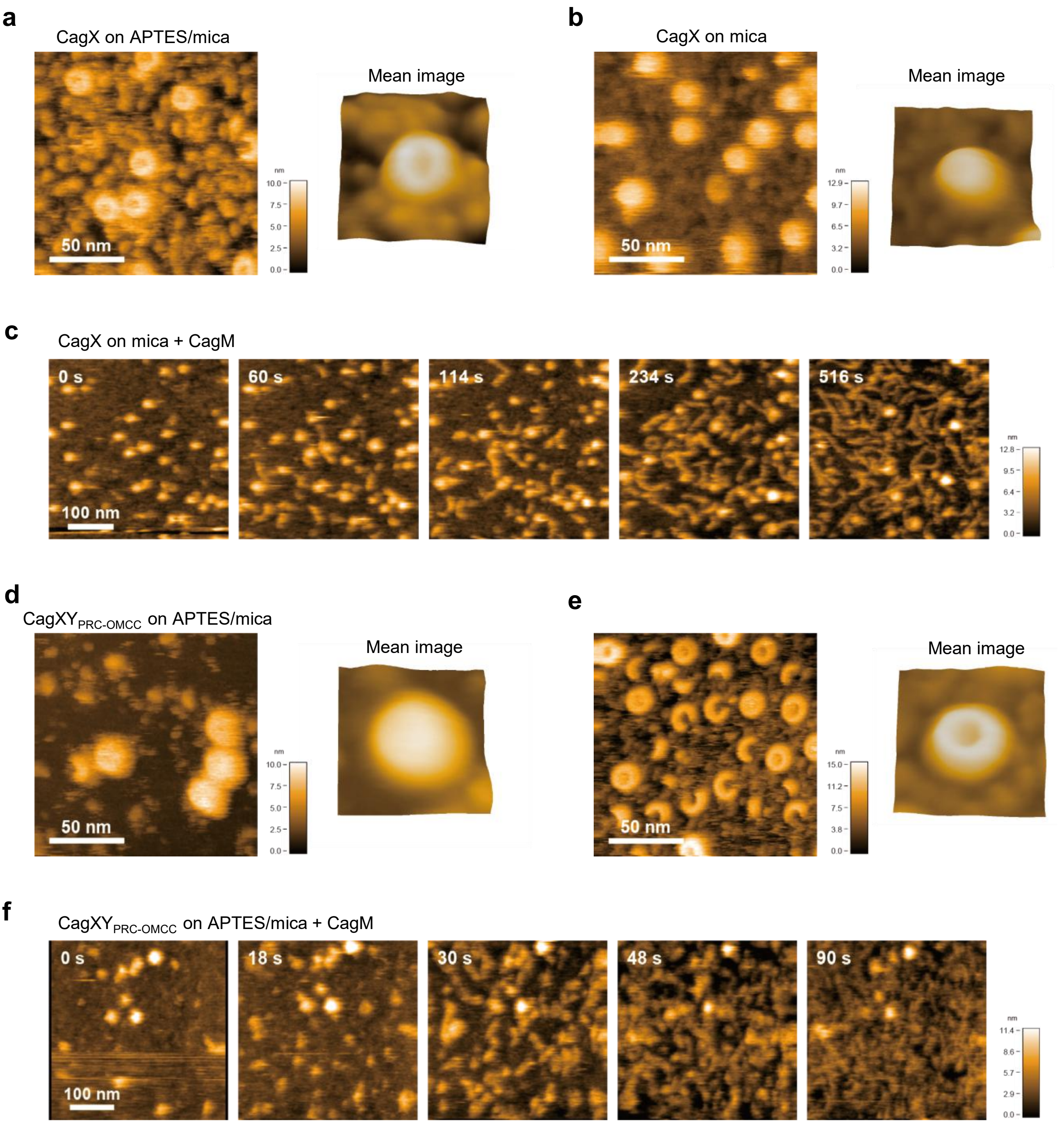

Supplementary Figure 26. Substrate-dependent orientation of CagX and CagX-CagY$_{PRC\text{-}OMCC}$ rings and its effect on CagM complex formation. a HS-AFM images of CagX rings on APTES-treated mica (left: wide-field; right: mean image). Ring pores are clearly visible, indicating the OMC-to-PR face is oriented upward. Scale bar, 50 nm. **b** HS-AFM images of CagX rings on bare mica (left: wide-field; right: mean image). Pores are not visible, indicating the PR-to-OMC face is oriented upward. The flexible periplasmic insertion (PI) domains of CagX, which are unresolved in cryo-EM structures, likely occlude the central pore when viewed from the PR face. Scale bar, 50 nm. **c** Time-lapse HS-AFM images of adding CagM to CagX rings on bare mica (intermittent scanning mode with 1 frame on and 5 frames off at 1 s/frame). Despite the presence of CagM monomers around the rings, no double-ring formation was observed. Scale bar, 100 nm. **d** HS-AFM images of CagX-CagY$_{PRC\text{-}OMCC}$ complex on APTES-treated mica (left: wide-field; right: mean image). In contrast to CagX alone **(a)**, pores are not visible, indicating a reversed orientation (PR-to-OMC face up). Scale bar, 50 nm. **e** HS-AFM images of CagX-CagY$_{PRC\text{-}OMCC}$ complex on bare mica (left: wide-field; right: mean image). In contrast to CagX alone **(b)**, pores are visible, confirming the reversed orientation (OMC-to-PR face up). Scale bar, 50 nm. **f** Time-lapse HS-AFM images of CagM addition to CagX-CagY$_{PRC\text{-}OMCC}$ complex on APTES-treated mica (intermittent scanning mode with 1 frame on and 5 frames off at 1 s/frame). Similarly to CagX alone on bare mica **(c)**, no double-ring formation was observed, consistent with the PR face being oriented upward due to the reversed orientation of the CagX-CagY$_{PRC\text{-}OMCC}$ complex on APTES-treated mica. Scale bar, 100 nm. These results demonstrate that the substrate surface chemistry controls ring orientation rather than oligomerization itself, and that CagY binding reverses the orientation of the ring on each substrate.

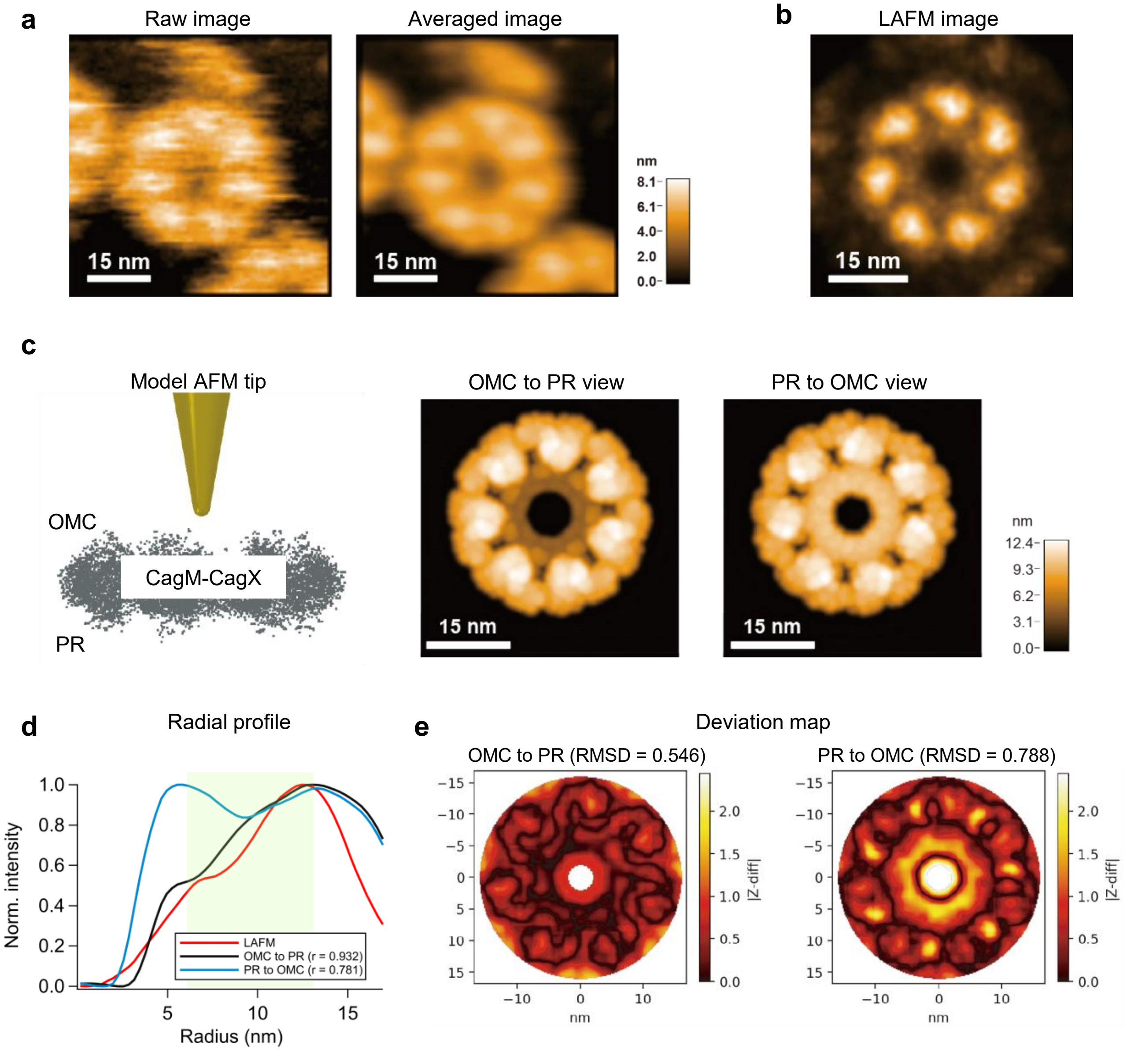

Supplementary Figure 27. Identification of the observation face of the CagM-CagX complex. **a** Representative raw HS-AFM image (left) and averaged image of 59 frames (right) of the CagM-CagX complex. Scale bars, 15 nm. **b** LAFM image reconstructed from the same 59 frames with 7-fold rotational symmetry applied. Scale bar, 15 nm. **c** Pseudo-AFM images generated from the cryo-EM map (EMD-69626) obtained in this study for OMC-to-PR and PR-to-OMC orientations, using a tip radius of 1 nm, cone angle of 10°, and 0.5 nm^{-1} spatial low-pass filter. Scale bars, 15 nm. **d** Radial profile comparison between the LAFM image (red) and pseudo-AFM images for OMC-to-PR (black; r = 0.932) and PR-to-OMC (cyan; r = 0.781) views. The green shaded region (radius ~6–13 nm) indicates the rigid ring wall core, where the cryo-EM structure is well-resolved and unaffected by missing flexible loops or disordered domains. The orientation assignment is based on profile comparison within this region to avoid potential artifacts arising from incomplete atomic coordinates in the PDB structure. **e** Deviation maps between the LAFM image and pseudo-AFM images for OMC-to-PR (RMSD = 0.546) and PR-to-OMC (RMSD = 0.788) views. The lower RMSD and higher correlation for the OMC-to-PR view indicate that the OMC face of the CagM-CagX complex is oriented upward under HS-AFM observation.

Supplementary Table 1. Summary of HDX-MS.

Data set	CagM	CagX	CagM-CagX
HDX reaction details	20 mM HEPES, 300 mM NaCl, pD 7.5, 94% D_2O, 25°C		
HDX time course (min)	0.5, 1, 10, 60, 120		
HDX control samples	Undeuterated samples		
# of peptides	83	52	CagM: 83<br>CagX: 52
Sequence coverage	93.2%	81.4%	CagM: 93.2%<br>CagX: 81.4%
Average peptide length / Redundancy	13.6 / 3.33	15 / 1.91	CagM: 13.6 / 3.33<br>CagX: 15 / 1.91
Replicates	3 (technical)		
Repeatability	0.13	0.09	CagM: 0.12<br>CagX: 0.11
Significant differences in HDX	95% confidence interval (CagM: 0.45 Da, CagX: 0.36 Da)		

Data set	CagM	$CagM_{NTD-EK}$	CagM	$CagM_{DEK}$
HDX reaction details	20 mM HEPES, 300 mM NaCl, pD 7.5, 96% D_2O, 25°C			
HDX time course (min)	0.5, 1, 10, 60, 120			
HDX control samples	Undeuterated samples			
# of peptides	51	50	72	72
Sequence coverage	84.1%	84.1%	92.6%	92.6%
Average peptide length / Redundancy	12.3 / 1.97	12.3 / 1.97	13.3 / 2.83	13.3 / 2.83
Replicates	3 (technical)			
Repeatability	0.13	0.07	0.14	0.11
Significant differences in HDX	95% confidence interval (0.38 Da)		95% confidence interval (0.46 Da)	

Data set	CagX	$CagY_{PRC-OMCC}$	CagX-$CagY_{PRC-OMCC}$
HDX reaction details	20 mM HEPES, 300 mM NaCl, pD 7.5, 90% D_2O, 25°C		
HDX time course (min)	0.5, 1, 10, 60, 120		
HDX control samples	Undeuterated samples		
# of peptides	72	64	CagX: 72<br>CagY: 64
Sequence coverage	82.2%	79.1%	CagX: 82.2%<br>CagY: 79.1%
Average peptide length / Redundancy	13.1 / 2.28	12.3 / 2.27	CagX: 13.1 / 2.28<br>CagY: 12.3 / 2.27
Replicates	3 (technical)		
Repeatability	0.11	0.08	CagX: 0.11<br>CagY: 0.10
Significant differences in HDX	95% confidence interval (CagX: 0.39 Da, CagY: 0.32 Da)		

Supplementary Table 2. Cryo-EM data collection, refinement and validation statistics.

	**CagM-CagX** EMD-69626	**CagX-CagY$_{PRC-OMCC}$ C16** EMD-65051 PDB ID: 9VGU	**CagX-CagY$_{PRC-OMCC}$ C17** EMD-65048 PDB ID: 9VGF
**Data collection and processing**			
Magnification	105,000	105,000	105,000
Voltage (kV)	300	300	300
Electron exposure (e^-/Å^2)	50	50	50
Defocus range (μm)	-1.5 to -2.5	-1.5 to -2.5	-1.5 to -2.5
Pixel size (Å)	1.494	0.83	0.83
Symmetry imposed	C7	C16	C17
Initial particle images (no.)	1,767,379	1,731,789	1,277,522
Final particle images (no.)	190,251	406,996	373,053
Map resolution (Å)	7.77	2.87	2.58
FSC threshold	0.143	0.143	0.143
Map resolution range (Å)	3.13-18.49	1.8-34.6	1.8-42.0
**Refinement**			
Model resolution (Å)	/	3.5	3.5
FSC threshold	/	0.5	0.5
Map sharpening *B* factor (Å^2)	/	-118.5	-88.6
Model composition			
Non-hydrogen atoms	/	29,536	31,705
Protein residues	/	3,664	3,927
Ligands	/	0	0
Residues modelled			
CagX	/	32-124, 261-320	32-125, 261-320
CagY	/	1469-1544	1469-1545
R.m.s deviations			
Bond lengths (Å)	/	0.003	0.003
Bond angles (°)	/	0.582	0.651
**Validation**			
MolProbity score	/	1.87	1.81
Clashscore	/	8.36	8.78
Poor rotamers (%)	/	0.27	0.14
Ramachandran plot			
Favored (%)	/	93.64	95.16
Allowed (%)	/	6.36	4.84
Disallowed (%)	/	0.00	0.00
