# Supplementary material for: Deciphering the initiation pathway of CagT4SS assembly by in vitro reconstitution of subcomplexes
Source: Nat Commun. 2026 Jul 23;17:8987. doi: 10.1038/s41467-026-75820-0 (PMC13507270; doi:10.1038/s41467-026-75820-0)
Supplement: Supplementary file 3 [file 41467_2026_75820_MOESM28_ESM.pdf]

nature portfolio

Peer Review File

Deciphering the initiation pathway of CagT4SS assembly by *in vitro* reconstitution of subcomplexes

Corresponding Author: Professor Shannon Wing Ngor Au

This file contains all reviewer reports in order by version, followed by all author rebuttals in order by version.

Attachments originally included by the reviewers as part of their assessment can be found at the end of this file.

Version 0:

Reviewer comments:

Reviewer #1

(Remarks to the Author)
In this study, Chu et al. apply multiple biophysical approaches, including cryo-EM, HDX-MS, and HS-AFM, to investigate the CagT4SS initiation pathway. The authors successfully reconstitute in vitro assemblies of CagX, CagY, and CagM, and delineate the assembly sequence. They show that CagX spontaneously oligomerizes into ring structures that act as a scaffold for PRC initiation and subsequent incorporation of CagY and CagM. CagY associates with these CagX rings as monomers, stabilizing the ring architecture. This intact PRC then serves as a docking hub for CagM, which, upon binding, forms the outer rim of the double-ring structure. The ability of CagM to form higher-order oligomers is essential for its incorporation into the CagX-CagY ring (as dimers). The static structures of intermediate complexes (e.g., CagX-CagM) are revealed by cryo-EM, whereas HS-AFM captures their interaction dynamics. HDX-MS further identifies specific interaction regions.
I was invited to evaluate the HS-AFM analysis. Overall, the HS-AFM experiments are well-designed, technically sound, and the data quality is high. The HS-AFM results generally support the authors' model and are consistent with observations from other methods. However, the current HS-AFM analysis lacks essential quantitative measurements and statistical evaluation, which are required for drawing robust conclusions. Additional clarification, annotation, and quantitative analyses are needed for the acceptance of the manuscript.
Below I provide detailed comments by section (that involve HS-AFM analysis):
1. "Self-oligomerization of CagX may act as a scaffold for early CagT4SS assembly"
The data generally support the authors' conclusions. However, the influence of the substrate (mica vs. APTES-treated mica) on protein assembly requires explicit discussion. Surface charge (negatively charged mica vs. positively charged APTES) can influence oligomerization behavior. The authors should clarify why different substrates were used across experiments and discuss potential effects on assembly dynamics.
For HS-AFM imaging of monomeric molecules (Supplementary Fig. 2B), the CagY images are confusing: it seems to me that two CagY monomers (two large PRC-domain blobs) consistently appear in the same image field. Are they always coupled? This needs clarification.
For the observation of different CagM oligomeric species, Supplementary Movie 3 is difficult to interpret. I suggest the authors consider clearer annotation or additional representative data would strengthen this point.
2. "In vitro reconstitution of CagM-CagX complex revealing a double-ring complex"
The HS-AFM data clearly indicate a dynamic double-ring assembly consistent with the authors' model. However, the sidedness assignment of the CagM-CagX assembly based on height profiles is not convincing. The reported height profiles do not align better with the pseudo-AFM "top-view", as compared to the "bottom-view".
Moreover, the pseudo-AFM images are generated from incomplete PDB structures with substantial unresolved regions. These missing regions would contribute to AFM densities and therefore bias the topology analysis. Additionally, it is unclear why PDB 6X6J, annotated as a CagX-CagY assembly in the PDB website, was used for comparison with CagX-CagM AFM results. Nonetheless, I agree with the conclusion that the AFM-observed ring does not include the OMCC, given the large height discrepancy.
3. "Distinct interfaces in CagM homo-oligomerization and hetero-oligomerization with CagX"
The HS-AFM data support the claim that CagM mutants incapable of homo-oligomerization fail to incorporate into the CagX ring.
4. "HS-AFM captures the in vitro assembly of the CagX-CagY-CagM complex in real time"

The HS-AFM movies are of high quality. However, the analysis lacks quantitative measurements and statistics needed to substantiate the authors' conclusions.
To support the claim that CagY binding stabilizes CagX rings, the authors should quantify ring flexibility (e.g., contour deformation, frame-by-frame variance) and provide statistical comparisons. I think the observation that "CagX-CagY is more readily visualized" is not sufficient here. Likewise, analysis of additional ring particles is needed to robustly characterize ring dimensions and distinguish CagX rings from CagX-CagY rings, given their diameter difference of 4 nm.
An especially valuable addition would be quantifying CagM binding kinetics. Since CagM association and dissociation events are visible in the movies, extracting dissociation rates for CagM on CagX-only versus CagX-CagY rings would directly assess whether CagY enhances CagM stabilization, and provides some energetics. I believe this result would significantly strengthen the study.

Reviewer #2

(Remarks to the Author)
Review of manuscript NCOMMS-25-87239

The authors did an impressive amount of work shedding new light on the T4SS assembly process based on in vitro reconstitution from purified components. They used cutting edge biochemical, biophysical and structural biology methods to characterize CagX, CagM and CagY and their interactions. The analyzed proteins are in many cases membrane-associated and biochemically very difficult to work with. The presented work is overall quite impressive, but the manuscript is very densely written and will be difficult to understand for non-experts. Since I could not visualise any of the HS-AFM videos, it is not possible to conclude whether the presented evidence supports the conclusions.

Major concerns:

1. Note that this review is only partial, since I could not get any of the video files to work.
2. The work is impressive, but the authors make very strong statements on their impact for the assembly process (Fig. 6). What happens in vivo may well be different from what can be reconstituted in vitro from purified components that represent only a domain in case of CagY. The work is still very interesting, but the language on the mechanistic impact should be toned down.
3. The interpretation of the CagM mutant analysis is not convincing (lines 221-250). The authors created deletion and E/K (changing the charge) variants of CagM. These mutations my impact protein folding and stability, or they may be directly involved in ring formation. The presented data do not support the claim that interactions between the mutated residues are directly involved in ring formation. The authors may consider leaving this less well substantiated section out of the manuscript (also lines 252-270 and 411-416). At the very least, they should discuss the limitations of their approach (lines 411-416).

Minor concerns:

1. Line 43 refers to a "pilus". As far as I know, this has not been established with certainty in the case of the H. pylori T4SS.
2. Which domains of CagX, CagY and CagM were purified? This should be clarified earlier in the results section (beginning lines 91) and in the legend to Fig.1
3. Suppl Fig. 3B particles not easy to identify. They should be indicated by arrows.
4. Microscale thermophoresis appears to be mentioned only in the Materials and methods section. Was it used in the manuscript?
5. The presented assembly model is interesting, but the authors do not really consider that in vivo, CagY is a very long protein with many repeat regions. They characterized only the C-terminal membrane domains. It would be interesting to speculate how the N-terminal repeat regions fit into the model presented in Fig. 6.
6. Considering the nature of the proteins, it is somewhat surprising that detergents were not required for the purification.

Reviewer #3

(Remarks to the Author)
I co-reviewed this manuscript with one of the reviewers who provided the listed reports. This is part of the Nature Communications initiative to facilitate training in peer review and to provide appropriate recognition for Early Career Researchers who co-review manuscripts.

Reviewer #4

(Remarks to the Author)
Review attached below.

Reviewer #5

(Remarks to the Author)
General comments to the authors
The authors have presented an intriguing manuscript that describes their efforts to study the stepwise assembly and

dynamics of a nanomachine known as a virulence factor that contributes to the pathogenicity of H. pylori. Below are several comments, questions, and suggestions that can be used to improve the manuscript and ready it for publication.
First, the authors have mostly conformed to the modern standard adopted by the HDX MS community for data reporting. It doesn't appear that the author have uploaded the raw MS data to a specific archive. The following will address this problem: Upload raw MS files, peptide lists, and summary processed data to the PRIDE archive, or similar. I would also suggest condensing the individual HDX supplemental data files into one workbook for ease of presentation and data mining.
Next, there seems to be some agreement issues with the peptide level HDX. Two examples:
-Supplementary Figure 5, data for peptide 305-321 seems out of place considering overlapping peptides.
-Supplementary Figure 10, peptides 39-56 and 42-58 show conflicting data.
This is likely due to peptide misidentification in PLGS. I would just go through and confirm peptide ID's that seem incongruent.
The authors use increases or decreases in solvent exposure as reasons for changes in HDX. It would be better to include in this discussion decreases and increases in h-bonding strength and character as well. The HDX process requires both access to solvent and catalyst as well as the breaking of potential h-bonds to the backbone amide hydrogen in order for productive HDX to occur.s
How stable are the SEC-isolated oligomer structures? Have the SEC fractions for any of the complexes been re-injected onto the column to see if those isolated fractions have then re-equilibrated to the distributions before SEC separations? Along those same lines, was it possible to see distributions/pouplations in the isotopic envelopes in the raw HDX data that reflect the range in oligomerization states that are reported from the cryo-EM data? It would be curious if there were differences in the HDX of the monomeric units depending on the oligomerization sate.
Line 115: "…are composed of arrowhead-like units…". I think it would be more accurate to describe the shape as a 3-lobed epitrochoid or reuleaux-like triangle.
Line 192 and 255: instead of "overlapping" perhaps consider using coincident peptides. I think that overlapping is confusing when it is also used to describe peptides within a protein construct.
Line 258: "…may be due to their differences in oligomeric states, resulting in distinctive pepsin digestion patterns." I think that this is only half-correct. At the point of digestion, the protein sample is quenched to low pH and temperature. SO, differences in the acid denatured states that arrive from the different oligomeric states could be the reason for the differences in digestion patterns.

Version 1:

Reviewer comments:

Reviewer #1

(Remarks to the Author)
The authors have performed additional HS-AFM experiments and quantitative anaylsis, which significantly strengthened this work. All of my previous concerns have been addressed. I now fully support the publication of this work.

Reviewer #2

(Remarks to the Author)
The authors have carefully addressed the reviewers's suggestions and I have no additional comments. They should be commended for this carefully executed study and for their detailed and constructive response to all reviewer comments.

Reviewer #3

(Remarks to the Author)
I co-reviewed this manuscript with one of the reviewers who provided the listed reports. This is part of the Nature Communications initiative to facilitate training in peer review and to provide appropriate recognition for Early Career Researchers who co-review manuscripts.

Reviewer #4

(Remarks to the Author)
The authors were very responsive to the reviews. They have addressed my questions and I appreciate the care and effort they went about in their response and edits. I can not comment the HDX or AFM revisions since this is not my area of expertise.

The only thing I feel needs to be addressed in the revision is that the authors should add the FSC curve and Euler angle plot to supplemental figure 3 and an Euler angle plot to supplemental figure 17. If there are any 2D averages that are not a "top" or "bottom" views - these would also be helpful to include - but the Euler plots should be sufficient to show the extent (or not) of preferred orientation. I apologize for not requesting the change to the current supplemental 17 in the first revision.

Reviewer #5

(Remarks to the Author)
I thank the authors for taking the time to address my "comments, questions, and suggestions". I have nothing more to add,

and I cannot wait to see the final version in press.

Open Access This Peer Review File is licensed under a Creative Commons Attribution 4.0 International License, which permits use, sharing, adaptation, distribution and reproduction in any medium or format, as long as you give appropriate credit to the original author(s) and the source, provide a link to the Creative Commons license, and indicate if changes were made.
In cases where reviewers are anonymous, credit should be given to 'Anonymous Referee' and the source.
The images or other third party material in this Peer Review File are included in the article's Creative Commons license, unless indicated otherwise in a credit line to the material. If material is not included in the article's Creative Commons license and your intended use is not permitted by statutory regulation or exceeds the permitted use, you will need to obtain permission directly from the copyright holder.
To view a copy of this license, visit https://creativecommons.org/licenses/by/4.0/

RESPONSE TO REVIEWERS' COMMENTS

Reviewer #1:

Overall comments: *In this study, Chu et al. apply multiple biophysical approaches, including cryo-EM, HDX-MS, and HS-AFM, to investigate the CagT4SS initiation pathway. The authors successfully reconstitute in vitro assemblies of CagX, CagY, and CagM, and delineate the assembly sequence. They show that CagX spontaneously oligomerizes into ring structures that act as a scaffold for PRC initiation and subsequent incorporation of CagY and CagM. CagY associates with these CagX rings as monomers, stabilizing the ring architecture. This intact PRC then serves as a docking hub for CagM, which, upon binding, forms the outer rim of the double-ring structure. The ability of CagM to form higher-order oligomers is essential for its incorporation into the CagX-CagY ring (as dimers). The static structures of intermediate complexes (e.g., CagX-CagM) are revealed by cryo-EM, whereas HS-AFM captures their interaction dynamics. HDX-MS further identifies specific interaction regions.*

I was invited to evaluate the HS-AFM analysis. Overall, the HS-AFM experiments are well-designed, technically sound, and the data quality is high. The HS-AFM results generally support the authors' model and are consistent with observations from other methods. However, the current HS-AFM analysis lacks essential quantitative measurements and statistical evaluation, which are required for drawing robust conclusions. Additional clarification, annotation, and quantitative analyses are needed for the acceptance of the manuscript. Below I provide detailed comments by section (that involve HS-AFM analysis):

Response: We thank Reviewer #1 for the thorough and expert evaluation of the HS-AFM analysis. The reviewer's detailed suggestions have led to substantial improvements, including new quantitative analyses of ring stability, CagM binding kinetics, and face identification, which we believe significantly strengthen the study. All comments have been addressed in the revised manuscript. Please refer to the point-to-point response below.

1) ***Comments****: "Self-oligomerization of CagX may act as a scaffold for early CagT4SS assembly" The data generally support the authors' conclusions. However, the influence of the substrate (mica vs. APTES-treated mica) on protein assembly requires explicit discussion. Surface charge (negatively charged mica vs. positively charged APTES) can influence oligomerization behavior. The authors should clarify why different substrates were used across experiments and discuss potential effects on assembly dynamics.*

Response: We thank the reviewer for this important comment. We have added a detailed discussion of substrate selection strategy and its mechanistic significance.

Following standard HS-AFM procedures, we initially used bare mica for all experiments and tested APTES-treated mica when adsorption was insufficient. For complex formation experiments, we performed parallel tests on both substrates and selected the one providing optimal observation conditions.

An important finding was that the appearance of CagX rings differs markedly between the two substrates: on bare mica, the central pore of the ring was not visible, whereas on APTES-mica, the pore was clearly resolved (Supplementary Fig. 26A-B). When CagM

was added, double-ring formation was only observed in CagX immobilized on APTES-mica but not on bare mica (Fig. 2B and Supplementary Fig. 26C). Based on the resolved structures and our biochemical analyses, the CagM binding interface is located on the OMC face of CagX. We suspected that the mica may affect the particle orientation. To confirm this, the orientation of observed CagM-CagX complex was characterized using localization AFM and pseudo-AFM, confirming the OMC face facing upwards under APTES-mica (Supplementary Fig. 27). These results collectively supported that the OMC face is presumably exposed under APTES-mica and available for CagM binding while the OMC face was facing the substrate on bare mica, obstructing the CagM binding.

Interestingly, for the CagX–CagY$_{PRC\text{-}OMCC}$ complex, the pore visibility pattern was reversed: on APTES-mica, pores were not visible, while on bare mica, pores were clearly resolved (Supplementary Fig. 26D–E). This suggests that CagY$_{PRC\text{-}OMCC}$ binding alters the physicochemical properties of the ring surface, reversing its preferred orientation on each substrate. Consistent with this interpretation, CagM addition to CagX-CagY$_{PRC\text{-}OMCC}$ on APTES-mica, where the OMC face would now be facing down, failed to form double rings (Supplementary Fig. 26F). Taken together, these results provide internally consistent evidence that CagM binding is strictly orientation-dependent: double-ring formation occurs only when the OMC face is accessible, regardless of whether the scaffold is CagX alone or CagX-CagY$_{PRC\text{-}OMCC}$. The substrate surface chemistry controls ring orientation rather than oligomerization itself. Yet, the surface charge of CagX alone was not able to explain its preferred orientation on bare or APTES-treated mica. We reasoned that it involves a complex mechanism, likely combining electrostatic, hydrophobic, contact-area and flexibility effects, with a rigorous dissection acknowledged as beyond the scope of this study.

While substrate effects cannot be entirely excluded, the key findings, orientation-dependent CagM binding and CagY-mediated stabilization, are supported by orthogonal evidence from cryo-EM and HDX-MS. This discussion has been incorporated into the revised manuscript and supplementary materials.

Please refer to our revised manuscript [Discussion (line 486-499) and Methodology (line 729-735, 778-782)].

> ***Comments**: For HS-AFM imaging of monomeric molecules (Supplementary Fig. 2B), the CagY images are confusing: it seems to me that two CagY monomers (two large PRC-domain blobs) consistently appear in the same image field. Are they always coupled? This needs clarification.*

Response: We thank the reviewer for this comment. The appearance of "two coupled blobs" in the original images was due to two independent CagY$_{PRC\text{-}OMCC}$ molecules that happened to be present in the same field of view; they are not always coupled. However, we acknowledge that the image quality in the original data was insufficient to make this point clearly. We therefore performed new HS-AFM experiments with optimized imaging conditions (Supplementary Fig. 2B, Supplementary Movie 2). In the new data, individual CagY$_{PRC\text{-}OMCC}$ molecules were clearly resolved as a two-domain structure connected by a flexible PL linker, where the larger and smaller globular domains likely correspond to PRC and OMCC, respectively.

Furthermore, we performed a quantitative comparison of end-to-end distances

(Supplementary Fig. 2C). CagX exhibited a mean distance of 8.8 ± 0.1 nm (n = 566), while $CagY_{PRC-OMCC}$ showed 15.0 ± 0.1 nm (n = 233). Please refer to our revised manuscript [Results (line 135-142)].

***Comments**: For the observation of different CagM oligomeric species, Supplementary Movie 3 is difficult to interpret. I suggest the authors consider clearer annotation or additional representative data would strengthen this point.*

Response: We thank the reviewer for this suggestion. In the revised manuscript, clear annotations have been added to the CagM adsorption process figure (Fig. 1F). Each stage is now labelled along the time course: monomers and small oligomers (arrows, 1.33 min) → filamentous oligomers (dashed circles, 6.48 min) → ring oligomers (dashed circles, 12.87–14.30 min). This facilitates identification of each structural species in the concentration-dependent oligomerization progression. Corresponding annotations have also been added to the accompanying movie (Supplementary Movie 4).
Please refer to our revised manuscript [Results (line 147-152) and Methodology (line 752-753)].

2) ***Comments**: "In vitro reconstitution of CagM-CagX complex revealing a double-ring complex" The HS-AFM data clearly indicate a dynamic double-ring assembly consistent with the authors' model. However, the sidedness assignment of the CagM-CagX assembly based on height profiles is not convincing. The reported height profiles do not align better with the pseudo-AFM "top-view", as compared to the "bottom-view". Moreover, the pseudo-AFM images are generated from incomplete PDB structures with substantial unresolved regions. These missing regions would contribute to AFM densities and therefore bias the topology analysis. Additionally, it is unclear why PDB 6X6J, annotated as a CagX-CagY assembly in the PDB website, was used for comparison with CagX-CagM AFM results. Nonetheless, I agree with the conclusion that the AFM-observed ring does not include the OMCC, given the large height discrepancy.*

Response: The reviewer's criticism is entirely valid. We recognized that simple height profiles were insufficient for face identification and have substantially revised the analysis (Supplementary Fig. 27).
We generated averaged images from 59 frames of the CagM–CagX complex and reconstructed a localization AFM (LAFM) image with 7-fold rotational symmetry applied (Supplementary Fig. 27A–B). Pseudo-AFM images were generated from the low resolution cryo-EM map of CagM-CagX obtained in this study (EMD-69626 instead of PDB 6X6J as in the original), using a tip radius of 1.0 nm, cone angle of 10°, and 0.5 nm^{-1} spatial low-pass filter (Supplementary Fig. 27C). The following quantitative comparisons were performed:
Radial profile correlation (Supplementary Fig. 27D): OMC-to-PR view r = 0.932 vs. PR-to-OMC view r = 0.781
Deviation map RMSD (Supplementary Fig. 27E): OMC-to-PR view RMSD = 0.546 vs. PR-to-OMC view RMSD = 0.788
These results consistently indicate that we are observing the OMC-to-PR face. The

AFM-observed height (~8 nm) does not correspond to the full native PRC-OMCC height (~18 nm) including the peripheral OMCC components (CagT and Cag3, which are absent from our reconstitution). This is consistent with the OMC face of the inner ring being oriented upward and accessible to the AFM tip.

To address the reviewer's concern about "bias from unresolved regions," the orientation assignment was based on profile comparison within the rigid ring wall core region (radius ~6–13 nm; green shaded region in Supplementary Fig. 27D), where the cryo-EM structure is well-resolved and unaffected by missing flexible loops. In this critical region, the LAFM image shows a steep ascending gradient (positive slope) that is reproduced exclusively by the OMC-to-PR view. In contrast, the PR-to-OMC view predicts a descending gradient in this region, which is fundamentally incompatible with the observed topography.

Please refer to our revised manuscript [Discussion (line 487-492) and Methodology (line 786-787)].

3) ***Comments****: "Distinct interfaces in CagM homo-oligomerization and hetero-oligomerization with CagX" The HS-AFM data support the claim that CagM mutants incapable of homo-oligomerization fail to incorporate into the CagX ring.*

Response: We appreciate the reviewer agrees with our claim.

4) ***Comments****: "HS-AFM captures the in vitro assembly of the CagX-CagY-CagM complex in real time" The HS-AFM movies are of high quality. However, the analysis lacks quantitative measurements and statistics needed to substantiate the authors' conclusions. To support the claim that CagY binding stabilizes CagX rings, the authors should quantify ring flexibility (e.g., contour deformation, frame-by-frame variance) and provide statistical comparisons. I think the observation that "CagX-CagY is more readily visualized" is not sufficient here. Likewise, analysis of additional ring particles is needed to robustly characterize ring dimensions and distinguish CagX rings from CagX-CagY rings, given their diameter difference of 4 nm.*

Response: We thank the reviewer for this most important comment. We have comprehensively quantified the structural stability and morphology of CagX and CagX-$CagY_{PRC-OMCC}$ rings using both dynamic and static analyses.

Dynamic stability analysis (Supplementary Fig. 22): We analyzed CagX (n = 15) and CagX–$CagY_{PRC-OMCC}$ (n = 11) rings under continuous HS-AFM scanning.

- Image correlation over time (Supplementary Fig. 22C): The Pearson correlation coefficient between each frame and the first frame was tracked over the normalized observation period. CagX rings showed progressive structural collapse (declining correlation), whereas CagX-$CagY_{PRC-OMCC}$ rings maintained high structural integrity.
- Structural Retention (Supplementary Fig. 22D): Defined as the mean correlation over the final 25% of frames. CagX-$CagY_{PRC-OMCC}$ rings exhibited significantly higher retention than CagX rings (median: 0.86 vs. 0.68; $p = 8.0 \times 10^{-5}$, Mann–Whitney U test).
- Circularity fluctuation (Supplementary Fig. 22E–F): The coefficient of variation (CV)

of circularity was significantly lower for CagXY rings (median CV: 0.0022 vs. 0.0108; $p = 4.1 \times 10^{-5}$, Mann–Whitney U test), indicating that CagY binding confers mechanical stability.

Static morphology analysis (Supplementary Fig. 23): We compared CagX (n = 45) and CagX-$CagY_{PRC-OMCC}$ (n = 39) ring particles.

- Ring height: CagX-$CagY_{PRC-OMCC}$ > CagX ($p < 0.001$). The ring becomes taller upon $CagY_{PRC-OMCC}$ binding.
- FWHM diameter: Not significantly different. The expected ~4 nm diameter difference may be masked by tip broadening effects.
- Circularity: CagX-$CagY_{PRC-OMCC}$ > CagX ($p < 0.001$). CagX-$CagY_{PRC-OMCC}$ rings are more circular, indicating greater structural regularity.

These quantitative results strongly support the claim that $CagY_{PRC-OMCC}$ binding structurally stabilizes CagX rings. This is also consistent with the HDX-MS data (Fig. 4), which show reduced conformational dynamics of CagX upon $CagY_{PRC-OMCC}$ binding. These new analyses have been incorporated into the revised supplementary materials and are referenced in the main text [Results (line 360-377), Discussion (line 437-440) and Methodology (line 792-800)].

Comments: *An especially valuable addition would be quantifying CagM binding kinetics. Since CagM association and dissociation events are visible in the movies, extracting dissociation rates for CagM on CagX-only versus CagX-CagY rings would directly assess whether CagY enhances CagM stabilization, and provides some energetics. I believe this result would significantly strengthen the study.*

Response: We thank the reviewer for this valuable suggestion, which has led to one of the most significant additions to the revised manuscript. We have comprehensively quantified CagM binding and dissociation dynamics on both CagX rings and CagX-CagYPRC-OMCC rings (Fig. 6).

To determine the binding state of CagM at each position around the ring, we divided the outer annular region into equal angular sectors and monitored the mean height of each sector over time. A sector with elevated height was classified as CagM-bound, while a sector at the baseline height was classified as unbound. The resulting spatiotemporal binding patterns were visualized as angular kymographs. Dwell times for bound and unbound states were extracted from these kymographs and fitted with single-exponential decay functions, yielding the following results:

CagM on CagX rings (Fig. 6C-D; 2,024/2,033 bound/unbound events from 10 molecules):

- Bound-state dwell time: $\tau = 0.229 \pm 0.005$ s
- Unbound-state dwell time: $\tau = 0.195 \pm 0.005$ s
- Cooperativity: 2.7-fold (adjacent bound: 26% vs. adjacent unbound: 9.7%)

CagM on CagX-CagYPRC-OMCC rings (Fig. 6G-H; 807/800 events from 6 molecules):

- Bound-state dwell time: $\tau = 0.380 \pm 0.016$ s (1.7× longer than on CagX)
- Unbound-state dwell time: $\tau = 0.423 \pm 0.019$ s (2.2× longer than on CagX)
- Cooperativity: 4.2-fold (adjacent bound: 22.0% vs. adjacent unbound: 5.2%; 1.6× higher than CagX)

We note that, ideally, we would like to extract intrinsic kinetic parameters from these measurements. However, the CagM–CagX interaction is inherently labile, and the

repeated mechanical contact of the AFM tip during continuous scanning inevitably perturbs the binding equilibrium to some extent. This is an intrinsic limitation of HS-AFM when applied to weakly bound complexes. Consequently, the absolute dwell time values reported here should not be taken as purely reflecting the native on/off kinetics but rather as apparent values that include contributions from tip-induced perturbation. Nevertheless, since both CagX and CagX-CagY$_{PRC-OMCC}$ rings were imaged under identical scanning conditions, the relative comparison between the two substrates remains valid. The consistently longer dwell times and higher cooperativity observed on CagX-CagY$_{PRC-OMCC}$ rings provide qualitative but robust evidence that CagY$_{PRC-OMCC}$ enhances CagM binding stability.

Moreover, HS-AFM movies revealed a striking qualitative difference in the mode of CagM association: on CagX rings, CagM subunits bind sequentially one by one to individual sectors (Supplementary Fig. 4), whereas on CagX-CagY$_{PRC-OMCC}$ rings, CagM often binds as a pre-formed ring-like assembly (Fig. 5A). This difference in binding mode is consistent with the higher cooperativity observed on CagX-CagY$_{PRC-OMCC}$ rings and suggests that CagY$_{PRC-OMCC}$ not only stabilizes individual CagM binding but also promotes concerted ring formation, potentially by providing a more geometrically or electrostatically favourable scaffold.

This new kinetic analysis has been incorporated into the revised main text (Fig. 6) and is discussed in the context of the assembly model [Results (line 400-418), Discussion (line 463-464, 475-486) and Methodology (line 802-815)].

Reviewer #2:

Overall comments: The authors did an impressive amount of work shedding new light on the T4SS assembly process based on in vitro reconstitution from purified components. They used cutting edge biochemical, biophysical and structural biology methods to characterize CagX, CagM and CagY and their interactions. The analyzed proteins are in many cases membrane-associated and biochemically very difficult to work with. The presented work is overall quite impressive, but the manuscript is very densely written and will be difficult to understand for non-experts. Since I could not visualise any of the HS-AFM videos, it is not possible to conclude whether the presented evidence supports the conclusions.

Response: We apologize that the reviewer encountered problems on viewing the HS-AFM videos. After incorporating the suggestions from Reviewer #1 and Reviewer #4, all movies in this revised manuscript are uploaded for visualization and ready for download for offline review. And here is the OneDrive link (https://shorturl.at/jRZBK). Please refer to our point-to-point response below.

Major concerns:

1) ***Comments****: Note that this review is only partial, since I could not get any of the video files to work.*

Response: We apologize for the inconvenience in accessing the video files. In the revised submission, we have ensured that all supplementary movies (Supplementary Movies 1–18) are provided in standard MP4 format and are accessible. We have also expanded the number of movies from the original submission to include all new experiments added during revision. We hope the reviewer will now be able to view all HS-AFM data.

2) ***Comments****: The work is impressive, but the authors make very strong statements on their impact for the assembly process (Fig. 6). What happens in vivo may well be different from what can be reconstituted in vitro from purified components that represent only a domain in case of CagY. The work is still very interesting, but the language on the mechanistic impact should be toned down.*

Response: We agree with this comment and have carefully tempered the language throughout the revised Discussion. Specific changes include: (1) "we pioneer" → "we report" to avoid an overly assertive tone (line 428); (2) "mimicking the native presentation of CagT4SS" → "that share key structural features with the native CagT4SS" to avoid implying equivalence between *in vitro* and *in vivo* assembly (line 429); (3) "we capture the dynamic assembly states and delineate the sequential assembly order" → "The findings from different *in vitro* biophysical analysis further provide spatial and temporal details of the dynamic assembly states beyond the fully assembled PRC-OMCC structure and allow us to propose a sequential assembly order" (line 430-432) to clarify that the assembly order is a model derived from *in vitro* data, not a definitive *in vivo* pathway; and (4) a new passage has been added at the end of the Discussion explicitly acknowledging that our findings are derived entirely from *in*

vitro reconstitution using recombinant proteins and that the assembly pathway may not fully recapitulate the process in the native bacterial periplasm, where additional factors such as the inner membrane, other T4SS components, and the cellular environment may influence the assembly sequence and kinetics (line 515-518). We hope the revised Discussion has addressed the concern from the Reviewer.

3) ***Comments:*** *The interpretation of the CagM mutant analysis is not convincing (lines 221-250). The authors created deletion and E/K (changing the charge) variants of CagM. These mutations my impact protein folding and stability, or they may be directly involved in ring formation. The presented data do not support the claim that interactions between the mutated residues are directly involved in ring formation. The authors may consider leaving this less well substantiated section out of the manuscript (also lines 252-270 and 411-416). At the very least, they should discuss the limitations of their approach (lines 411-416).*

Response: We understand the reviewer's concern that the CagM mutants may affect protein folding and stability. During protein purification, we observed that all mutants were soluble and stable, behaving very similarly as wild-type CagM. More importantly, all mutants interacted with CagX (Supplementary Fig. 13), further suggesting that the CagM mutants retained their folding and stability.
The mutagenesis experiments aimed to identify the potential residues involved in the outer ring formation in CagM-CagX double-ring complex. In conjunction with various biophysical analysis, including crosslinking-TEM, SEC-SLS and HDX-MS, we identified various charged residues at the NTD and upstream of CTD of CagM are critical for CagM high-order oligomerization. In addition, CagM ring was not observed in these mutants. However, how these residues interact and contribute to CagM ring formation remains unclear. In this study, we do not assert that interactions between these charged residues are directly responsible for ring formation. To clarify, the limitations of our approach have been explicitly acknowledged in the discussion (line 454-457).

Minor concerns:

1) ***Comments****: Line 43 refers to a "pilus". As far as I know, this has not been established with certainty in the case of the H. pylori T4SS.*

Response: We thank the reviewer for this comment. To the best of our understanding, several studies have reported the presence of pili / pilus-like structure when *H. pylori* was co-cultured with gastric epithelial AGS cells (Backert et al., 2015; Johnson et al., 2014; Shaffer et al., 2011; Rohde et al., 2003). Johnson et al. (2014) further demonstrated that pilus formation is dependent on CagT4SS. Pilus was absent in H. pylori ΔcagPAI strain as well as individual ΔcagX, ΔcagT, ΔcagM, Δcag3 and ΔcagV knockout strains.
Other studies have reported that CagI, CagL and CagH play a role in pilus formation and regulation (Backert et al., 2015; Shaffer et al., 2011). In another independent study, extension of pilus from outer membrane-associated complex into inner membrane or cytoplasm with different depths were shown in three CagT4SS particles out of seventy

particles (Chang et al., 2018). It has also been proposed that this rare, stalled and downward protrusion assembly observed requires host contact.

In this revised manuscript, we cited two of these studies (line 55 and line 427) to support the existence of pilus substructure in *H. pylori* CagT4SS.

References

- Backert, S., Tegtmeyer, N., & Fischer, W. (2015). Composition, structure and function of the Helicobacter pylori cag pathogenicity island encoded type IV secretion system. Future microbiology, 10(6), 955-965.
- Chang, Y. W., Shaffer, C. L., Rettberg, L. A., Ghosal, D., & Jensen, G. J. (2018). In vivo structures of the Helicobacter pylori cag type IV secretion system. Cell Reports, 23(3), 673-681.
- Johnson, E. M., Gaddy, J. A., Voss, B. J., Hennig, E. E., & Cover, T. L. (2014). Genes required for assembly of pili associated with the Helicobacter pylori cag type IV secretion system. Infection and immunity, 82(8), 3457-3470.
- Shaffer, C. L., Gaddy, J. A., Loh, J. T., Johnson, E. M., Hill, S., Hennig, E. E., ... & Cover, T. L. (2011). Helicobacter pylori exploits a unique repertoire of type IV secretion system components for pilus assembly at the bacteria-host cell interface. PLoS pathogens, 7(9), e1002237.
- Rohde, M., Püls, J., Buhrdorf, R., Fischer, W., & Haas, R. (2003). A novel sheathed surface organelle of the Helicobacter pylori cag type IV secretion system. Molecular microbiology, 49(1), 219-234.

2) ***Comments:*** *Which domains of CagX, CagY and CagM were purified? This should be clarified earlier in the results section (beginning lines 91) and in the legend to Fig.1*

Response*:* We thank the reviewer for the comment. Our study began with the construction of full-length CagX and CagM lacking signal peptide, and PRC-OMCC regions of CagY (denoted as $\text{CagY}_{\text{PRC-OMCC}}$). In this revised manuscript, we have clarified this information in the beginning in the Results section (line 104-105) and in the legend of Fig. 1A.

3) ***Comments:*** *Suppl Fig. 3B particles not easy to identify. They should be indicated by arrows.*

Response: We thank the reviewer for the comment. In this revised manuscript, arrows are added to ease the identification of particles in Supplementary Fig. 3B.

4) ***Comments:*** *Microscale thermophoresis appears to be mentioned only in the Materials and methods section. Was it used in the manuscript?*

Response: Yes, microscale thermophoresis assays were performed to characterize the binding kinetics of CagX-CagY interactions. Please refer to line 331-332, 444-445 and Supplementary Fig. 19D in this revised manuscript.

5) ***Comments****: The presented assembly model is interesting, but the authors do not really consider that in vivo, CagY is a very long protein with many repeat regions. They characterized only the C-terminal membrane domains. It would be interesting*

to speculate how the N-terminal repeat regions fit into the model presented in Fig. 6.

Response: We thank the reviewer for this important comment. It has been predicted that the N-terminal region of CagY is structurally disordered. The structural model of MRR region by deep learning approaches has suggested that this region may form another ring-like structure underneath the PRC (López-Luis et al., 2023). We speculate that the stabilization of PRC-OMCC of CagY would facilitate the oligomerization and assembly of its N-terminal domains. Please refer to our revised manuscript (line 468-473).

Reference

López-Luis, M. A., Soriano-Pérez, E. E., Parada-Fabián, J. C., Torres, J., Maldonado-Rodríguez, R., & Méndez-Tenorio, A. (2023). A proposal for a consolidated structural model of the cagy protein of helicobacter pylori. International Journal of Molecular Sciences, 24(23), 16781.

6) ***Comments:*** *Considering the nature of the proteins, it is somewhat surprising that detergents were not required for the purification.*

Response: We thank the reviewer for this comment. Although CagX and CagM are mainly considered as membrane proteins, they do not contain any transmembrane region and are likely located in the periplasmic space close to the outer membrane. In our study, signal peptides of both CagX and CagM were deleted to ease the purification of the recombinant proteins from *E. coli* cytoplasm (see line 103-104, 527-528). Whereas for CagY, it contains two transmembrane regions (Fig. 1A). In our CagY construct (PRC-OMCC), only the second TM region within OMCC domain was included. CagY does not contain signal peptide. We suspected that the absence of the N-terminal region of CagY may affect the membrane targeting and localization of recombinant CagY. Noteworthy, a relatively higher salt concentration (500 mM NaCl) was used to solubilize these Cag proteins during the purification process. They were stable and did not prone to aggregation without the addition of detergents.

Reviewer #3:

***Overall comments**: I co-reviewed this manuscript with one of the reviewers who provided the listed reports. This is part of the Nature Communications initiative to facilitate training in peer review and to provide appropriate recognition for Early Career Researchers who co-review manuscripts.*

Response: We thank Reviewer 3 for co-reviewing our manuscript and for their valuable input.

Reviewer #4:

Overall comments: *The work presented by Chu et al. aims to investigate early steps in Cag T4SS assembly using in vitro reconstitution and subsequent analysis using a combination of SEC analysis, in vitro pull-down assays, single particle EM (both negative stain and cryo-EM), high speed AFM, and HDX exchange. Using recombinantly expressed and purified CagX, CagY, and CagM from E. coli, the authors are able to analyze the formation of ring-like structures that resemble the periplasmic ring of the Cag T4SS and show the propensity of CagM to homo-and hetero-oligomerize. In combination this work characterizes domains of CagM necessary for CagM-CagM oligomerization and cagM-1 radial ring assemble, finds that CagX alone can form "PR' like rings (although with more heterogenous symmetry than found in situ or purified from H. pylori and CagM-CagX can form hetero-oligomers. The HS-AFM combined with HDX exchange studies provides insights into the dynamic ability of the recombinant proteins to oligomerize. Overall, the paper was very well written and results are clearly explained. The results are interesting in that it shows the capacity of three main Cag T4SS components to form rings in isolation and as well as to assemble into larger order structures in vitro. The HDX and AFM analysis provide some of the first biophysical information about potential protein dynamics and how these proteins can behave in isolation as well as how they can interact outside of the bacterial periplasm or with other T4SS components.*

Response: We thank the reviewer for the constructive suggestions. Please refer to the response below.

Below are some questions and general comments that should be addressed and/or commented on by the authors:

1) ***Comments:*** *The authors noted that the minimum interacting construct consists of residues 67- 365 of CagM (CagMΔN49ΔC11) for binding to CagX. They propose that the assembly mechanism of CagM-CagX involves (i) self-oligomerization of the CagX ring, (ii) higher-order oligomerization of CagM, and (iii) the association of CagM to CagX. Have the authors tested CagMΔN49ΔC11 for its oligomeric state, crosslinked morphology, and ring formation with CagX?*

Response: Yes, as in other CagM mutants, we have characterized $CagM_{\Delta N49\Delta C11}$ for its oligomeric state, crosslinked morphology, and ring formation with CagX. $CagM_{\Delta N49\Delta C11}$ exhibited as a monomer in SEC-SLS and granule-like particles in DSSO-crosslinked condition under TEM. While $CagM_{\Delta N49\Delta C11}$ retained the interaction with CagX in the pull-down assay, no double ring formation was found after CagX co-incubation. These results confirm that high-order oligomerization of CagM is required for ring structure formation under DSSO treatment, and for outer ring formation in the CagM-CagX complex, supporting our proposed CagM-CagX assembly mechanism. These results have been included in line 288-290, Fig. 3A, Supplementary Fig. 14 (crosslinking morphology and oligomeric state) and 15 (ring formation with CagX) in our revised manuscript.

2) ***Comments:*** *Also, does the removal of ΔN49 cause the CagM construct to resemble the phenotype of ΔN19, which showed granule-like crosslinked morphology (indicating a loss of higher-order oligomerization of CagM) and failed to form rings with CagX, even though ΔN49 was pulled down with CagX?*

Response: *Yes, the $CagM_{\Delta N49\Delta C11}$ mutant generally resembled the phenotype of $CagM_{\Delta N19}$. Both constructs showed granule-like crosslinked morphology and failed in ring formation with CagX, aligning with our hypothesis. Please refer to our response to Comment 1 above.*

3) ***Comments:*** *Authors should show confidence of all AlphaFold predictions by color-coding the protein structures based on the per-residue pLDDT scores.*

Response: We thank the reviewer for the comment. In this revised manuscript, we have added a new figure, showing the predicted structures of PL and PI regions of CagX, CagM and PL as well as the AP regions of CagY, with their respective pLDDT scores coloured coded. Please refer to Supplementary Fig. 8.

4) ***Comments****: In Supplemental Figure 17 – why is there such a prominent high MW band in the GST control pull down? The authors should also include a lane showing the inputs (or % inputs) of the bait and prey recombinant proteins.*

Response: We thank the reviewer for the comment. That high MW band was likely an impurity in the GST control. To clarify, we have repeated the whole set of pull-down experiment and included the inputs of the bait and prey proteins in the SDS-PAGE analysis. Please refer to Supplementary Fig. 19A in the revised manuscript.

5) ***Comments****: Regarding the HS-AFM comparisons (lines 349-375) between CagM binding to immobilized CagX and CagM binding to a mixture of CagX and CagY PR-OMCC, can the authors determine the relative assembly kinetics and whether one of these samples exhibits a faster assembly rate? Have the authors also tested the incubation of immobilized CagX with CagM, followed by the addition of CagY PR OMCC?*

Response: We thank the reviewer for this valuable suggestion, which has led to one of the most significant additions to the revised manuscript. We have comprehensively quantified CagM binding and dissociation dynamics on both CagX rings and CagX-CagY rings (Fig. 6). As described in detail in our response to Reviewer #1, Comment 4 (Part 2).
In brief, on CagX-$CagY_{PRC\text{-}OMCC}$ rings, the CagM bound-state dwell time was ~1.7× longer (0.380 vs. 0.229 s) and cooperativity was ~1.6× higher (4.2× vs. 2.7×) compared to CagX-only rings (Fig. 6). As noted in our response to Reviewer #1, the absolute kinetic values should be interpreted with caution due to the inevitable perturbation from the AFM tip during continuous scanning. Nevertheless, since both conditions were measured under identical imaging parameters, the relative comparison is valid, and the results consistently indicate that CagX-$CagY_{PRC\text{-}OMCC}$ rings promote more stable CagM binding and more efficient outer ring formation.

We also performed the reverse-order experiment requested by the reviewer. $CagY_{PRC-OMCC}$ was added to pre-formed, immobilized CagM–CagX double-ring complexes (Supplementary Fig. 24B, Supplementary Movie 15). No discernible structural change was observed in the CagM–CagX double rings upon CagY addition. To verify that $CagY_{PRC-OMCC}$ was indeed present and active in the solution rather than simply absent or aggregated, we performed a temporal standard deviation (SD) analysis (Supplementary Fig. 25). The temporal SD was significantly elevated in the CagY-added group (median: 0.81 nm) compared to CagM–CagX alone (median: 0.51 nm; $p < 10^{-10}$, Mann–Whitney U test), confirming that CagY molecules were diffusing near the sample surface and transiently interacting with the AFM tip. Despite this confirmed presence of CagY, the temporal mean images remained essentially unchanged, demonstrating that CagY cannot incorporate into the pre-assembled CagM–CagX complex.
These results support the strict sequential assembly order: CagX → CagY → CagM. CagY binding requires access to the CagX ring prior to CagM association. They have been included in Results (line 385-394, 400-418), Discussion (line 463-464, 475-486) and Methodology (line 770-777, 802-815).

6) ***Comments:*** *It is not clear how the authors conclude that CagY binds to CagX before CagM; clarification on this point is needed.*

Response: This point is closely related to Comment 5 above, and the key evidence is summarized here.
Our conclusion is based on two independent lines of evidence. First, the forward sequential addition experiment (Supplementary Fig. 24A, Supplementary Movie 14) showed that triple-complex formation occurs when $CagY_{PRC-OMCC}$ is added to CagX rings before CagM, whereas the reverse order, adding $CagY_{PRC-OMCC}$ to pre-assembled CagM–CagX complexes, did not result in any structural change (Supplementary Fig. 24B, Supplementary Movie 15; see also our response to Comment 5 for the temporal SD analysis confirming that $CagY_{PRC-OMCC}$ was present but unable to incorporate). Second, this result is structurally consistent with the observation that CagM covers the OMC face of the CagX ring (Supplementary Fig. 26-27), thereby sterically occluding the CagY binding site on the PRC. Together, these functional and structural data demonstrate that $CagY_{PRC-OMCC}$ binds to CagX before CagM association.

7) ***Comments****: The first paragraph of the discussion should be tempered. Although the authors do present evidence about how these recombinant expressed proteins can assemble in vitro, this is not the same as how it assembles in situ. This should be made clearer in the wording.*

Response: We thank the reviewer for the comment. We have carefully tempered the language throughout the revised Discussion, as described in our detailed response to Reviewer #2 Comment 2. In particular, we clarified that our assembly mechanism is derived from in vitro data and discussed the possible limitations that our proposed mechanism that may not fully recapitulate in native environment. Please refer to our revised manuscript (line 428-432, 515-518).

8) ***Comments:*** *Lines 382-385: "Among diherent CagT4SS substructures, the central cylinder composing of CagX, CagY and CagM is of particular interest, as it serves as the initiator of the nanomachine, gateway for the pilus biogenesis and substrates translocation, and contact with host receptors." The authors should consider modifying this statement and/or provide more detailed references to back up these statements. Although these have been postulated functions of the central cylinder it remains controversial about whether the Cag T4SS has a pilus. For example, in the Cheng et. al. cryo-ET studies they found no evidence of a pilus associated with the Cag T4SS (PMID 29669273). The authors should also reference any experimental data showing substrates translocate through the central channel of the complex.*

Response: We thank the reviewer for the comment. To the best of our understanding, the existence of pilus/pilus-like structure in *H. pylori* CagT4SS has been reported in several publications (Backert et al., 2015; Johnson et al., 2014; Shaffer et al., 2011; Rohde et al., 2003). It has also been suggested that cagPAI and host cell contact are crucial for pilus formation. In the cryo-ET studies from Chang et al., 2018, the researchers observed a central rod structure in three unusual CagT4SS particles among 70 particles extending from outer membrane-associated complex into inner membrane or cytoplasm in the presence of AGS cells (Figure 3 in PMID 29669273). A pilus-like rod structure emerging from CagT4SS has also been proposed. In this revised manuscript, we included two representative citations to support the existence of pilus substructure in *H. pylori* CagT4SS (line 55 and line 427). Please also refer to our response to Reviewer #2 Minor Comment 1 for more details and references.
Although direct experimental evidence showing substrate translocation through the central channel of CagT4SS is not available, cumulative structural, functional and genetic studies have demonstrated the importance of the central channel in substrate translocation. For example, the cryo-EM/cryo-ET models of CagT4SS reveal a central channel spanning across from IMC to OMCC and there are ATPases located at the base of IMC that provide energy for substrate translocation (Hu et al., 2019; Sheedlo et al., 2020). A systematic mutagenesis study has shown that PRC-OMCC (CagX, CagY, CagM) and pilus components (CagH, CagL, CagI) are all required for CagA translocation (Fischer et al., 2001). Besides interacting with CagA, CagM has also been found to bind to dsDNA substrate (Bats et al., 2025). In addition, the central channels across bacterial T4SSs, including Agrobacterium tumefaciens, E. coli R388 and L. pneumophila are highly conserved (Cascales & Christie, 2004; Christie, 2004; Christie et al., 2014). Taken together, it is highly possible that H. pylori CagT4SS translocates substrate via central channel and pilus. In this revised manuscript, we have included additional references to support our statement in line 427-428.

<u>References</u>

- Bats, S. H., Metz, F., Beilmann, J., & Josenhans, C. (2025). Helicobacter pylori CagT4SS proteins CagN and CagM bind DNA and CagN is involved in heptose-independent pro-inflammatory substrate translocation by the T4SS. International Journal of Medical Microbiology, 320, 151661.
- Cascales, E., & Christie, P. J. (2004). Definition of a bacterial type IV secretion pathway for a DNA substrate. Science, 304(5674), 1170-1173.
- Christie, P. J. (2004). Type IV secretion: the Agrobacterium VirB/D4 and related conjugation systems. Biochimica et Biophysica Acta (BBA)-Molecular Cell

Research, 1694(1-3), 219-234.
- Christie, P. J., Whitaker, N., & González-Rivera, C. (2014). Mechanism and structure of the bacterial type IV secretion systems. Biochimica et Biophysica Acta (BBA)-Molecular Cell Research, 1843(8), 1578-1591.
- Fischer, W., Püls, J., Buhrdorf, R., Gebert, B., Odenbreit, S., & Haas, R. (2001). Systematic mutagenesis of the Helicobacter pylori cag pathogenicity island: essential genes for CagA translocation in host cells and induction of interleukin-8. Molecular microbiology, 42(5), 1337-1348.
- Kwok, T., Zabler, D., Urman, S., Rohde, M., Hartig, R., Wessler, S., … & Backert, S. (2007). Helicobacter exploits integrin for type IV secretion and kinase activation. Nature, 449(7164), 862-866.
- Rohde, M., Püls, J., Buhrdorf, R., Fischer, W., & Haas, R. (2003). A novel sheathed surface organelle of the Helicobacter pylori cag type IV secretion system. Molecular microbiology, 49(1), 219-234.

9) ***Comments**: In reference to lines 425-427: have the authors obtained a low-resolution 3D reconstruction of the CagX-CagM complex, or are they only discussing the 2D classes of vitrified particles shown in Fig 2A? If they are solely referencing the 2D classes, this interpretation is overstated, as the resolution is not suhicient diherentiate between "twisted and stacked pairs" of CagM versus a single CagM-2 binding to two outer radial CagM-1 monomers. If they are referring to a 3D reconstruction, this map needs to be included and presented in the manuscript.*

Response: In this revised manuscript, a 7.77 Å cryo-EM map of CagM-CagX complex (EMD-69626) is included. With this low-resolution map, some structural features of the outer rim were observed. These include the 7 pairs of intertwining spokes connecting the inner and outer rings of the complex. Please refer to our revised manuscript [Results (line 175-178), Supplementary Fig. 3B, Supplementary Table 6 and Methodology (line 676-679, 682-693)].

10) ***Comments**: Lines 436-439: The single particle cryo-EM structures of isolated Cag T4SS OMCCs and the cryo-ET analyses of the in situ CagT4SS were conducted without host cells present. In the case of the single particle structures, the OMCC, PRC, and stalk were present. In the cryo-ET analyses the OMCC, PRC, stalk, arches, and IMC were visible. These studies contradict the hypothesis that the PRC serves as the nucleation point for rapid assembly upon host cell recognition. For these reasons lines 436-439 should be reconsidered.*

Response: We thank the reviewer for this important comment. We agree with the reviewer that the PRC is unlikely to serve as the nucleation point for rapid assembly upon host contact.

Taken into consideration that the central channel is critical for substrate translocation and pilus formation, but the channel diameter is narrow (~3.5 nm facing the outer membrane and ~10 nm facing the inner membrane) (Chung et al., 2019; Sheedlo et al., 2020), we speculate that the mechanical coupling of stable PRC may act as an anchoring base, providing structural support for the flexible OMCC to undergo conformational change that may be required for the translocation of partially

unfolded CagA via the narrow channel (Letti et al., 2021; Woon et al., 2013; Wu et al., 2023). We have revised our discussion in lines 504-509.

References

- Lettl, C., Haas, R., & Fischer, W. (2021). Kinetics of CagA type IV secretion by Helicobacter pylori and the requirement for substrate unfolding. Molecular Microbiology, 116(3), 794-807.
- Woon, A. P., Tohidpour, A., Alonso, H., Saijo-Hamano, Y., Kwok, T., & Roujeinikova, A. (2013). Conformational analysis of isolated domains of Helicobacter pylori CagA. PLoS One, 8(11), e79367.
- Wu, X., Zhao, Y., Zhang, H., Yang, W., Yang, J., Sun, L., … & Wu, Y. (2023). Mechanism of regulation of the Helicobacter pylori Cagβ ATPase by CagZ. Nature communications, 14(1), 479.

11) ***Comments****: Lines: 448-451: However, the low-resolution map of the CagM-CagX complex limits the elucidation of its atomic details." What cryo-EM density map are the author's referring too?*

Response: Please refer to our response to Major Comment 9. In brief, in this revised manuscript, we have included a 7.77 Å cryo-EM map of the CagM-CagX complex.

Minor:

1) ***Comments****: Supplemental Figure 4: instead of labeling "top" and "bottom" in the figure can the authors include instead "OMC to PR" and "PR to OMC"? In the legend it should also be OMC to PR or PR to OMC (i.e. not OMCC – the OMCC is the outer membrane core complex, and I think that authors are referring to subcomplexes within the OMCC – i.e. the OMC and PR.*

Response: We thank the reviewer for the comments. We have revised the text (line 489-492) and legend of Supplementary Fig. 27 accordingly.

2) ***Comments****: Supplementary Figure 7 legend: It is unclear what "Partially resolved cryo-EM model" means. The authors should clarify. In (B) it should be Nickel-NTA pull down – not "nickel" pulldown.*

Response: Please refer to our response to Major Comment 9.
We have revised the legend of Supplementary Fig. 7B.

3) ***Comments****: Can the author's report the RMSD or a comparison of their in vitro assembled CagX-CagY PRC model and the model built from the PR in the H. pylori purified OMC-PR complex. It would be interesting to easily see any major differences.*

Response: The comparison of our in vitro C16 and C17 assembled and the in vivo isolated CagX-CagY complex was included in Supplementary Fig. 18B and discussed in line 316-318. The Cα RMSDs are 0.87Å (for C16 model) and 1.1Å (for C17 model), revealing the high structural similarities among models.

Reviewer #5:

Overall comments: *The authors have presented an intriguing manuscript that describes their efforts to study the stepwise assembly and dynamics of a nanomachine known as a virulence factor that contributes to the pathogenicity of H. pylori. Below are several comments, questions, and suggestions that can be used to improve the manuscript and ready it for publication.*

Response: We thank the reviewer for the constructive comments that improved the clarity of our manuscript. Please find our point-to-point response below.

1) ***Comments****: First, the authors have mostly conformed to the modern standard adopted by the HDX MS community for data reporting. It doesn't appear that the author have uploaded the raw MS data to a specific archive. The following will address this problem: Upload raw MS files, peptide lists, and summary processed data to the PRIDE archive, or similar. I would also suggest condensing the individual HDX supplemental data files into one workbook for ease of presentation and data mining.*

Response: We thank the reviewer for the comments. We have deposited all raw HDX-MS data of CagM-CagX complex, CagM mutants and CagX-$CagY_{PRC-OMCC}$ complex to the ProteomeXchange Consortium via the PRIDE partner repository with the dataset identifier PXD075968, PXD075969 and PXD075970, respectively. We have also combined the individual data tables (Supplementary Tables 2-5 and 7-8, separated by tabs) into one excel workbook (HDX Supp Table Combined) as suggested.

2) ***Comments****: Next, there seems to be some agreement issues with the peptide level HDX. Two examples:*
   -Supplementary Figure 5, data for peptide 305-321 seems out of place considering overlapping peptides.
   -Supplementary Figure 10, peptides 39-56 and 42-58 show conflicting data.
   This is likely due to peptide misidentification in PLGS. I would just go through and confirm peptide ID's that seem incongruent.

Response: We thank the reviewer for the comments. After manual inspection of the mentioned peptides, peaks in peptide 305-321 from Supplementary Figure 5 is now correctly placed and shows consistent deuterium uptake patterns with peptide 306-321 and 307-321. The corresponding uptake plot and deuterium uptake data have been updated in Fig. 2C, Supplementary Fig. 5 and Supplementary Table 2. Regarding the inconsistency between peptides 39-56 and 42-58 in Supplementary Fig. 10 (Supplementary Fig. 11 now), we found that spectra of several time intervals in peptide 39-56 showed ambiguous and poor signal peptide peaks, leading to the misassignment and confliction with peptide 42-58. Considering the data quality of peptide 39-56, this peptide has been removed from our analysis. The final peptide coverage (84.1%) has been also updated. Please refer to our revised manuscript for the changes (line 259, Figure 3B, Supplementary Fig. 11 and Supplementary Table 1 and Table 4).

__Comments__: The authors use increases or decreases in solvent exposure as reasons for changes in HDX. It would be better to include in this discussion decreases and increases in h-bonding strength and character as well. The HDX process requires both access to solvent and catalyst as well as the breaking of potential h-bonds to the backbone amide hydrogen in order for productive HDX to occurs

Response: We thank the reviewer for the comment. We have revised manuscript as suggested (lines 219-220, 271, 348).

3) ***Comments***: *How stable are the SEC-isolated oligomer structures? Have the SEC fractions for any of the complexes been re-injected onto the column to see if those isolated fractions have then re-equilibrated to the distributions before SEC separations?*

Response: Based on our observations, the SEC-isolated oligomeric structures were very stable. For the three independent SEC-SLS and TEM experiments, we obtained reproducible results of elution profiles and oligomeric states. As suggested by the reviewer, we have tried re-injecting CagM-CagX complex onto the same SEC column under identical conditions. The elution profile of the re-injected sample overlapped with that of the initial run (Please refer to the figure below). It is unlikely that the isolated fractions were re-equilibrated after SEC separations.

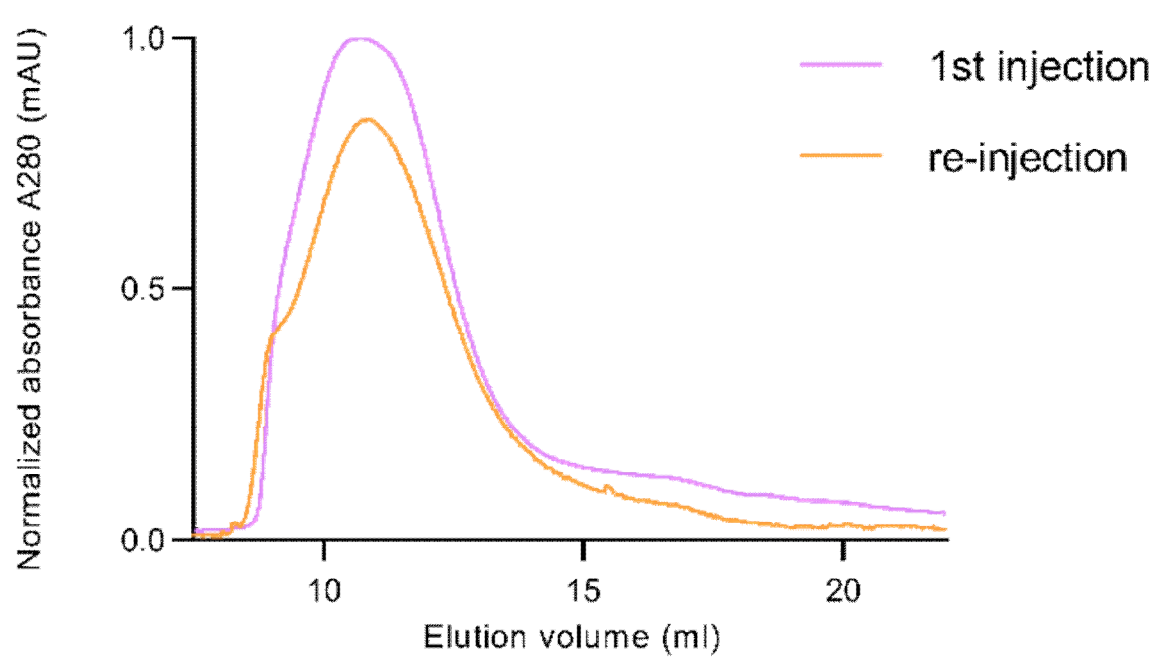

Gel filtration elution profiles of CagM-CagX complex, first injection (pink) and re-injection (orange)

4) ***Comments***: *Along those same lines, was it possible to see distributions/pouplations in the isotopic envelopes in the raw HDX data that reflect the range in oligomerization states that are reported from the cryo-EM data? It would be curious if there were differences in the HDX of the monomeric units depending on the oligomerization sate.*

Response: We agree that heterogeneous oligomeric states may manifest as multimodal isotopic envelopes in HDX-MS data. However, in our experiments, peptides at and around the oligomerization interface exhibited single, gradually shifting EX2 envelopes and we did not observe any multimodal features or populations that could be assigned to different oligomeric states. This observation is consistent with previously resolved cryo-EM model and our CagX-CagY$_{PRC-OMCC}$ cryo-EM

structures, showing that the complexes share essentially the same interface in all protomers, that they displayed similar HDX patterns regardless of the oligomeric states. As a result, the HDX differences depending on oligomerization states are expected to be negligible.

5) ***Comments**: Line 115: "…are composed of arrowhead-like units…". I think it would be more accurate to describe the shape as a 3-lobed epitrochoid or reuleaux-like triangle.*

Response: We have revised to "reuleaux-like triangle" in our revised manuscript (see line 126).

6) ***Comments**: Line 192 and 255: instead of "overlapping" perhaps consider using coincident peptides. I think that overlapping is confusing when it is also used to describe peptides within a protein construct.*

Response: We thank the reviewer for the comment. We have revised the statement accordingly in our revised manuscript (see line 198 and 259).

7) ***Comments**: Line 258: "…may be due to their differences in oligomeric states, resulting in distinctive pepsin digestion patterns." I think that this is only half-correct. At the point of digestion, the protein sample is quenched to low pH and temperature. SO, differences in the acid denatured states that arrive from the different oligomeric states could be the reason for the differences in digestion patterns.*

Response: We thank the reviewer for the comment. We have revised the statement accordingly in our revised manuscript (line 262).

RESPONSE TO REVIEWERS' COMMENTS

Reviewer #1:

The authors have performed additional HS-AFM experiments and quantitative anaylsis, which significantly strengthened this work. All of my previous concerns have been addressed. I now fully support the publication of this work.

Response: We thank the reviewer for the thorough evaluation of our manuscript and for supporting its publication in *Nature Communications*.

Reviewer #2:

The authors have carefully addressed the reviewers's suggestions and I have no additional comments. They should be commended for this carefully executed study and for their detailed and constructive response to all reviewer comments.

Response: We are grateful to the reviewer for the positive comments.

Reviewer #3:

I co-reviewed this manuscript with one of the reviewers who provided the listed reports. This is part of the Nature Communications initiative to facilitate training in peer review and to provide appropriate recognition for Early Career Researchers who co-review manuscripts.

Response: We thank the reviewer for co-reviewing our manuscript and for their valuable input.

Reviewer #4:

The authors were very responsive to the reviews. They have addressed my questions and I appreciate the care and effort they went about in their response and edits. I cannot comment the HDX or AFM revisions since this is not my area of expertise.

The only thing I feel needs to be addressed in the revision is that the authors should add the FSC curve and Euler angle plot to supplemental figure 3 and an Euler angle plot to supplemental figure 17. If there are any 2D averages that are not a "top" or "bottom" views - these would also be helpful to include - but the Euler plots should be sufficient to show the extent (or not) of preferred orientation. I apologize for not requesting the change to the current supplemental 17 in the first revision.

Response: We sincerely appreciate the reviewers' suggestions. We have included the FSC curve and 2D angular distribution plot for CagM-CagX in Supplementary Figure 3b. An 2D angular distribution plot for CagX-$CagY_{PRC-OMCC}$ has also been added to Supplementary Figure 17.

Reviewer #5:

I thank the authors for taking the time to address my "comments, questions, and suggestions". I have nothing more to add, and I cannot wait to see the final version in press.

Response: We are grateful for the constructive comments from Reviewer 5 and for supporting the publication of our work in *Nature Communications*.

Reviewer 4 Review Attachment 1

The work presented by Chu et al. aims to investigate early steps in Cag T4SS assembly using in vitro reconstitution and subsequent analysis using a combination of SEC analysis, in vitro pull-down assays, single particle EM (both negative stain and cryo-EM), high speed AFM, and HDX exchange. Using recombinantly expressed and purified CagX, CagY, and CagM from E. coli, the authors are able to analyze the formation of ring-like structures that resemble the periplasmic ring of the Cag T4SS and show the propensity of CagM to homo- and hetero-oligomerize. In combination this work characterizes domains of CagM necessary for CagM-CagM oligomerization and cagM-1 radial ring assemble, finds that CagX alone can form "PR' like rings (although with more heterogenous symmetry than found in situ or purified from H. pylori and CagM-CagX can form hetero-oligomers. The HS-AFM combined with HDX exchange studies provides insights into the dynamic ability of the recombinant proteins to oligomerize. Overall, the paper was very well written and results are clearly explained. The results are interesting in that it shows the capacity of three main Cag T4SS components to form rings in isolation and as well as to assemble into larger order structures in vitro. The HDX and AFM analysis provide some of the first biophysical information about potential protein dynamics and how these proteins can behave in isolation as well as how they can interact outside of the bacterial periplasm or with other T4SS components. Below are some questions and general comments that should be addressed and/or commented on by the authors:

1. The authors noted that the minimum interacting construct consists of residues 67-365 of CagM (CagMΔN49ΔC11) for binding to CagX. They propose that the assembly mechanism of CagM-CagX involves (i) self-oligomerization of the CagX ring, (ii) higher-order oligomerization of CagM, and (iii) the association of CagM to CagX. Have the authors tested CagMΔN49ΔC11 for its oligomeric state, crosslinked morphology, and ring formation with CagX?
2. Also, does the removal of ΔN49 cause the CagM construct to resemble the phenotype of ΔN19, which showed granule-like crosslinked morphology (indicating a loss of higher-order oligomerization of CagM) and failed to form rings with CagX, even though ΔN49 was pulled down with CagX?
3. Authors should show confidence of all AlphaFold predictions by color coding color-coding the protein structures based on the per-residue pLDDT scores.
4. In Supplemental Figure 17 – why is there such a prominent high MW band in the GST control pull down? The authors should also include a lane showing the inputs (or % inputs) of the bait and prey recombinant proteins.
5. Regarding the HS-AFM comparisons (lines 349-375) between CagM binding to immobilized CagX and CagM binding to a mixture of CagX and CagY PR-OMCC, can the authors determine the relative assembly kinetics and whether one of these

samples exhibits a faster assembly rate? Have the authors also tested the incubation of immobilized CagX with CagM, followed by the addition of CagY PR-OMCC?

6. It is not clear how the authors conclude that CagY binds to CagX before CagM; clarification on this point is needed.
7. The first paragraph of the discussion should be tempered. Although the authors do present evidence about how these recombinant expressed proteins can assemble in vitro, this is not the same as how it assembles in situ. This should be made clearer in the wording.
8. Lines 382-385: "Among different CagT4SS substructures, the central cylinder composing of CagX, CagY and CagM is of particular interest, as it serves as the initiator of the nanomachine, gateway for the pilus biogenesis and substrates translocation, and contact with host receptors." The authors should consider modifying this statement and/or provide more detailed references to back up these statements. Although these have been postulated functions of the central cylinder it remains controversial about whether the Cag T4SS has a pilus. For example, in the Cheng et. al. cryo-ET studies they found no evidence of a pilus associated with the Cag T4SS (PMID 29669273). The authors should also reference any experimental data showing substrates translocate through the central channel of the complex.
9. In reference to lines 425-427: have the authors obtained a low-resolution 3D reconstruction of the CagX-CagM complex, or are they only discussing the 2D classes of vitrified particles shown in Fig 2A? If they are solely referencing the 2D classes, this interpretation is overstated, as the resolution is not sufficient differentiate between "twisted and stacked pairs" of CagM versus a single CagM-2 binding to two outer radial CagM-1 monomers. If they are referring to a 3D reconstruction, this map needs to be included and presented in the manuscript.
10. Lines 436-439: The single particle cryo-EM structures of isolated Cag T4SS OMCCs and the cryo-ET analyses of the in situ CagT4SS were conducted without host cells present. In the case of the single particle structures, the OMCC, PRC, and stalk were present. In the cryo-ET analyses the OMCC, PRC, stalk, arches, and IMC were visible. These studies contradict the hypothesis that the PRC serves as the nucleation point for rapid assembly upon host cell recognition. For these reasons lines 436-439 should be reconsidered.
11. Lines: 448-451: However, the low-resolution map of the CagM-CagX complex limits the elucidation of its atomic details." What cryo-EM density map are the author's referring too?

Minor:

1. Supplemental Figure 4: instead of labeling “top” and “bottom” in the figure can the authors include instead “OMC to PR” and “PR to OMC”? In the legend it should also be OMC to PR or PR to OMC (i.e. not OMCC – the OMCC is the outer membrane core complex, and I think that authors are referring to subcomplexes within the OMCC – i.e. the OMC and PR.
2. Supplementary Figure 7 legend: It is unclear what “Partially resolved cryo-EM model” means. The authors should clarify. In (B) it should be Nickel-NTA pull down – not “nickel” pulldown.
3. Can the author’s report the RMSD or a comparison of their in vitro assembled CagX-CagY PRC model and the model built from the PR in the *H. pylori* purified OMC-PR complex. It would be interesting to easily see any major differences.
