# Supplementary material for: Deciphering the initiation pathway of CagT4SS assembly by in vitro reconstitution of subcomplexes
Source: Nat Commun. 2026 Jul 23;17:8987. doi: 10.1038/s41467-026-75820-0 (PMC13507270; doi:10.1038/s41467-026-75820-0)
Supplement: Supplementary file 4 [file 41467_2026_75820_MOESM2_ESM.pdf]

Description of Additional Supplementary Files

Supplementary Data

Supplementary Data 1. HDX data table for local HDX-MS of CagX.

Supplementary Data 2. HDX data table for local HDX-MS of CagM.

Supplementary Data 3. HDX data table for local HDX-MS of $CagM_{NTD-EK}$.

Supplementary Data 4. HDX data table for local HDX-MS of $CagM_{DEK}$.

Supplementary Data 5. HDX data table for local HDX-MS of CagX.

Supplementary Data 6. HDX data table for local HDX-MS of $CagY_{PRC-OMCC}$.

Supplementary Movies

Supplementary Movie 1. HS-AFM movie of CagX monomer on bare mica, acquired with the continuous scanning mode at an imaging speed of 0.1 s/frame. Data presented in Supplementary Fig. 2A.

Supplementary Movie 2. HS-AFM movie of $CagY_{PRC-OMCC}$ monomer on APTES-treated mica, acquired with the continuous scanning mode at an imaging speed of 0.08 s/frame. Data presented in Supplementary Fig. 2B.

Supplementary Movie 3. HS-AFM movie of CagM monomer observation with height profile on bare mica, acquired with the continuous scanning mode at an imaging speed of 0.1 s/frame. Data presented in Supplementary Fig. 2D.

Supplementary Movie 4. HS-AFM movie of adsorption process of CagM onto APTES-treated mica, acquired with the continuous scanning mode at an imaging speed of 1 s/frame. Data presented in Fig. 1F.

Supplementary Movie 5. HS-AFM movie of adding CagM into CagX pre-immobilized on APTES-treated mica, acquired with the continuous scanning mode at an imaging speed of 1 s/frame. Data presented in Supplementary Fig. 4.

Supplementary Movie 6. HS-AFM movie of adding CagM into CagX pre-immobilized on APTES-treated mica, acquired with the intermittent scanning mode (1 frame on, 3 frames off) at an imaging speed of 0.5 s/frame. Data presented in Fig. 2B.

Supplementary Movie 7. HS-AFM movie of adding wild-type CagM into CagX ring, acquired with the intermittent scanning mode (1 frame on, 5 frames off) at an imaging speed of 0.15 s/frame. Data presented in Fig. 3F.

Supplementary Movie 8. HS-AFM movie of adding $CagM_{NTD-EK}$ mutant into CagX ring, acquired with the intermittent scanning mode (1 frame on, 5 frames off) at an imaging speed of 0.15 s/frame. Data presented in Fig. 3F.

Supplementary Movie 9. HS-AFM movie of adding $CagM_{DEK}$ mutant into CagX ring, acquired with the intermittent scanning mode (1 frame on, 5 frames off) at an imaging speed of 0.15 s/frame. Data presented in Fig. 3F.

Supplementary Movie 10. HS-AFM movie comparing the structural stability of CagX versus CagX-$CagY_{PRC\text{-}OMCC}$ rings over time, acquired with the continuous scanning mode at an imaging speed of 0.15 s/frame. Data presented in Supplementary Fig. 22.

Supplementary Movie 11. HS-AFM movie of adding CagM to CagX-$CagY_{PRC\text{-}OMCC}$ complex on bare mica, demonstrating direct outer ring formation, acquired with the continuous scanning mode at an imaging speed of 1 s/frame. Data presented in Fig. 5A.

Supplementary Movie 12. HS-AFM movie of CagM binding and dissociation dynamics on CagX-$CagY_{PRC\text{-}OMCC}$ ring, acquired with the continuous scanning mode at an imaging speed of 0.15 s/frame. Data presented in Fig. 5B.

Supplementary Movie 13. HS-AFM movie demonstrating sequential addition of CagX, followed by $CagY_{PRC\text{-}OMCC}$, and then CagM, acquired initially with the intermittent scanning mode (1 frame on, 5 frames off) and subsequently with the continuous scanning mode at an imaging speed of 1 s/frame. Data presented in Supplementary Fig. 24A.

Supplementary Movie 14. HS-AFM reverse-order experiment movie showing $CagY_{PRC\text{-}OMCC}$ added to pre-immobilized CagM-CagX complex, acquired with the continuous scanning mode at an imaging speed of 0.5 s/frame. Data presented in Supplementary Fig. 24B.

Supplementary Movie 15. HS-AFM movie for temporal SD analysis comparing CagM-CagX + CagY versus CagM-CagX only, acquired with the continuous scanning mode at an imaging speed of 0.2 s/frame. Data presented in Supplementary Fig. 25.

Supplementary Movie 16. HS-AFM movie of adding $CagM_{DEK}$ mutant to CagX-$CagY_{PRC\text{-}OMCC}$ ring, resulting in no intact outer ring formation, acquired with the intermittent scanning mode (1 frame on, 5 frames off) at an imaging speed of 1 s/frame. Data presented in Fig. 5C.

Supplementary Movie 17. HS-AFM movie of adding CagM into CagX pre-immobilized on bare mica, showing the failure of outer ring formation, acquired with the intermittent scanning mode (1 frame on, 5 frames off) at an imaging speed of 1 s/frame. Data presented in Supplementary Fig. 26C.

Supplementary Movie 18. HS-AFM movie of adding CagM to CagX-$CagY_{PRC\text{-}OMCC}$ complex on APTES-treated mica, showing the failure of outer ring formation due to reversed orientation, acquired with the intermittent scanning mode (1 frame on, 5 frames off) at an imaging speed of 1 s/frame. Data presented in Supplementary Fig. 26F.
